# Downregulation of stromal syntenin sustains AML development

**DOI:** 10.15252/emmm.202317570

**Published:** 2023-10-11

**Authors:** Raphael Leblanc, Rania Ghossoub, Armelle Goubard, Rémy Castellano, Joanna Fares, Luc Camoin, Stephane Audebert, Marielle Balzano, Berna Bou‐Tayeh, Cyril Fauriat, Norbert Vey, Sylvain Garciaz, Jean‐Paul Borg, Yves Collette, Michel Aurrand‐Lions, Guido David, Pascale Zimmermann

**Affiliations:** ^1^ Team Spatio‐Temporal Regulation of Cell Signaling‐Scaffolds and Phosphoinositides, Equipe Labellisée Ligue 2018, Centre de Recherche en Cancérologie de Marseille (CRCM) Institut Paoli‐Calmettes, Aix‐Marseille Université, Inserm, CNRS Marseille France; ^2^ TrGET Preclinical Platform, Centre de Recherche en Cancérologie de Marseille, Inserm, CNRS Aix‐Marseille Université, Institut Paoli‐Calmettes Marseille France; ^3^ Proteomics and Mass Spectrometry Platform, Centre de Recherche en Cancérologie de Marseille Aix‐Marseille Université, Inserm, CNRS, Institut Paoli Calmettes Marseille France; ^4^ Team Immunity and Cancer, Centre de Recherche en Cancérologie de Marseille Aix‐Marseille Université, Inserm, CNRS, Institut Paoli Calmettes Marseille France; ^5^ Aix‐Marseille Univ, Inserm, CNRS, Institut Paoli‐Calmettes, CRCM Marseille France; ^6^ Team Leuko/Stromal Interactions in Normal and Pathological Hematopoiesis, Centre de Recherche en Cancérologie de Marseille, Aix‐Marseille Université, Inserm, CNRS, Institut Paoli Calmettes Marseille France; ^7^ Department of Human Genetics K U Leuven Leuven Belgium

**Keywords:** cell‐to‐cell communication, syntenin, tumor aggressiveness, tumor–stroma, Cancer, Haematology

## Abstract

The crosstalk between cancer and stromal cells plays a critical role in tumor progression. Syntenin is a small scaffold protein involved in the regulation of intercellular communication that is emerging as a target for cancer therapy. Here, we show that certain aggressive forms of acute myeloid leukemia (AML) reduce the expression of syntenin in bone marrow stromal cells (BMSC). Stromal syntenin deficiency, in turn, generates a pro‐tumoral microenvironment. From serial transplantations in mice and co‐culture experiments, we conclude that syntenin‐deficient BMSC stimulate AML aggressiveness by promoting AML cell survival and protein synthesis. This pro‐tumoral activity is supported by increased expression of endoglin, a classical marker of BMSC, which *in trans* stimulates AML translational activity. In short, our study reveals a vicious signaling loop potentially at the heart of AML–stroma crosstalk and unsuspected tumor‐suppressive effects of syntenin that need to be considered during systemic targeting of syntenin in cancer therapy.

## Graphical abstract

The lack of syntenin in the tumor microenvironment, particularly in bone marrow stromal cells (BMSC), enhances acute myeloid leukemia (AML) aggressiveness.

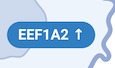

**The paper explained**

**Problem**

The crosstalk between cancer cells and their microenvironment is essential for sustained tumor progression. We currently lack insight into the molecular mechanisms supporting this crosstalk.

**Results**

We illustrate that aggressive acute myeloid leukemia (AML) blasts are able to reduce the expression of syntenin, a small PDZ scaffold protein, in bone marrow stromal cells (BMSC), by transferring miR‐155, an oncogene upregulated in 30% of human AML cases. Stromal syntenin deficiency, in turn, generates a pro‐tumoral microenvironment that stimulates AML cell survival and protein synthesis. This activity is supported by the accumulation of endoglin in BMSC, which *in trans* induces high EEF1A2 protein levels and translational activity in AML blasts.

**Impact**

Studying the communication between cancerous blasts and BMSC, we here identified a vicious circle of intercellular communication at work in aggressive forms of AML. These findings may help better patient stratification and rational therapeutic intervention.

## Introduction

Acute myeloid leukemia (AML) is a heterogeneous malignancy that accounts for over 80% of all acute leukemias in adults, with a 5‐year overall survival rate below 30% (Döhner *et al*, 2017). This disease is characterized by the clonal expansion of immature myeloblasts, originating from leukemic stem cells (LSC) which are niched within the bone marrow (BM; Morrison & Scadden, 2014). The crosstalk between AML cells and surrounding bone marrow stromal cells (BMSC) is essential to sustain tumor progression. Indeed, the leukemic blasts progressively hijack the BMSC to disrupt normal hematopoiesis and promote their own growth and survival (Ayala *et al*, 2009; Shiozawa & Taichman, 2010; Duarte *et al*, 2018; Ghobrial *et al*, 2018). BMSC‐mediated protection of leukemic cells relies on leuko‐stromal interactions mediated by ligand–receptor pairs involving adhesion molecules, cytokines, chemokines, growth factors, and metabolites. Extracellular vesicles (including exosomes) and mitochondrial transfers also emerged as important vehicles for this cellular crosstalk (Moschoi *et al*, 2016; Kumar *et al*, 2018). Identifying specific mechanisms that coordinate the AML–stroma crosstalk can potentially help refine diagnostics and anticancer therapies.

Intriguingly, transcriptomic analysis of BMSC isolated from mice inoculated with different subtypes of AML has revealed downregulation of a protein called syntenin (Battula *et al*, 2017). In prior work, we identified syntenin as a small PDZ scaffold protein operating in membrane trafficking. It acts as a rate‐limiting factor in cell‐to‐cell communication, supporting both *in cis* and *in trans* signaling. Indeed, syntenin works at the cross‐section of pathways that mediate the cell surface recycling of endocytosed receptor complexes (Zimmermann *et al*, 2005; Egea‐Jimenez *et al*, 2016) versus their exosomal secretion after budding into multivesicular bodies (Baietti *et al*, 2012; Ghossoub *et al*, 2014; Friand *et al*, 2015). Syntenin directly binds to multiple transmembrane proteins by virtue of its PDZ domains (Grootjans *et al,* 1997; Beekman & Coffer, 2008; Luyten *et al*, 2008). For instance, by interacting with syndecans, syntenin controls the recycling and exosomal secretion of FGF signals (Zimmermann *et al*, 2005; Baietti *et al*, 2012), known drivers of cancer cell proliferation and survival (Karajannis *et al*, 2006). Syntenin–syndecan also drives non‐cell‐autonomous SRC oncogenic effects mediated by exosomes (Imjeti *et al*, 2017). Currently, syntenin gain of function is strongly associated with pro‐tumoral effects and has been proposed as a therapeutic target (Pradhan *et al*, 2020). Several inhibitors have already been developed for that purpose and tested in preclinical models (Kegelman *et al*, 2017; Leblanc *et al*, 2020; Pradhan *et al*, 2021). Yet, whether and how stromal syntenin might influence tumoral cell behavior, in particular in AML, is still unknown.

Here, we investigate the consequence of stromal syntenin downregulation for AML progression. Using both *in vivo* and *in vitro* approaches and various models of AML, we found that lack of syntenin in the tumor environment, in particular in BMSC, ultimately promotes AML aggressiveness. Exploring the molecular mechanisms at work, both in AML blasts and BMSC, we identified a signaling loop potentially at the heart of stroma‐induced tumor survival. This vicious loop is fed by imbalanced stromal endoglin expression and localization, sustained AML EEF1A2/AKT/RPS6 signaling, and possibly miR‐155 transfer from AML to BMSC.

## Results

### Aggressive AML cells downregulate stromal syntenin expression

**Figure 1:**
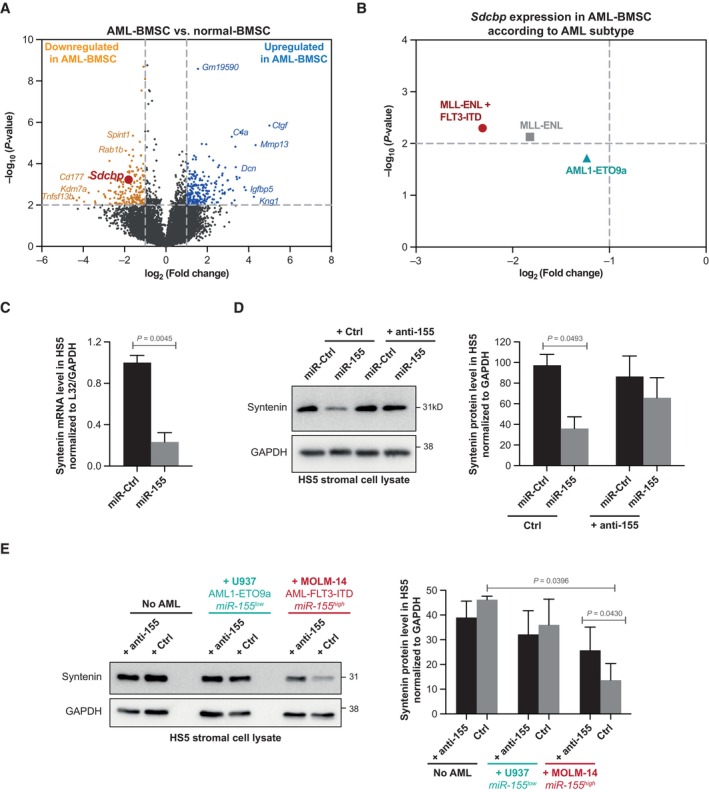
Aggressive human FLT3‐ITD^+^ AML downregulates stromal syntenin expression through miR‐155 The volcano plot was generated from the publicly available database (GSE97194). It illustrates the changes in gene expression, measured by microarray, with log_2_‐fold change (x‐axis) and −log_10_ (*P*‐value) (y‐axis), in BMSC isolated from C57BL/6 mice inoculated with AML (AML‐BMSC) compared to BMSC isolated from control animals (normal‐BMSC). Genes, down‐ or upregulated in AML‐BMSC compared to normal BMSC, were selected based on a *P*‐value < 0.01 and a difference > 2 (see Dataset EV1 for raw data).Dot plot representing *Sdcbp* expression in AML‐BMSC, compared to normal BMSC, according to the AML subtypes inoculated in the animals (MLL‐ENL + FLT3‐ITD, MLL‐ENL, and AML1‐ETO9a). The selected threshold is based on a *P*‐value < 0.01 and a difference > 2 (see Dataset EV2 for raw data).Real‐time PCR analysis of syntenin mRNA expression in HS5 transfected with hsa‐miR‐155‐5p (miR‐155; 30 nM) or control (miR‐Ctrl). Values were normalized to housekeeping L32/GAPDH genes. Data represent the mean ± SEM of three independent experiments performed in duplicate. Statistical analysis was performed using Student's *t*‐test.Left, western blots on cell lysates from HS5 cells transfected with hsa‐miR‐155‐5p (miR‐155) or control (miR‐Ctrl), in the presence or absence of miR‐155‐inhibitor (anti‐155), illustrating the miR‐155‐dependent loss of syntenin expression. GAPDH is used as loading control. Right, histograms representing mean syntenin signal intensities ± SEM, relative to GADPH, calculated from the analysis of three independent experiments. Statistical analysis was performed using the one‐way analysis of variance (ANOVA).Left, western blots of total cell lysates from HS5 transfected with miR‐155 inhibitor (anti‐155) or control, cultured in the absence or presence of U937 (AML‐ETO, miR‐155^low^) or MOLM14 (AML‐FLT3‐ITD, miR‐155^high^) cells for 24 h, illustrating the impact on syntenin signals. GAPDH was used as loading control. Right, histograms representing mean signal intensities ± SEM, relative to loading control, calculated from the analysis of three independent experiments. Statistical analysis was performed using the one‐way analysis of variance (ANOVA). Source data are available online for this figure.

By screening a publicly available database (GSE97194) previously established by Battula *et al* (2017), we found that syntenin expression is downregulated in BMSC isolated from mice with leukemia (Fig 1A; Dataset EV1). In this study, BMSC from C57Bl/6 mice transplanted with murine AML cells harboring different genetic alterations (MLL‐ENL, MLL‐ENL + FLT3‐ITD, and AML1‐ETO9a) were isolated by FACS and their gene expression profiles were established by microarray analysis (Battula *et al*, 2017). The selected threshold revealed 241 upregulated (blue dots) and 187 downregulated (orange dots) genes in BMSC stromal cells isolated from mice with leukemia (AML‐BMSC) compared to normal BMSC, isolated from nontransplanted animals (Fig 1A). Among those figured *Sdcbp* (the gene encoding syntenin), whose average expression in BMSC, across various AML genotypes, was 3.2‐fold downregulated (Fig 1A). Noteworthy, compared to other AML subtypes, AML cells bearing MLL‐ENL + FLT3‐ITD mutations seem to be more prone to reduce syntenin expression in BMSC (Fig 1B; Dataset EV2). Interestingly, the AML FLT3‐ITD subtype was shown to overexpress miR‐155, an oncogene known to accelerate AML progression (Gerloff *et al*, 2015; Salemi *et al*, 2015). Moreover, recent “omic” studies revealed that this miRNA directly downregulates syntenin expression (Xu *et al*, 2010; Lößner *et al*, 2011; Lopez‐Ramirez *et al*, 2014). To validate that stromal syntenin is indeed a miR‐155 target, we first transfected human miR‐155 in the human stromal cell line HS5. As expected, we found that miR‐155 can reduce syntenin mRNA and protein, by 80 and 60%, respectively (Fig 1C and D). Of note, when co‐transfecting HS5 cells with miR‐155 and its inhibitor (anti‐miR‐155), syntenin expression was no longer affected, illustrating the specificity of the effects (Fig 1D). To test whether human AML cells overexpressing miR‐155 can act *in trans* to downregulate stromal syntenin, we co‐cultured FLT3‐ITD (miR‐155^high^) MOLM‐14 and AML1‐ETO9a (miR‐155^low^) U937 cells with HS5 cells (Gerloff *et al*, 2015; Salemi *et al*, 2015). After 24 h of co‐culture, stromal syntenin levels remained unaffected in the presence of U937 (miR‐155^low^) cells, while MOLM‐14 (miR‐155^high^) markedly decreased stromal syntenin expression (Fig 1E). Transfecting the stromal cells with miR‐155 inhibitor prevented the loss of stromal syntenin induced by MOLM‐14 (miR‐155^high^) cells, confirming the downregulation of syntenin is indeed mediated by miR‐155 (Fig 1E). Altogether, these results indicate that syntenin expression in the microenvironment, in particular in the BMSC, can be modulated by AML cells and that stromal syntenin might be downregulated by AML expressing high levels of miR‐155.

### Host syntenin deficiency enhances AML aggressiveness

**Figure 2:**
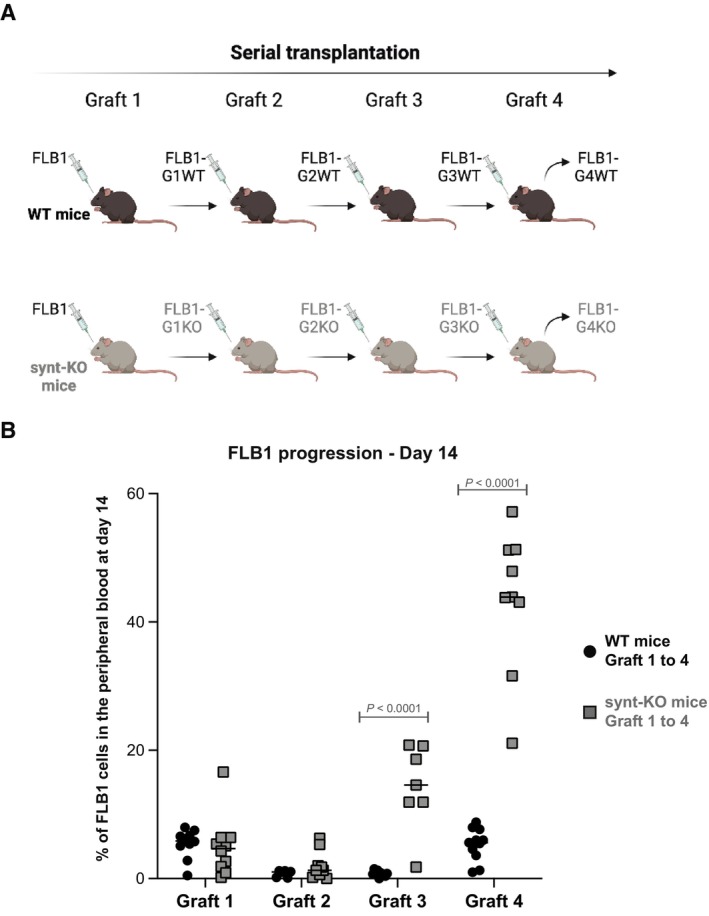
FLB1 blasts gain in aggressiveness upon serial transplantation in a syntenin‐deficient host Scheme illustrating the serial transplantation assay. Briefly, WT and syntenin knock‐out (synt‐KO) mice were injected, in parallel, with FLB1 cells. Upon complete invasion of the bone marrow, FLB1 cells were harvested and re‐injected for a new round of transplantation. FLB1 were serially transplanted into WT and synt‐KO animals in parallel for four rounds (graft 1 to graft 4)FACS analysis of blood samples collected from mice on day 14 post‐injection. Leukemia burden is expressed as percentage of FLB1 (CD45.1^+^) cells relative to total CD45^+^ (CD45.1^+^+CD45.2^+^) in the peripheral blood ± SEM. Statistical analysis was performed using the two‐way analysis of variance (ANOVA). Note the stability of the leukemia burden upon serial transplantation in WT animals. Note leukemia “outbreak,” from graft 3 on, in synt‐KO animals. Source data are available online for this figure.

To address the role of host syntenin in AML development, we made use of the murine FLB1 model that nicely recapitulates features of human AML. FLB1 cells overexpress the oncogenes Meis1 and Hoxa9 and are mir‐155^low^ (Wilhelm *et al*, 2011). This model rapidly produces widespread AML when inoculated into syngeneic C57Bl/6J mice; the frequency of leukemic stem cells remaining stable over successive transplantations (Wilhelm *et al*, 2011). Of note, FLB1 cells are CD45.1, allowing unambiguous identification of the leukemic blasts versus host cells when engrafted in CD45.2 mice. At graft 1, loss of host syntenin had no noticeable effect on FLB1 progression (Appendix Fig S1A). In parallel short‐term homing assays, lack of host syntenin had also no significant effect on the recruitment of the leukemic cells to the primary and secondary hematopoietic organs (Appendix Fig S1A). As expected, FLB1 growth in wild‐type (WT) animals remained stable over successive transplantations (Fig 2). Yet, FLB1 grown in syntenin‐knockout (synt‐KO) mice significantly gained in aggressiveness from the third transplantation on (Fig 2B) invading bone marrow and spleen and escaping in the peripheral blood within 2 weeks instead of 3 (Appendix Fig S1C). Altogether, our data reveal that AML blasts confronted with a syntenin‐deficient microenvironment ultimately acquire an aggressive phenotype.

### AML blasts educated in a syntenin‐deficient host display enhanced survival and protein synthesis

**Figure 3:**
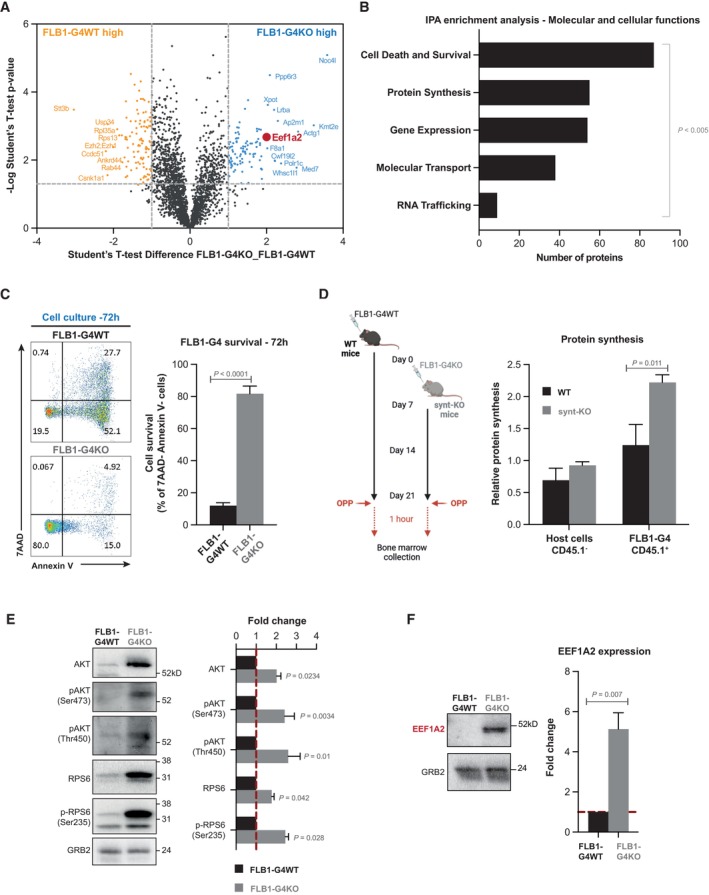
Education by a syntenin‐deficient host sustains cell survival and protein synthesis in leukemia blasts Volcano plot illustrating the proteins that are differentially expressed, with log_10_ levels (x‐axis) and –log (*P*‐value) (y‐axis), in FLB1‐G4KO (aggressive) blasts versus FLB1‐G4WT blasts (see Dataset EV3 for raw data).Molecular and cellular functions identified using ingenuity pathway analysis (IPA) based on the selected threshold (*P* < 0.005; difference > 1). The bar diagram indicates the most significant molecular and cellular functions upregulated in FLB1 cells transplanted for four rounds in Synt‐KO mice compared to FLB1 cells transplanted in WT animals. The x‐axis indicates the number of molecules involved in the corresponding y‐axis function.FACS analysis of FLB1 (CD45.1^+^) cells collected after graft 4 from WT (FLB1‐G4WT) or synt‐KO animals (FLB1‐G4KO). Apoptosis was evaluated after 72 h of maintenance in complete RPMI media, using double staining with 7AAD/AnnexinV, as illustrated on the representative dot blots (Left panel). Results from three independent experiments are expressed as mean value of living (AnnexinV^−^, 7AAD^−^) FLB1cells ± SEM. Statistical analyses were performed using the nonparametric Mann–Whitney *U*‐test (Right panel).Left, scheme of the experiment. FLB1‐G4WT cells were injected at day 0 into WT mice, while the inoculation of FLB1‐G4KO cells into synt‐KO mice was delayed for 1 week to obtain similar stages of disease progression in the two different hosts at day 21. To address *in vivo* protein synthesis, O‐propargyl‐puromycin (OPP) was injected intraperitoneally. One hour later, BM was collected and OPP incorporated into nascent polypeptide chains was fluorescently labeled via “Click‐it Chemistry.” Right, graph representing the relative levels of protein synthesis in host cells (CD45.1‐) and in FLB1 cells (CD45.1^+^) relative to the BM cells from OPP‐untreated mice ± SEM as measured by FACS and calculated from the analysis of three independent mice. Statistical analysis was performed using the nonparametric Mann–Whitney *U*‐test.Left, western blots of FLB1‐G4WT and ‐G4KO total cell lysates analyzed for different markers, as indicated. Right, histograms representing FLB1‐G4KO mean signal intensities ± SEM, relative to signals obtained with FLB1‐G4WT lysates, calculated from the analysis of five independent mice. Statistical analysis was performed using the nonparametric Mann–Whitney *U*‐test.Left, western blot from FLB1‐G4WT and ‐G4KO total cell lysates illustrating EEF1A2 expression. The ubiquitous GRB2 signal was used as loading control. Right, histogram representing FLB1‐G4KO EEF1A2 mean signal intensities ± SEM, relative to signals obtained with FLB1‐G4WT lysates, calculated from the analysis of five independent mice. Statistical analysis was performed using the nonparametric Mann–Whitney *U*‐test. Source data are available online for this figure.

To identify the mechanisms supporting this AML aggressiveness, we first assessed classical “hallmarks” associated with leukemia progression. As shown in Appendix Fig S2A–C, we excluded an altered homing, change in leukemic initiating cell frequencies or cell cycle defect. We then compared the proteomes of FLB1 blasts isolated from WT and synt‐KO animals after graft 4 (further referred to as FLB1‐G4WT and FLB1‐G4KO, respectively). Quantitative protein expression analysis by mass spectrometry allowed the reproducible identification of 2,705 proteins (Dataset EV3). Figure 3A shows a volcano plot of the entire dataset, highlighting proteins whose expressions were significantly different within FLB1‐G4WT and G4KO cells. The selected thresholds revealed 104 upregulated (blue dots) and 112 downregulated (orange dots) proteins in FLB1‐G4 isolated from synt‐KO animals. Bioinformatic evaluation of these changes (i.e., ingenuity pathway analysis) identified “Cell Death and Survival” and “Protein Synthesis” pathways, as the top two molecular and cellular functions that were significantly different (Fig 3B; Dataset EV3). To validate these observations, we first tested the capacity of FLB1‐G4 cells to survive *ex vivo*. FLB1‐G4 cells were isolated from bone marrow aspirates by FACS and seeded in complete medium. We found that FLB1‐G4KO undergo little apoptosis (Fig 3C). In fact, the propensity of these cells to survive is exacerbated so that they can easily be grown *ex vivo*, unlike FLB1 transplanted in WT animals. We also compared the translational activity of FLB1‐G4WT and FLB1‐G4KO *in vivo* in WT and synt‐KO animals, respectively, using O‐propargyl‐puromycin labeling methods (Fig 3D; Signer *et al*, 2014). AML blasts evolving in synt‐KO mice showed a significant twofold increase in total protein synthesis (Fig 3D; Appendix Fig S2D). Total protein synthesis in the nonmalignant (CD45.1^−^) host cells, in contrast, was similar in WT and synt‐KO mice (Fig 3C; Appendix Fig S2D). Assessing classical pathways associated with cell survival and protein translation, we found that FLB1‐G4KO cells display higher cellular levels of total AKT, associated with increased levels of AKT phosphorylated on S473 and T450 (Fig 3E). In contrast, FLB1‐G4KO show no activation of STAT3, suggesting specific activation of survival pathways (Appendix Fig S2E). We additionally tested for the activation of RPS6, a downstream effector of AKT involved in protein synthesis. Interestingly, FLB1‐G4KO cells showed a significant increase in the cellular level of RPS6, as well as increased levels of phosphorylated S235‐RPS6 (Fig 3D).

In an attempt to identify a “molecular node” associated with the activation of these pathways, we carefully analyzed the proteins differentially expressed in FLB1‐G4KO cells. Interestingly, we noted the eukaryotic translation elongation factor 1 alpha 2 (EEF1A2) ranked among the top upregulated factors (Fig 3A). EEF1A2 is a translation factor implicated in the delivery of aminoacyl‐tRNAs to the A site of the ribosome (Abbas *et al*, 2015). It is also known as a proto‐oncogene, whose expression is linked to anti‐apoptotic functions in several types of cancer, including AML, through the activation of AKT/mTORC1/RPS6 signaling (Li *et al*, 2010; Pellegrino *et al*, 2014; Sun *et al*, 2014). Western blot confirmed that EEF1A2 was significantly upregulated, nearly fivefold (Fig 3F). Then, we investigated to what extent gain of EEF1A2 can explain the gain of AML survival and aggressiveness. When treating aggressive FLB1‐G4KO cells, *ex vivo*, with increasing concentrations of metarrestin, an EEF1A2 inhibitor (Frankowski *et al*, 2018), we observed reduced cellular levels of pS473‐AKT, pT450‐AKT, RPS6, and pS235‐RPS6 (Appendix Fig S3A). Metarrestin treatment was also associated with a dose‐dependent apoptosis of aggressive FLB1‐G4KO cells *ex vivo* (Appendix Fig S3B). The high level of apoptosis of FLB1‐G4WT cells, very difficult to maintain *ex vivo*, was not significantly affected by a 24 h treatment with 10 μM of metarrestin (Appendix Fig S3C). As mice did well tolerate Metarrestin treatment for up to 14 days (Appendix Fig S3D–F), we also tested whether such treatment would impact bone marrow invasion by FLB1‐G4KO and FLB1‐G4WT. While we observed no effect of the treatment on FLB1‐G4WT, Metarrestin had a significant effect on bone marrow invasion by FLB1‐G4KO (Appendix Fig S3F).

Altogether, our data reveal that a syntenin‐deficient environment may ultimately lead to the activation of EEF1A2/AKT/RPS6 molecular pathways in AML cells, stimulating protein synthesis and enhancing cell survival, advantages educating the leukemia to become more aggressive.

### *Ex vivo* long‐term co‐culture with syntenin‐deficient BMSC suffices to convey an AML aggressive phenotype

**Figure 4:**
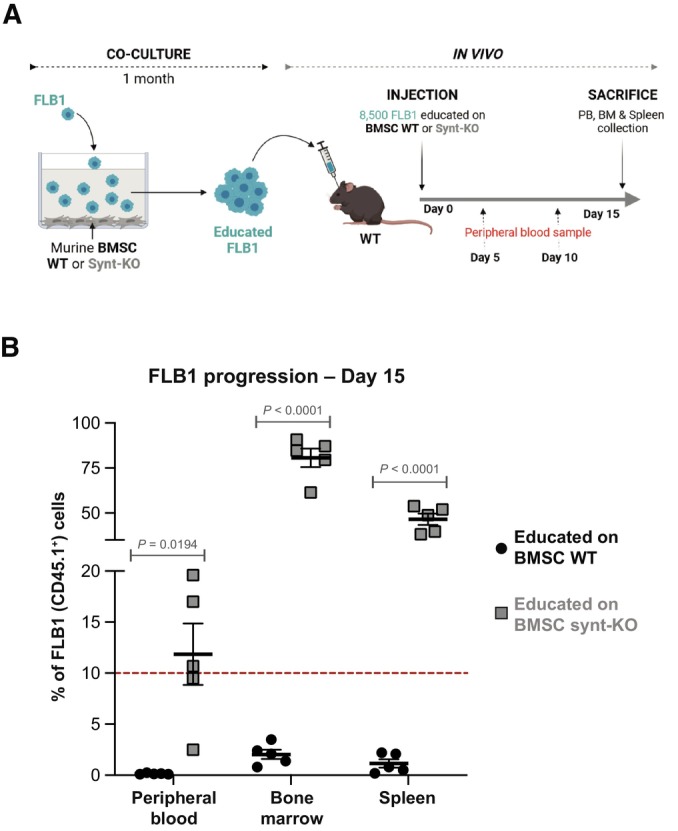
Syntenin‐deficient BMSC suffice to educate AML cells for aggressiveness Scheme for *ex vivo* long‐term FLB1‐BMSC co‐culture experiments coupled to *in vivo* leukemia burden assays. FLB1 cells were co‐cultured with murine BMSC isolated from WT or synt‐KO mice. After 1 month of co‐culture, surviving FLB1 cells, “educated” by murine BMSC (WT or synt‐KO), were injected into the retro‐orbital vein of WT mice. Leukemia progression was assessed weekly by FACS analysis as described before.Results of FACS analyses showing FLB1 (CD45.1^+^) cell frequencies measured in PB, BM, and the spleen of the different mice in the different groups 15 days after cell inoculation. Results are expressed as percentage of FLB1 (CD45.1^+^) cells relative to total CD45^+^ cells, and calculated as means ± SEM. Statistical analysis was performed using one‐way ANOVA test. Source data are available online for this figure.

BMSC were previously reported to support AML aggressiveness by diverse mechanisms (Ayala *et al*, 2009; Brenner *et al*, 2017; Corradi *et al*, 2018). We, therefore, aimed to clarify whether syntenin‐KO BMSC might account for the AML aggressive phenotype. No conditional knockout targeting BMSC is available as this is a highly heterogenous population. We therefore isolated BMSC from both WT and synt‐KO mice and expanded these *in vitro* (Appendix Fig S4A). BMSC from both sources appear similar in terms of differentiation potential (Appendix Fig S4B). To test whether synt‐KO BMSC would be competent to educate AML *in vitro*, we kept FLB1 cells in culture, in the presence of BMSC, WT or synt‐KO, for 1 month (Fig 4A). Of note, original FLB1 cells were difficult to maintain *ex vivo*, in particular when co‐cultured with BMSC WT, and considerable amounts of cells were required to conduct these experiments. After 1 month of co‐culture, the remaining FLB1 (CD45.1^+^) cells were collected and injected into WT animals. At the moment when the disease just became detectable in animals injected with FLB1 maintained on a WT stroma, we noticed an already important invasion of the peripheral blood, the bone marrow, and the spleen when FLB1 had been co‐cultured with synt‐KO BMSC (Fig 4B). This result demonstrates that exposure to synt‐KO stromal cells alone suffices to educate AML cells to become more aggressive.

### Syntenin‐deficient BMSC display altered subcellular endoglin expression

**Figure 5:**
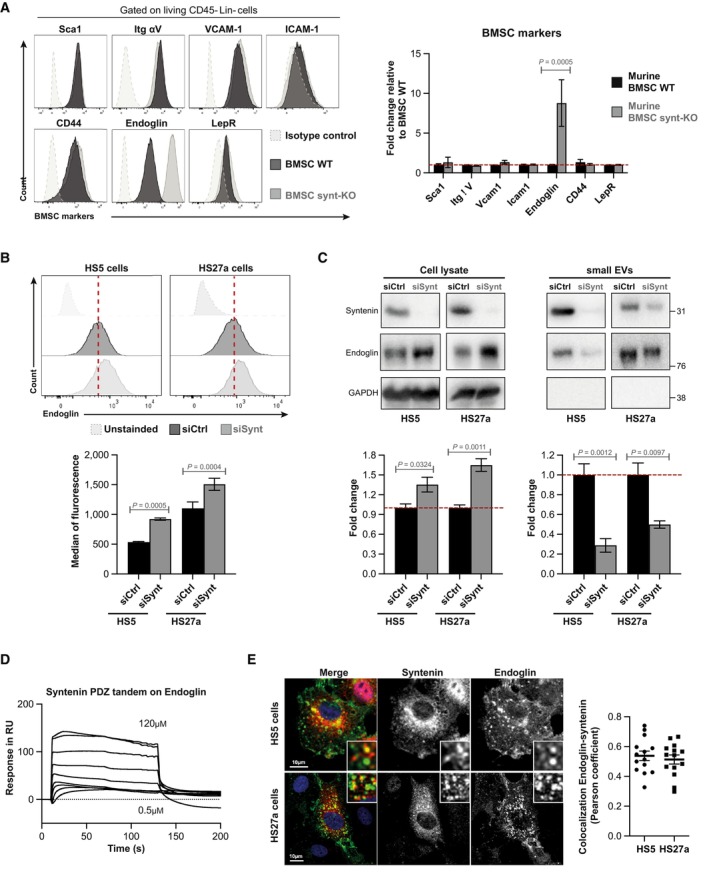
Syntenin deficiency in BMSC affects the subcellular expression of endoglin Left, FACS analysis of cell surface BMSC markers (as indicated) in WT (black lane) or Synt‐KO (gray lane) murine expanded BMSC (passage 4). Right, histogram representing the fold change in the median of fluorescence relative to that measured in BMSC WT ± SEM, calculated from the analysis of five independent experiments. Statistical analysis was performed using parametric Student's *t*‐test.Upper, FACS detection of the cell surface expression of endoglin in HS5 and HS27a stromal cells transfected with siRNA‐targeting syntenin (siSynt; gray histogram) or control siRNA (siCtrl; black histogram). Lower, histogram representing the median of fluorescence of endoglin ± SEM. Values were calculated from three independent experiments. Statistical analysis was performed using the two‐way analysis of variance (ANOVA).HS5 and HS27a cells were transfected with siRNA‐targeting syntenin (siSynt) or control siRNA (siCtrl). After 48 h, the media were changed and the transfected cells were cultured in medium containing EV‐depleted FCS (10%) for 16 h. Conditioned medium was submitted to differential centrifugation and small EVs were pelleted at 100,000 *g*. Upper, total cell lysates and corresponding pelleted extracellular particles (small EVs) were analyzed for indicated markers. Lower, histograms represent the endoglin protein levels in HS5 and HS27a cell lysates (normalized to GAPDH) and in corresponding small EVs. Values were calculated from three independent experiments. Statistical analysis was performed using one‐way ANOVA test.Surface plasmon resonance experiment illustrating the direct syntenin–endoglin interaction, tested with recombinant polypeptides. Increasing concentrations of syntenin (comprising only the tandem‐PDZ domains + C‐terminal domain; 0.5 μM to 120 μM) were perfused on an immobilized peptide corresponding to the last 25 C‐terminal amino acids of wild‐type endoglin.Left, representative confocal micrographs showing the steady‐state distributions of endogenous syntenin and endogenous endoglin in HS5 and HS27a stromal cells. In merge, nuclei are stained with DAPI (blue), syntenin is in red, and endoglin is in green. Right, Pearson correlation coefficient in 16 HS5 and 14 HS27a cells using the JACoP plugin on ImageJ. Dot plots with bars representing the mean Pearson coefficient ± SEM from three independent experiments. Source data are available online for this figure.

**Figure EV1:**
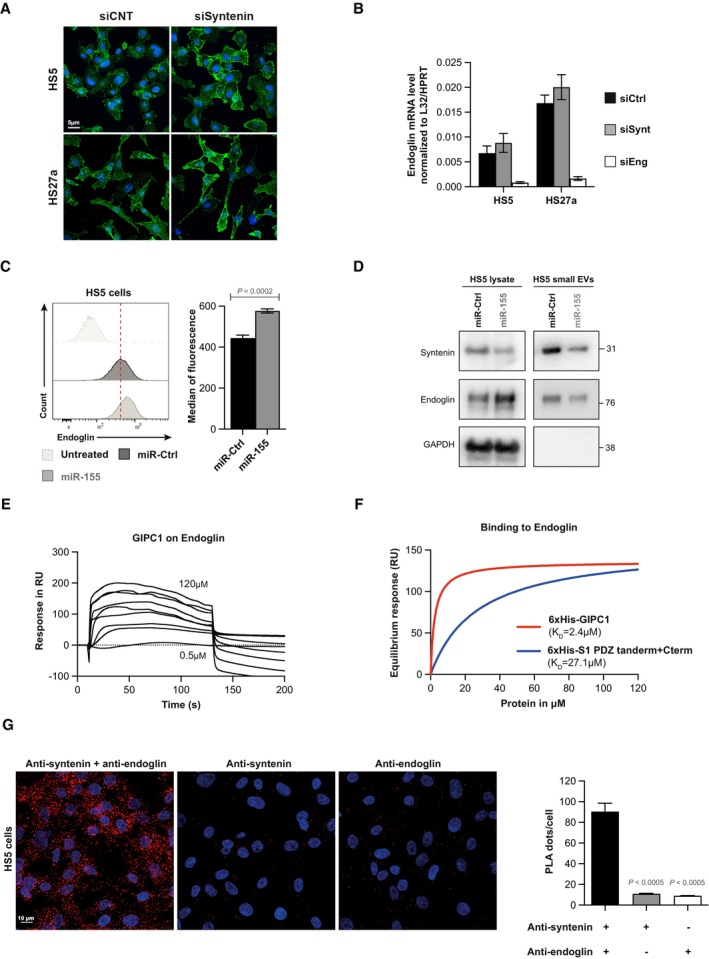
(Related to Fig 5). Syntenin–endoglin relationship Effect of loss of syntenin on endoglin expression (A–D) and endoglin–syntenin interaction assays (E–G).
Representative confocal micrographs showing the subcellular distribution of endogenous endoglin in HS5 and HS27a cells, depleted for syntenin expression (siSynt) or treated with control siRNA (siCtrl). Nuclei are stained with DAPI (blue), and endoglin is in green.Real‐time PCR analysis of endoglin mRNA expression in HS5 and HS27a cells depleted for syntenin (siSynt) and endoglin (siEng) or treated with control siRNA (siCtrl). Values were normalized to housekeeping L32/GAPDH genes. Data represent the mean ± SEM of four independent experiments performed in duplicate.Left, FACS analysis of endoglin levels in HS5, transfected with miR‐155‐5p (miR‐155) or control (miR‐Ctrl). Cells were immunostained with the anti‐human endoglin monoclonal antibody or isotype control antibody (untreated). Right, data represent the mean of the means of fluorescence intensity ± SEM, collected from three independent experiments. Statistical analysis was performed using the nonparametric Mann–Whitney *U*‐test.HS5 cells were transfected with miR‐155‐5p (miR‐155) or control (miR‐Ctrl). After 48 h, the media were changed and the transfected HS5 cells were cultured in medium containing EV‐depleted FCS (10%) for 16 h. Conditioned medium was submitted to differential centrifugation and small EVs were pelleted at 100,000 *g*. Total cell lysates and corresponding pelleted extracellular particles (small EVs) were analyzed for indicated markers.Surface plasmon resonance experiment illustrating the direct interaction of endoglin with GIPC1, used as positive control. Sensorgrams illustrating the binding of GIPC1, at increasing concentrations of recombinant GIPC1 (0.5 μM to 120 μM), to an immobilized peptide corresponding to the last 25 C‐terminal amino acids of wild‐type endoglin.Langmuir graph showing the binding (in resonance units, RU) of recombinant syntenin (blue curve) and GIPC1 (red curve) as observed at equilibrium, at increasing concentrations of analyte. KD (apparent) was calculated from the protein concentration required to observe half of the maximal response.Proximity ligation assay (PLA) detecting the proximity of endogenous syntenin and endoglin in HS5 cells. Left, representative images of confocal microscopy showing the PLA signals. Nuclei were stained with DAPI. Syntenin and endoglin antibodies were incubated alone or together. Each picture is representative of a typical cell staining pattern observed in five fields chosen at random. Right, the quantification of the number of PLA dots per nucleus is represented as the mean values ± SEM from a single experiment performed in duplicate. Statistical analysis was performed using the one‐way analysis of variance (ANOVA).

To clarify the pro‐tumoral mechanisms at work in syntenin‐deficient stroma, we compared the mesenchymal patterns of WT and synt‐KO murine BMSC. To our surprise, we noticed markedly increased levels of endoglin protein at the surface of *in vitro* expanded synt‐KO BMSC, compared to WT (Fig 5A). A similar phenomenon was observed in human BMSC, using the stromal cell lines HS5 and HS27a as models. Consistent with the observation in murine BMSC, syntenin deficiency in HS5 and HS27a cells is also accompanied by an increase in endoglin protein at the cell surface (Figs 5B and EV1A) and of total cellular endoglin (Fig 5C). We found that syntenin deficiency does not impact endoglin gene transcription (Fig EV1B) nor protein stability (Appendix Fig S5A and B). Because of the important role of syntenin in exosomal releases, we tested for a potential defect of vesicular endoglin secretion. Comparing small extracellular vesicles (small EVs) purified by differential ultracentrifugation (enriched in exosomes), we found a significant decrease in endoglin levels in the small EVs isolated from both HS5 and HS27a cells with syntenin deficiency (Fig 5C). Interestingly, treatment of HS5 with miR‐155 mimics the effects of syntenin deficiency, increasing the levels of cell surface and total cellular endoglin (Fig EV1C and D) and decreasing endoglin levels associated with extracellular vesicles (Fig EV1D). Endoglin is a transmembrane glycoprotein modulating TGFβ signaling, notably through its PDZ interactions (Lee *et al*, 2008). As PDZ interactions are known to be promiscuous (Ivarsson, 2012) and as syntenin is a PDZ protein, we investigated whether syntenin might directly bind endoglin. By surface plasmon resonance (SPR) experiments, we found that the PDZ domains of syntenin interact directly with endoglin cytosolic domain, in a concentration‐dependent manner (Fig 5D), similar to GIPC1, a previously described endoglin PDZ interactor (Fig EV1E). The apparent K_D_ values were determined to be 27.1 and 2.4 μM, respectively (Fig EV1F). In addition, confocal imaging revealed that endogenous endoglin partially colocalizes with endogenous syntenin, at intracellular sites in both HS5 and HS27a models (Fig 5E). Colocalization in the cytoplasm of HS5 cell was confirmed by *in situ* proximity ligation assay (PLA; Fig EV1G). Altogether, these data show that syntenin controls the distribution of endoglin in stromal cells.

### High endoglin expression in BMSC supports AML phenotypic changes

**Figure 6:**
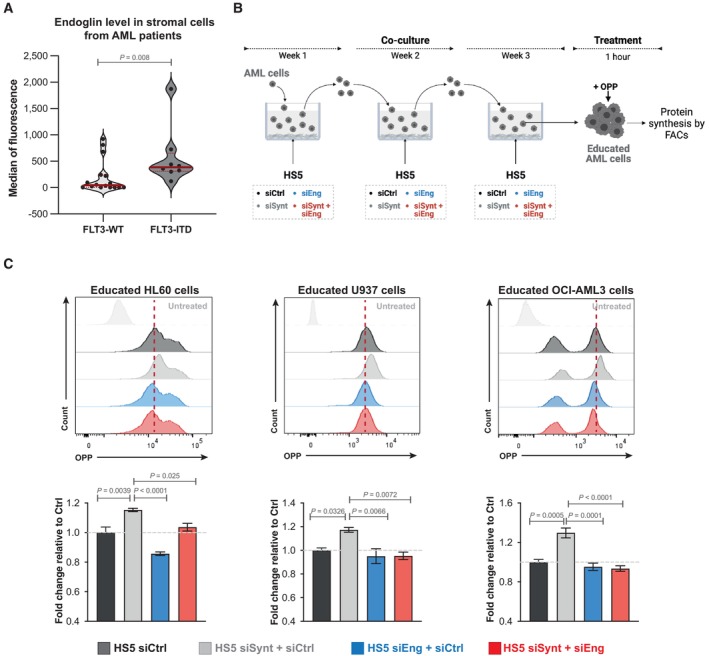
High endoglin cell surface expression in BMSC promotes AML aggressiveness FACS analysis of cell surface expression of endoglin in stromal cells from AML patients with wild‐type FLT3 (WT) or bearing FLT3‐ITD mutation. As indicated in Dataset EV4, endoglin expression was assessed in samples where at least 300 stromal cells were reliably detected. The violin plots represent the median fluorescence of endoglin expression at the surface of the stromal cells minus the value of the isotypic control. Each dot represents one patient. Statistical analysis was performed using the nonparametric Mann–Whitney *U*‐test.Scheme illustrating long‐term co‐culture experiments with syntenin‐ and endoglin‐deficient stromal cells addressing stromal effects on AML protein synthesis. HS5 cells were transfected with siRNA‐targeting syntenin (siSynt), endoglin (siEng), or control siRNA (siCtrl). Twenty‐four hours later, human AML HL60, U937, or OCI‐AML3 cells were co‐cultured with these HS5 cells. After 1 week of co‐culture, AML cells were collected and re‐seeded on freshly transfected HS5 cells. After 3 weeks of co‐culture, AML cells were collected and treated with O‐propargyl‐puromycin (OPP) to measure protein synthesis by FACS.Upper, FACS detection of OPP incorporation in AML cells educated on HS5 treated as indicated. Untreated refers to AML cells not treated with OPP. Lower, graph representing the relative levels of protein synthesis in AML cells (HL60, U937, or OCI‐AML3) normalized to untreated AML cells ± SEM. Values were calculated from four independent experiments. Statistical analysis was performed using the one‐way analysis of variance (ANOVA). Source data are available online for this figure.

**Figure EV2:**
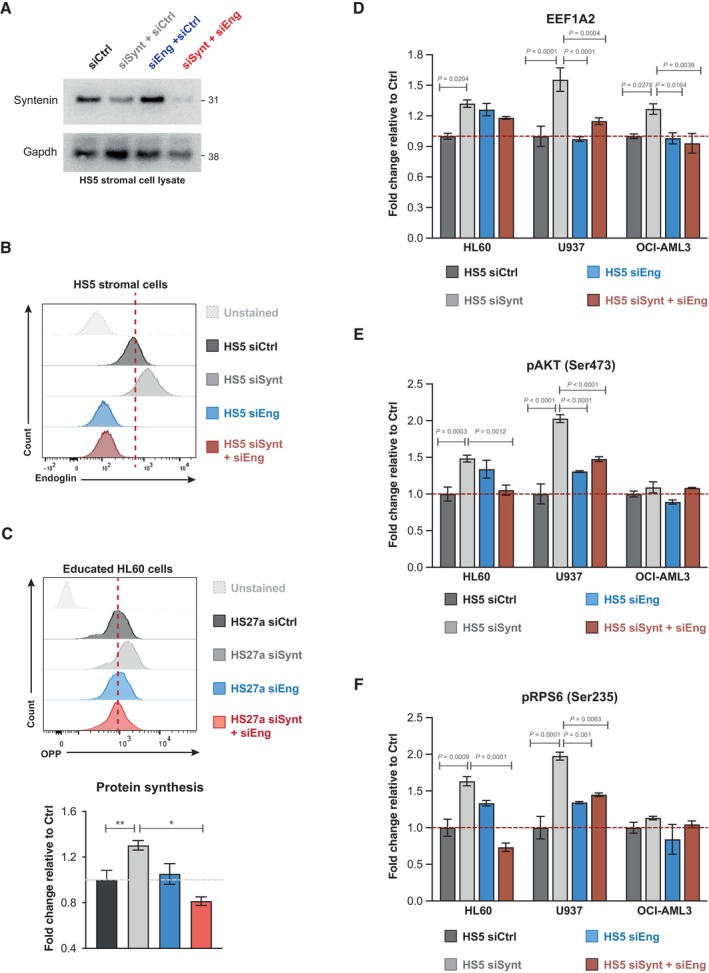
(Related to Fig 6). Endoglin/syntenin suppression in HS5 cells (control transfection experiments) AWestern blots of the lysates of HS5 cells transfected with control siRNA (siCtrl), with control siRNA and siRNA‐targeting syntenin (siSynt + siCtrl) or endoglin (siEng + siCtrl), or siRNA‐targeting syntenin and endoglin (siSynt + siEng; 30 nM), showing the effects on syntenin levels. GAPDH was used as control.BFACS analysis of the endoglin expression at the surface of HS5 cells transfected with control siRNA (siCtrl, dark gray), with control siRNA and siRNA‐targeting syntenin (siSynt + siCtrl, light gray) or endoglin (siEng + siCtrl, blue), or siRNA‐targeting syntenin and endoglin (siSynt + siEng; red). Isotype control antibody was used as control (unstained).CHS27a (stroma) cells were transfected with siRNA‐targeting syntenin (siSynt), endoglin (siEng), both (siSynt + siEng), or control siRNA (siCtrl). After 24 h, HL60 (leukemia) cells were put in contact with the transfected HS27a cells. After 1 week of co‐culture, HL60 cells were collected and re‐seeded on freshly transfected HS27a cells. After 3 weeks of co‐culture, AML cells were collected and treated with O‐propargyl‐puromycin (OPP) to measure protein synthesis by FACS. Upper panel, FACS detection of OPP incorporation in HL60 cells educated on HS27a treated as indicated. Untreated refers to HL60 cells not treated with OPP. Lower panel, graph representing the relative levels of protein synthesis in HL60 cells, normalized to untreated HL60 cells ± SEM. Values were calculated from four independent experiments. Statistical analysis was performed using the one‐way analysis of variance (ANOVA).D–FHS5 cells were transfected with siRNA‐targeting syntenin (siSynt), endoglin (siEng), both (siSynt + siEng), or control siRNA (siCtrl). Twenty‐four hours later, human AML HL60, U937, or OCI‐AML3 cells were co‐cultured with these HS5 cells. After 1 week of co‐culture, AML cells were collected and re‐seeded on freshly transfected HS5 cells. After 3 weeks of co‐culture, AML cells were collected for FACS analysis of (D) EEF1A2, (E) pAKT (Ser473), and (F) pRPS6 (Ser235), respectively. The histograms represent the fold change in mean of fluorescence intensity relative to the signal in AML cells maintained on HS5 cells transfected with siCtrl ± SEM calculated from the analysis of three independent experiments. Statistical analysis was performed using the nonparametric two‐way analysis of variance (ANOVA).

Finally, we assessed whether endoglin may be implicated in the acquisition of an aggressive AML phenotype conveyed by a syntenin‐deficient stroma. First, in patients, we observed that aggressive AML forms carrying the FLT3‐ITD mutation display significantly higher endoglin expression on the surface of stromal cells, compared to FLT3 wild‐type AML (Fig 6A; Dataset EV4). Then, we evaluated the effect of a syntenin^low^/endoglin^high^ stroma on AML. For that, we made use of human stromal cell lines (HS5 and HS27a) and several AML cell lines, including HL60 (mutated for Nras and Cdkn2a), U937 (mutated for Pten and p53), and OCI‐AML3 (mutated for Nras and Dnmt3a). HS5 and HS27a stromal cells were silenced for syntenin, endoglin, or both, and co‐cultured with human AML cells for 1 month (Fig 6B). To maintain the level of stromal syntenin or stromal endoglin at their minimum during the whole experiment, stroma‐exposed AML cells were collected each week and re‐seeded on freshly transfected (silenced) HS5 or HS27a cells (Fig EV2A and B). In agreement with our *in vivo* observations on FLB1 cells, co‐culture with syntenin‐deficient HS5 or HS27a stromal cells significantly stimulates *in trans* the protein synthesis of AML cells (siCtrl vs. siSynt; Figs 6C and EV2C). Such co‐culture is associated with an increase in EEF1A2 levels in all AML cells (siCtrl vs. siSynt; Fig EV2D). Syntenin deficiency also causes an increased phosphorylation of S473‐AKT and S235‐RPS6 (siCtrl vs. siSynt; Fig EV2E and F) in HL60 and U937 AML cells. Yet, this effect was not observed in OCI‐AML3 cells (Fig EV2E and F), suggesting cell type‐dependent effects. Interestingly, when silencing endoglin or both syntenin/endoglin in HS5 stromal cells, the level of protein synthesis in AML cell lines remains at a “baseline level,” similar to the level observed in AML cells co‐cultured with HS5 control cells (Fig 6C). A similar result is observed when HS27a stromal cells are silenced for both syntenin/endoglin, with a “return” of the protein synthesis to a baseline level in HL60 cells (Fig EV2C). Moreover, we noticed that the co‐silencing of endoglin (in stromal cells treated with siSynt) reduces the level of EEF1A2 in AML, markedly in U937 and OCI‐AML3, but not significantly in HL60 cells (Fig EV2D). Similarly, HL60 and U937 cells educated on a stroma deficient for both syntenin/endoglin showed lower phosphorylation of S473‐AKT and S235‐RPS6 than when educated on a syntenin‐deficient stroma. Of note, the inhibition of endoglin, syntenin, or both syntenin/endoglin expression in HS5 cells does not appear to affect S473‐AKT and S235‐RPS6 phosphorylation in OCI‐AML3 cells (Fig EV2E and F). Thus, specific pathways activated downstream of the upregulated EEF1A2 may diverge between different types of AML. Yet, altogether, these results suggest that an “endoglin high” stroma may be implicated in the acquisition of the aggressive AML phenotype promoted by a syntenin‐deficient tumor environment.

## Discussion

**Figure 7:**
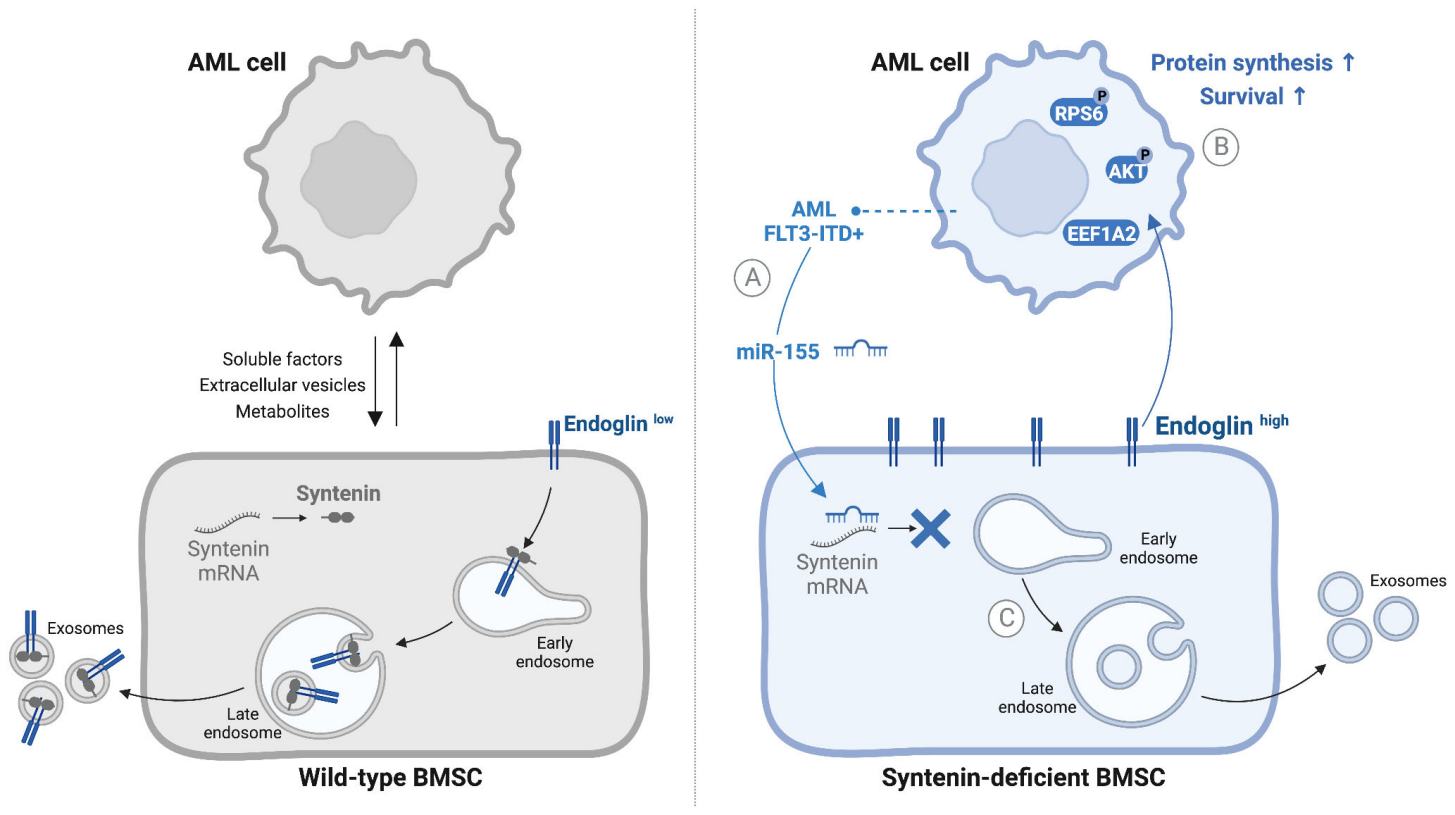
Model recapitulating the findings presented in this study We here summarize cues that might provide novel insight into the mechanisms at work in tumor–stroma crosstalk.
AAggressive AML (FLT3‐ITD) cells overexpressing miR‐155 can suppress syntenin expression in stromal cells.BAML blasts confronted with a syntenin‐deficient stroma acquire a cell survival advantage associated with increased levels of protein synthesis. This AML response relies on the upregulation of EEF1A2, an elongation factor and notable activator of the AKT/RPS6 signaling pathway.CSyntenin loss/dysregulation increases the level of endoglin at stromal cell surfaces, while decreasing the loading of endoglin into exosomes. Stromal endoglin, and likely high endoglin at stromal cell surfaces, is needed for the *in trans* support of AML translational activity by a syntenin‐deficient stroma.

Using an AML‐relevant syngeneic mouse model and *in vitro* human and mouse co‐culture assays, we here reveal several unsuspected molecular mechanisms that may govern tumor–stroma communication. Indeed, our study indicates that downregulation of stromal syntenin might be at the heart of a pro‐tumoral vicious signaling loop between the tumor cells and the associated bone marrow stromal cells (Fig 7).

In the context of AML, we found that such signaling loop might be initiated by “aggressive” blasts, in particular AML of the FLT3‐ITD^+^ subtype overexpressing miR‐155. Indeed, AML that are miR‐155 high are able to downregulate stromal syntenin in co‐culture (Fig 1E). A phenomenon that is not observed with AML expressing low levels of miR‐155. This is intriguing because the pro‐tumoral effect of miR‐155 is currently primarily considered to take place via mechanisms occurring in the tumor cells. This holds for AML blasts, and also for glioma, lung, colorectal, or breast cancers (Bayraktar & Van Roosbroeck, 2018). We hereby provide evidence that the pro‐tumoral effect of miR‐155 may also depend on effects that occur *in trans*, that is, via downregulation of stromal syntenin (Fig 1E). The publicly available data on *in vivo* expressions indicate that other AML subtypes bearing different genetic alterations, in particular MLL‐ENL, are capable of reducing syntenin expression in BMSC (Fig 1B). This suggests that other factors might be involved in the downregulation of stromal syntenin expression. miR‐7977 might be a relevant candidate since it can be transferred from different subtypes of AML cells to BMSC (Horiguchi *et al*, 2016) and syntenin is one of its main targets (Yoshida *et al*, 2019). Therefore, the regulation of stromal syntenin expression in distinct AML subtypes requires deeper analysis.

The finding that leukemic blasts confronted with a syntenin‐KO environment gain in aggressiveness was completely unexpected (Fig 2B). Indeed, up to now, syntenin was unambiguously considered a valuable target for cancer therapy, and several inhibitors have already been developed and tested in preclinical models (Kegelman *et al*, 2017; Leblanc *et al*, 2020; Pradhan *et al*, 2020, 2021). This is because gain of syntenin expression in tumor cells has been invariably associated with the invasion and the metastatic potential of various solid tumors, including melanoma, glioblastoma, breast, prostate, and head/neck squamous cancers (Pradhan *et al*, 2020). Moreover, syntenin‐KO mice were previously shown to be refractory to melanoma metastasis due to reduced tumor‐supporting inflammation (Das *et al*, 2016). One reason for this apparent controversy may be that the B16 melanoma tumor model used in these (single graft) experiments was only shortly exposed to the syntenin‐deficient microenvironment, while our study addressed the effects of long‐term exposure to a syntenin‐deficient stroma (Figs 2B and 4B). Another trivial explanation might be that a syntenin‐deficient stroma has different consequences depending on the tumor type. That remains to be clarified; our study invites to also considerate the long‐term effects of anti‐syntenin therapy in the context of cancer treatment. It implies that the suitability and efficacy of syntenin as a systemic target in cancer therapy (including the emergence of drug resistance) may ultimately also depend on monitoring and compensating for environmental loss of syntenin activity.

Remarkably, the spectacular gain of survival acquired by AML blasts facing a syntenin‐KO environment (Fig 2B) is associated with a twofold increase in tumor translational activity (Fig 3D). Similar results are observed *in vitro*, in several different human AML models held in long‐term co‐culture with human BMSC (Figs 6C and EV2C). In the last decade, aberrant translation emerged as an important player in the pathogenesis of cancers (Song *et al*, 2021). The precise molecular mechanism by which stroma supports high protein synthesis in the tumor remaining enigmatic, we here show that stromal syntenin deficiency induces a fivefold increase in the elongation factor EEF1A2 (Figs 3A and F, and 7B) in AML. This was confirmed *in vitro*, in different AML models co‐cultured with syntenin‐deficient BMSC (Fig EV2D). In addition to its role in aminoacyl‐tRNA delivery to the ribosome, EEF1A2 is known for its pro‐oncogenic activity in many tumors, enhancing cancer cell proliferation and inhibiting apoptosis (Abbas *et al*, 2015). Moreover, EEF1A2 is known to activate the pro‐survival AKT signaling pathway (Chang & Wang, 2007; Sun *et al*, 2014; Abbas *et al*, 2015). This seems consistent with our observations that AML blasts educated in a syntenin‐deficient environment display activated AKT/RPS6 signaling and survive better (Figs 3E and EV2E and F). Yet, there are some discrepancies *in vitro* depending on the AML models used (Fig EV2E and F), suggesting the activation of pathways other than AKT/RPS6 signaling. Overall, however, it is tempting to propose that EEF1A2 works as a node supporting the pro‐tumoral effects elicited by the stromal deficiency of syntenin. In agreement with this contention, we observed that pharmacological inhibition of EEF1A2 by Metarrestin, *in vitro*, drastically induces apoptosis of AML blasts educated by a syntenin‐KO stroma (Appendix Fig S3B). Moreover, Metarrestin treatment significantly reduces the aggressiveness of blasts educated in a syntenin‐KO host but has no effect on AML blasts grown in wild‐type mice (Appendix Fig S3F).

In search of mechanisms supporting the stromal syntenin‐KO pro‐tumoral effects, we found that the TGFβ co‐receptor endoglin is highly expressed at cell membranes in syntenin‐deficient BMSC (Fig 5A and B). Evidence emerges that high endoglin expression in stromal cells (BMSC or cancer‐associated fibroblasts) supports glioma, and breast and pancreatic cancer progression (Hutton *et al*, 2021; Muñoz *et al*, 2021). In agreement with such observations, our preliminary results with AML patients strongly suggest that endoglin expression at the cell surface of BMSC is upregulated in aggressive AML bearing FLT3‐ITD mutation. So far, the pro‐tumoral modes of action of stromal endoglin are unknown. We here established, in the context of AML, that high‐stromal endoglin is required to support *in trans* the gain of tumor translational activity induced by stromal syntenin deficiency (Figs 6C, EV2C and 7C). Interestingly, the increase in endoglin protein levels is not correlated with an increase in endoglin RNA levels in stromal cells (Fig EV1B). Thus, the mechanism by which syntenin deficiency leads to higher cellular/membrane levels of endoglin might be related to role of syntenin in late endosomal trafficking. Indeed, syntenin is well‐known to address its PDZ interactors to the lumen of late endosomes for exosomal secretion (Zimmermann *et al*, 2005; Baietti *et al*, 2012). Note that we here established that endoglin binds to the syntenin PDZ domains and that the two proteins partly colocalize intracellularly (see Figs 5D and E, and EV1G). The increased cellular levels of endoglin observed upon syntenin loss of function might thus (at least in part) be due to defective targeting of endoglin to late endosomes/exosomal secretion. The much lower levels of endoglin accumulating into small EVs isolated from syntenin‐deficient stromal cells (Fig 5C) strongly supports this hypothesis. Secreted endoglin has recently been shown to exert anti‐tumoral effect by working as decoy in TGFβ signaling (Castonguay *et al*, 2011). Whether a lack of vesicular endoglin secretion may contribute to the pro‐tumoral effect of syntenin‐deficient stroma remains to be clarified. Additionally, or alternatively, as co‐receptor for TGFβ, cell‐associated endoglin will potentially support intracellular SMAD and AKT signaling (Muñoz *et al*, 2021). Noteworthy, the TGFβ receptor ligand BMP9 was recently shown to stimulate protein synthesis by inducing the activation of RPS6 (Medina‐Jover *et al*, 2020). It would thus not be surprising that stromal endoglin and tumor TGFβ receptor cooperate *in trans* to potentiate AKT/RPS6 signaling and protein synthesis in AML cells.

Overall, our study underscores the importance of syntenin for cell‐to‐cell communication and uncovers unexpected molecular mechanisms that may govern tumor–stroma communication (Fig 7).

## Materials and Methods

Antibodies and reagents are listed in Appendix Table S1.

### Cell lines

All human cell lines were obtained from the American Type culture collection (ATCC; Manassas, USA) and authenticated (STR profiling) by Eurofins Scientific SE (Luxembourg). They were grown in media supplemented with 10% heat‐inactivated FBS (Eurobio, Les Ulis, France) and incubated at 37°C, with 5% CO_2_. Human bone marrow stromal cell lines HS5 and HS27a were maintained in DMEM (Gibco, Carlsbad, USA). AML cell lines U937 (FLT3‐negative, miR‐155^low^) and HL60 (FLT3‐WT, miR‐155^low^; Gerloff *et al*, 2015; Salemi *et al*, 2015) were maintained in minimum essential medium‐α medium (Gibco, Carlsbad, USA). MOLM‐14 (FLT3‐ITD^+^, miR‐155^high^; Wallace *et al*, 2017) and OCI‐AML3 (FLT3‐WT) were maintained in RPMI‐1640 medium (Gibco, Carlsbad, USA). The cultured cells were split every 2–3 days, maintaining an exponential growth. All cell lines were routinely tested for mycoplasma contamination (Mycoplasmacheck—qPCR‐based service for detection of mycoplasma, Eurofins Scientific SE, Luxembourg). FLB1 (miR‐155^low^) primary mouse leukemia cells were provided by Pr. O. Hérault and were generated into C57BL/6J CD45.1 mice, as described previously (Wilhelm *et al*, 2011). Patient samples were obtained from the IPC/CRCM Tumor Bank/Biological Resource Center for Oncology that operates under authorization # AC‐2018‐1905 granted by the French Ministry of Research (“Ministère de l'Enseignement Supérieur, de l'Innovation et de la Recherche”). Patient samples were stored at IPC/CRCM Tumor Bank and obtained after informed consent from all subjects and that the experiments conformed to the principles set out in the WMA Declaration of Helsinki and the Department of Health and Human Services Belmont Report.

### Cell transfection

For RNAi experiments, cells at a confluence of 50% were transfected with 30 nM RNAi using Lipofectamine RNAiMAX reagent (Life Technologies, USA); cells were analyzed 48–96 h after RNAi treatment. RNAi targeting human SDCBP (5′‐GCAAGACCUUCCAGUAUAA‐3′), a SMART pool targeting human ENG, and the nontargeting control RNAi (siCNT) were purchased from Dharmacon Inc. For miRNA treatment, HS5 cells were transiently transfected using 30 nM of hsa‐miR‐155‐5p mimic or 50 nM of mirVana negative control, in the presence or absence of 30 nM of antagomiR‐155 (miR‐155 inhibitor). Expanded murine BMSC WT were transiently transfected using 30 nM of mmu‐miR‐155‐5p mimic or 50 nM of mirVana negative control.

### Animal studies

CD45.2 C57BL/6J mice were purchased from Janvier Laboratories (France). CD45.2 C57BL/6J syntenin‐knockout (synt‐KO) animals were generated as previously described (Kashyap *et al*, 2021) and the hygromycin resistance gene was removed using Flipper mice (Farley *et al*, 2000). Both male and female mice were used between 6 and 11 weeks of age and were housed under specific pathogen‐free conditions. All experiments were performed in compliance with the laws and protocols approved by animal ethics committees (Agreement No. APAFIS#5123‐2016041809182209v2). For *in vivo* expansion, freshly thawed 50,000 CD45.1 FLB1 cells were resuspended in PBS and injected into the retro‐orbital vein of CD45.2 mice. Control mice consisted of PBS‐injected mice. AML development was monitored by flow cytometry, analyzing the percentage of CD45.1‐positive cells in the blood. Recipient mice were sacrificed when blast levels reached > 10% of total white blood cells in the peripheral blood (survival threshold), and leukemic cells were collected from femurs and tibias. For serial transplantation assays, blasts collected from the bone marrow (> 90% blast invasion) of three different animals were pooled and used (50,000 cells/mouse) for re‐injection in the following transplantation round. AML progression was assessed weekly, and animals were sacrificed at survival threshold.

### *In vivo* homing assays

For homing assays, a total of 3 × 10^6^ FLB1 (CD45.1) cells were injected into the retro‐orbital vein of WT or synt‐KO CD45.2 C57Bl/6J mice. Sixteen hours after cell inoculation, animals were sacrificed, and bone marrow, spleen, and lymph nodes were collected for analysis. Numbers of FLB1 in the bone marrow and the spleen were evaluated by flow cytometry using CD45.1/CD45.2 antibodies and CountBright™ Absolute Counting Beads according to the manufacturer.

### *In vivo* limiting dilution

Extreme limiting dilution analysis (ELDA) comparing populations for enrichment in stem cell was performed as previously described (Huy & Smyth, 2009). Five doses (20, 200, 500, 1,000 and 2,000) of CD45.1 FLB1‐G4 cells, isolated from WT or synt‐KO animals at graft 4, were transplanted into CD45.2 C57Bl/6J mice. The cut‐off for engraftment was set at FLB1 (CD45.1) cells composing more than 1% of the total white blood cells in the peripheral blood, up to 12 weeks after cell inoculation. Leukemia initiating cell (LIC) frequencies were determined using the webtool interface “http://bioinf.wehi.edu.au/software/elda/”.

### Isolation and culture of murine bone marrow stromal cells

Six‐ to eight‐week‐old female CD45.2 C57BL/6J WT or synt‐KO mice were sacrificed, and hind limbs were collected and stripped of their skin and muscle manually. For isolation of the primary cells, the bone marrow from the femora and the tibia was flushed in RPMI buffer containing 1% FBS and 1% of penicillin–streptomycin (Gibco, Carlsbad, CA, USA). Red blood cells were removed using ACK lysis buffer (Gibco, Carlsbad, CA, USA) for 10 min at room temperature. Cells were then washed twice into RPMI medium and seeded in a rat tail collagen‐1 (5 μg/cm^2^; Gibco, Carlsbad, CA, USA) coated flask for cell culture. Murine BMSC were routinely grown in RPMI medium supplemented with 10% FBS, 10% horse serum (Gibco, Carlsbad, CA, USA), 1% penicillin–streptomycin, and 1% sodium pyruvate, in plastic flasks coated with collagen. The growing cells were characterized as murine bone marrow stromal cells (BMSC) by flow cytometry analysis: dead cells were excluded using DRAQ7 (Beckman Coulter, Brea, CA, USA) and cells were stained with antibodies for the positive (Sca1, CD51, CD106, CD105, CD54, CD44, and CD295) and the negative (CD45, CD3e, CD11c, B220, CD11b, CD19, and Ter119) selection of this lineage. Expanded BMSC WT/synt‐KO were used in the fourth to the tenth passages.

### *In vitro* differentiation of murine expanded BMSC

Osteoblastic differentiation was induced by culturing expanded BMSC, WT/synt‐KO, for 4 weeks with 50 μg/ml of l‐ascorbic acid 2‐phosphate, 10 mM glycerophosphate (Sigma‐Aldrich, Saint‐Louis, MO, USA), and 15% FBS in α‐MEM supplemented with penicillin–streptomycin. Adipocyte differentiation was induced with 1 μM dexamethasone, 10 μg/ml of insulin (Sigma‐Aldrich), and 10% FBS in α‐MEM with penicillin–streptomycin. All cultures were maintained with 5% CO_2_ in a water‐jacketed incubator at 37°C, and media were replaced every 2–3 days. To assess *in vitro* differentiation into mesenchymal lineages, cells were briefly washed with phosphate‐buffered saline solution (PBS1X; Gibco, Carlsbad, CA, USA) and fixed for 10 min at room temperature with 4% paraformaldehyde (Santa Cruz). For the detection of mineral calcium deposition, osteoblasts were stained with Alizarin Red solution (Sigma‐Aldrich) for 30 min and rinsed three times with distilled water. Adipocytes were stained with Oil Red O (Sigma‐Aldrich) as follows: cells were washed with 60% isopropanol and allowed to dry completely. Oil Red O working solution was prepared as a 6:4 dilution in distilled water of a 0.35 g/ml Oil Red O solution in isopropanol (Sigma‐Aldrich) and filtered 20 min later. Cells were incubated for 60 min with Oil red O working solution and rinsed four times using distilled water.

### Long‐term co‐culture experiments

Transfected HS5 and passages 4–10 murine BMSC (WT or synt‐KO) were irradiated at 30 Gy prior to co‐culture to stop their proliferation during the co‐culture assay. Irradiated murine BMSC (10,000) or HS5 cells (25,000) were seeded in 12‐well plates and were cultured in RPMI medium containing exosome‐depleted FCS (10%) for 24 h. Then, AML cells (ratio 5:1 to stromal cells) were added and maintained in long‐term co‐culture for 1 month, with a media change every 3–4 days.

### Exosomes and total cell lysates

For comparative analyses, exosome‐enriched fractions were collected from equivalent amounts of culture medium, conditioned by equivalent amounts of HS5 cells transfected with siRNA‐targeting syntenin (siSynt) or control siRNA (siCtrl). After 16 h, cell media were collected and small extracellular vesicles were isolated by three sequential centrifugation steps at 4°C: 10 min at 500 *g*, to remove cells and large debris; 30 min at 10,000 *g*, to remove large extracellular vesicles; and 1 h 30 min at 100,000 *g*, to pellet small extracellular vesicles (exosome‐enriched fraction), followed by one wash with 1,400 μl of PBS (100,000 *g*, 1 h) to remove soluble serum and secreted proteins. Small extracellular vesicle pellets were then re‐suspended in 100 μl of PBS. The lysates from corresponding cultures were cleared by centrifugation at 125 *g* for 5 min and then resuspended in lysis buffer (Tris–HCL pH 7.4 30 mM, NaCl 150 mM, 1% NP40 (IGEPAL), 1 μg/ml aprotinin, and 1 μg/ml leupeptin). Although only small variations were observed from sample to sample, exosomal amounts loaded for the western blot were normalized according to the number of parent cells from where exosomes were secreted.

### Western blots

The proteins were heat denaturated in Laemmli sample buffer, fractionated in 12.5 or 15% gels by SDS–PAGE, and electrotransferred to nitrocellulose membrane. Membranes were stained with Ponceau red and immunoblotted with the indicated primary antibodies (Appendix Table S1) and HRP‐conjugated secondary antibodies (Mouse or Rabbit, Thermofisher Scientific; 1/10,000). Signals were visualized using Amersham ECL Prime Western Blotting Detection Reagent (GE Healthcare).

### Mass spectrometry analysis and protein quantification

FLB1 CD45.1 cells were isolated and sorted by FACS after four rounds of serial transplantation from WT and synt‐KO mice, using five animals for each condition. Two million FLB1 CD45.1 cells per condition were lysed in RIPA buffer and analyzed by mass spectrometry as described below.

#### Sample preparation and labeling

Equal amounts of protein (i.e., around 100 μg) were aliquoted and adjusted to 100 μl using 100 mM TEAB. Proteins were reduced using TCEP and then alkylated using iodoacetamide as mentioned in the TMT mass tag labeling instructions (Thermo Scientific). Proteins were precipitated using six volumes of cold acetone at −20°C overnight. The pellet was then suspended in 100 mM TEAB and digested with 2.5 μg of Trypsin (Promega), overnight at 37°C. Peptides were labeled with TMT reagents as described in the TMT labeling instructions (Thermo Scientific). Briefly, samples were transferred to TMT reagents reconstituted using 40ul of anhydrous acetonitrile, and then incubated at room temperature for 1 h. Five biological replicates for both conditions, that is, PWT and PKO FLB1, were distributed in the 10 TMT channels. The reaction was quenched by adding 8 μl of 5% hydroxylamine and incubating for 15 min. Labeled peptides were mixed, and dried to remove organic solvent prior to clean‐up via Sep‐Pak (50 mg C18 SepPak; Waters). Labeled peptide mixtures were separated into 80 fractions via high‐pH reversed‐phase chromatography (Waters; Xbridge C18 analytical column) and concatenated into 13 fractions. Samples were dried and stored at −80°C prior to LC–MS analysis.

#### Mass spectrometry analysis

Samples were reconstituted in 4% acetonitrile/0.1% trifluoroacetic acid prior to analysis by liquid chromatography (LC)‐tandem mass spectrometry (MS/MS), using an Orbitrap Fusion Lumos Tribrid Mass Spectrometer (Thermo Fischer) online with an Ultimate 3000RSLCnano chromatography system (Dionex). Peptides were separated on a reverse‐phase LC EASY‐Spray C18 column from Dionex (PepMap RSLC C18, 50 cm × 75 μm I.D, 100 Å pore size, and 2 μm particle size) at 300 nl/min flow rate and 40°C and using a two steps linear gradient (4–20% acetonitrile/H_2_O; 0.1% formic acid for 180 min and 20‐45‐45% acetonitrile/H_2_O; 0.1% formic acid for 60 min). An EASY‐Spray nanosource was used for peptide ionization (2,200 V, 275°C). The mass spectrometer was used in data‐dependent mode to switch consistently between MS1 and MS2 for peptide identification and multinotch‐MS3 for protein abundance measurements. Time between Masters Scans was set to 3 s. MS1 spectra were acquired with the Orbitrap in the range of *m/z* 375–1,500 at a FWHM resolution of 120,000 measured at 200 *m/z*. AGC target was set at 4.0 × 10E5 with a 50 ms maximum injection time. The more abundant precursor ions were selected for MS2 (Top speed 3 s) and collision‐induced dissociation fragmentation at 35% was performed and analyzed in the linear ion trap using the “Inject Ions for All Available Parallelizable time” option with a maximum injection time of 105 ms and an AGC target of 1.0 × 10E5. For Multi‐Notch MS3, the top 10 precursor ions from each MS2 scan were fragmented by HCD followed by orbitrap analysis (resolution = 60,000; first mass = 100 *m/z*; AGQ target = 250,000; MaxIT = 86 ms, 100–500 *m/z*). Charge state screening was enabled to include precursors with two and seven charge states. Dynamic exclusion was enabled with a repeat count of one and a duration of 60s. The mass spectrometry proteomics data have been deposited to the ProteomeXchange Consortium via the PRIDE partner repository with the dataset identifier PXD023602 (https://www.ebi.ac.uk/pride/).

#### Database search and relative quantification

Relative TMT intensity‐based quantification was processed using the freely available MaxQuant computational proteomics platform, version 1.6.3.4. Raw data were searched against the mouse database, extracted from UniProt on the 9th of December 2019, and containing 20,379 entries (reviewed) supplemented with known contaminants. Searches were performed with default parameters trypsin enzymatic cleavage with up to two missed cleavages, and three variable modifications allowed per peptide. Searches were performed with variable methionine oxidation (+15.99491), static cysteine carboxyamidomethylation (+57.02146), and static TMT modifications on lysine and the peptide N‐termini (+229.16293). The false discovery rate (FDR) at the peptide and protein levels were set to 1% and determined by searching a reverse database. For protein grouping, all proteins that cannot be distinguished based on their identified peptides were assembled into a single entry according to the MaxQuant rules. TMT reporter intensities were corrected for isotope impurities according to the lot used. The statistical analysis was done with Perseus program (version 1.6.2.1). First corrected TMT reporter intensities were base 2 logarithmized to obtain a normal distribution and normalized by subtracting the median for each TMT channel. The few proteins with missing values were removed from the dataset. To determine whether a given detected protein was specifically differential, a two‐sample *t*‐test was done using permutation‐based FDR controlled at 0.01 and employing 250 permutations. The *P*‐value was adjusted using a scale factor s0 with a value of 0.4 for the FLB1‐G4WT/‐G4KO with *n* = 5.

### Measurement of protein synthesis

The alkyne analog of puromycin “O‐propargyl‐puromycin” (OPP) was purchased from Medchem source (Federal Way, WA, USA). For *in vivo* analysis, OPP (50 mg/kg body mass; pH 6.4–6.6 in PBS) was injected intraperitoneally. One hour later, mice were euthanized, and the BM was collected. To differentiate the CD45.1 FLB1 cells from the CD45.2 host cells, 3 × 10^6^ cells isolated from the BM were pre‐stained with CD45.1 antibody at room temperature for 30 min. Cells were then washed twice in Ca^2+^‐ and Mg^2+^‐free PBS and fixed in 0.5 ml of 1% paraformaldehyde in PBS for 15 min on ice. Cells were washed in PBS, then permeabilized in 200 μl of PBS supplemented with 3% fetal bovine serum and 0.1% saponin (Sigma‐Aldrich) for 5 min at room temperature (20–25°C). To visualize the incorporation of OPP into newly translated proteins, the azide–alkyne cycloaddition was performed using the Click‐iT Cell Reaction Buffer Kit (Life Technologies, Carlsbad, CA, USA) and azide conjugated to Alexa Fluor 555 (Life Technologies, Carlsbad, CA, USA) at 5 μM final concentration. After the 30 min reaction, the cells were washed twice in PBS supplemented with 3% FBS and 0.1% saponin, then resuspended in PBS supplemented with 4′,6‐diamidino‐2‐phenylindole (DAPI; 4 μg/ml final concentration) and analyzed by flow cytometry. Relative rates of protein synthesis were calculated by normalizing OPP signals to whole bone marrow after subtracting autofluorescence background. For *in vitro* analysis, 0.5 × 10^6^ cells were seeded in 2 ml of RPMI medium containing FCS (10%). OPP (10 μM final concentration) was added to the culture medium for 1 h. Control cells consisted of PBS‐treated cells. The cells were collected, fixed, permeabilized, and the azide–alkyne cycloaddition was performed as described above. Mean OP‐Puro fluorescence reflected absolute fluorescence values for each cell population from multiple independent experiments.

### Flow cytometry

For extracellular staining, cells were incubated with appropriate antibodies (Appendix Table S1) diluted in PBS supplemented with 0.1% of FBS to block nonspecific antibody binding for 30 min at room temperature. Intracellular staining for indicated markers was performed using BD Cytofix/Cytoperm™ Fixation/Permeabilization kit (BD Biosciences; Appendix Table S1). For absolute counting of cells, 10 μl of CountBright absolute counting beads (ThermoFisher Scientific) were added to the stained cells just before acquisition on the flow cytometer. The acquisitions were performed by a BD LSRII flow cytometer or a BD Fortessa flow cytometer (BD Biosciences). Data analysis was conducted with FlowJo software (BD). For the detection of endoglin at the surface of human AML stromal cells, bone marrow samples isolated from various AML patients (see detail in Dataset EV4) were thawed in RPMI media supplemented with 10% FBS and DNAse I (10 μg/ml). After two washes with PBS, a maximum of 2 × 10^6^ cells per sample were stained as follows: dead cells were excluded using LIVE/DEAD Fixable blue staining and the cells were stained with True‐Stained Monocyte blocker and human BD FcBlock and negatively selected for CD3, CD14, CD41, CD19, CD235a, CD45, and CD31 (see detail in Appendix Table S1). The median of the intensity of endoglin fluorescence was measured in this population using Human CD105 (Endoglin) fluorescein‐conjugated antibody or its isotypic control.

### Cell cycle assays

Distribution of cell cycle phases was determined by propidium iodide (PI; Life Technologies, Carlsbad, CA, USA) staining and flow cytometry analysis. Briefly, 5 × 10^6^ FLB1‐G4 (CD45.1) cells were isolated from the bone marrow of WT or Synt‐KO mice and stained with CD45.1 antibody. Cells were fixed in 1 ml ice‐cold 70% ethanol for 30 min at 4°C and stored at −20°C. At the time of analysis, the cells were centrifuged, washed once again in PBS, and stained with a freshly made solution containing 40 μg/ml of PI, 0.1% Triton x‐100, and 40 μg/ml of RNaseA (Life Technologies, Carlsbad, CA, USA) in PBS. Percentages of cells within cell cycle compartments (G1, S, and G2/M) were determined using a FACS LSRII flow cytometer and data were analyzed with Flowjo software.

### AML cell viability

AML blasts were collected, and early/late apoptosis was measured by using the PE Annexin V apoptosis detection kit with 7‐AAD (Biolegend, San Diego, USA) according to the manufacturer. Annexin V‐positive, but 7‐AAD‐negative (early apoptotic cells) and both Annexin V‐ and 7‐AAD‐positive cells (late‐stage apoptosis) were counted with a FACS LSRII flow cytometer, and data were analyzed using Flowjo software (BD Biosciences, Franklin Lakes, USA).

### Reverse transcription‐polymerase chain reaction (RT–PCR)

RNA was extracted from cells using a Nucleospin RNA isolation kit (Macherey‐Nagel). Reverse transcription was done using PrimeScript RT Reagent with gDNA Eraser kit (TAKARA CLONTECH) and MJ Research PTC‐220 Dyad PCR System (Conquer Scientific). Quantitative PCR reactions were performed using SsoAdvanced Universal Sybr Green Supermix kit (BIORAD) and analyzed using CFX96 Touch Real‐Time PCR Detection System (BIORAD). The expression level was normalized to the housekeeping L32 and GAPDH genes. The cDNAs were amplified by 35 cycles with sets of specific primers (Appendix Table S2). Each PCR cycle consisted of 15 s of denaturation at 95°C, 30s of annealing, and extension at 60°C. Quantifications were normalized to corresponding L32 and GAPDH RNA values and expressed as relative expression using the 2(−ΔΔCT) method (Schmittgen & Livak, 2008).

### Immunofluorescence staining and confocal microscopy

For colocalization experiments, HS5 and HS27a cells were cultured for 72 h and fixed with 4% paraformaldehyde for 20 min at RT. For evaluating syntenin effects on membrane localization of endoglin, HS5 cells were treated with siRNA‐targeting syntenin (siSynt) or control (siCNT) for 48 h and fixed with 100% methanol for 5 min. For both experiments, cells were permeabilized with 0.05% saponin for 1 h. Cells were then incubated with the indicated antibodies in PBS containing 1% BSA. Cells were mounted in ProLong™ Diamond Antifade Mountant containing DAPI and observed with a Zeiss confocal microscope (LSM 880, Zeiss, France) with the corresponding lasers and 40× objectives. Confocal images were analyzed using Photoshop (Adobe, San Jose, CA) software.

### *In situ* proximity ligation assay (PLA)

PLA experiments were performed using the Duolink kits (Sigma‐Aldrich, catalog number DUO92101‐1KT) according to the manufacturer's instructions. The conjugates were prepared using the Duolink Probemaker Plus and Minus kits, syntenin antibody (ab133267, Abcam), and Endoglin antibody (sc‐18883, Santa Cruz Biotechnology). HS5 cells were fixed in 4% paraformaldehyde (PFA) for 20 min at room temperature followed by 10 min incubation in PBS‐NH4Cl (50 mM). All samples were incubated in permeabilization solution (1% BSA + 0.05% saponin) at 37°C for 1 h and then with primary antibodies (1/50 for endoglin and 1/500 for syntenin) in 1% BSA overnight at 4°C. Cells were washed twice for 5 min with “Wash Duolink buffer A” and incubated with Duolink PLA probe for 1 h at 37°C, washed in “Wash Duolink buffer A,” and incubated with Duolink ligation reagents for 30 min at 37°C. Cells were washed with “Wash Duolink buffer A” and then incubated with “Duolink amplification reagents” for 100 min at 37°C. Cells were finally washed twice for 10 min with “Duolink buffer B” and mounted in “Duolink *in situ* mounting media with DAPI.” Images were acquired with a Zeiss LSM880 laser confocal microscope using a 40× objective. To record the PLA dots throughout the entire Z‐height of the cell, Z‐stacks of 12 images with a step size of 0.5 μm were acquired. The Z‐stack was then transformed using a maximum projection. PLA dot detection was then performed. A minimum of at least five images were taken for each sample at various sites on the slide. A minimum of 200 cells per condition were analyzed. PLA produces bright red dots that were quantified using the Image J software (NIH). The tiff images were thresholded, and the function “Analyze particles” was applied in the red channel to measure the number of dots and in the blue channel to measure the number of nuclei corresponding to DAPI staining.

### Surface plasmon resonance experiments

SPR measurements were carried out at 25°C using a BIAcore T200 Instrument (GE Healthcare). A total of 100 resonance units (RU) of biotinylated ligands corresponding to the last 25 amino acids of endoglin wild‐type (SSESSSTNHSIGSTQSTPCSTSSMA) were immobilized on a streptavidin sensor chip (GE Healthcare). A 6xHis‐tagged syntenin construct (containing the two PDZ domains and the C‐terminal domain) or 6xHis‐tagged GIPC1 construct was perfused at 30 μl/min in running buffer (10 mM HEPES pH 7.4, 150 mM NaCl, and 0.005% Tween 20) at different concentrations (μM). Recombinant proteins were prepared as described previously (PMID: 27386966). The reference channel was blank immobilized. The injection time was 120 s, allowing binding to reach equilibrium. The dissociation time was 90 s. The surfaces were regenerated between runs, by short pulses of 50 mM NaOH and 1 M NaCl at 30 μl/min flow rate. Sensorgrams were corrected for binding to reference surfaces and for buffer effects (blank subtracted) before further data analysis. Apparent equilibrium dissociation constants (K_D_) were calculated by fitting the data to a simple Langmuir binding isotherm using GraphPad Prism. RUs at equilibrium were plotted as a function of protein concentration.

### Gene expression data analysis

The expression of syntenin in BMSC from mice inoculated with different subtypes of leukemia was assessed using gene expression profiling data available from the National Center for Biotechnology Information (NCBI, https://www.ncbi.nlm.nih.gov) with the accession number GSE97194. As previously described (Battula *et al*, 2017), in these experiments, murine AML cells carrying different gene rearrangements (MLL‐ENL, AML1‐ETO9a, or MLL‐ENL + FLT3‐ITD) were transplanted into C57Bl/6 mice (6‐ to 8‐week‐old). PBS was injected in the control group. Transplanted leukemia was allowed to progress for 3–4 weeks according to the disease burden. At last, mice were sacrificed and BMSC were isolated by FACS using a combination of cell surface markers (Ter119^−^, CD31^−^, CD45^−^, Sca1^+^, CD106^+^, CD105^+^, and CD140^+^). RNA was extracted, amplified, and biotinylated. Microarray data were generated using IlluminaHT12 version 4 mouse whole‐genome arrays. Data extraction and analysis were performed using the webserver GEOexplorer (https://geoexplorer.rosalind.kcl.ac.uk) as previously described (Hunt *et al*, 2022). Briefly, normal BMSC (Ctrl_expt, *n* = 3) were compared to AML‐BMSC (MLL&FLT3‐ITD_p53WT_Expt1, MLL&FLT3‐ITD_p53−/−_Expt1; AML‐ETO_Expt, *n* = 2; MLL‐ENL_Expt; *n* = 2) for differential gene expression analysis. Data were normalized and *P*‐values were adjusted using Benjamini and Hochberg procedure (Datasets EV1 and EV2). The genes that are differentially expressed were selected based on a *P*‐value < 0.01 and a difference > 2.

### Statistical analysis

For animal experiments, groups or treatments were not blinded. None of the mice was excluded from analysis. For human samples, the membrane expression of endoglin stromal cells was measured when at least 300 stromal cells were reliably detected (see details in Dataset EV4). No statistical methods were used to predetermine sample sizes. Differences between groups were determined by using one‐way or two‐way analysis of variance (ANOVA), followed by a Sidak's or Tukey's multicomparison post‐test depending on the data type, and the details can be found in data source. Single comparisons were carried out using the parametric Student's *t*‐test or the nonparametric Mann–Whitney *U*‐test. *P* < 0.05 was considered statistically significant. All statistical analyses were conducted using GraphPad Prism v10.0c software.

## Author contributions

**Raphael Leblanc:** Conceptualization; data curation; formal analysis; supervision; funding acquisition; validation; investigation; methodology; writing – original draft; writing – review and editing. **Rania Ghossoub:** Conceptualization; validation; investigation; methodology; writing – original draft; writing – review and editing. **Armelle Goubard:** Formal analysis; methodology. **Rémy Castellano:** Conceptualization; resources; formal analysis; investigation; methodology; writing – original draft. **Joanna Fares:** Conceptualization; formal analysis; funding acquisition; methodology. **Luc Camoin:** Resources; software; formal analysis; methodology. **Stephane Audebert:** Formal analysis; methodology. **Marielle Balzano:** Formal analysis; methodology. **Berna Bou‐Tayeh:** Formal analysis; methodology. **Cyril Fauriat:** Conceptualization; resources; software; formal analysis; methodology. **Norbert Vey:** Resources; investigation. **Sylvain Garciaz:** Resources; supervision; validation; investigation. **Jean‐Paul Borg:** Supervision; validation. **Yves Collette:** Supervision; validation; investigation. **Michel Aurrand‐Lions:** Conceptualization; formal analysis; supervision; validation; methodology. **Guido David:** Conceptualization; funding acquisition; validation; investigation; methodology; writing – original draft; project administration; writing – review and editing. **Pascale Zimmermann:** Conceptualization; resources; funding acquisition; validation; investigation; methodology; writing – original draft; project administration; writing – review and editing.

## Disclosure and competing interests statement

The authors declare that they have no conflict of interest.

## Supporting information



Appendix

Expanded View Figures PDF

Dataset EV1

Dataset EV2

Dataset EV3

Dataset EV4

Source Data for Appendix

PDF+

Source Data for Figure 1

Source Data for Figure 2

Source Data for Figure 3

Source Data for Figure 4

Source Data for Figure 5

Source Data for Figure 6

## Data Availability

Proteomics data have been deposited to the ProteomeXchange Consortium via the PRIDE partner repository with the dataset identifier [PXD023602] (https://www.ebi.ac.uk/pride/archive/projects/PXD023602).
