# Supplementary material for: Downregulation of stromal syntenin sustains AML development
Source: EMBO Mol Med. 2023 Oct 11;15(11):e17570. doi: 10.15252/emmm.202317570 (PMC10630886; doi:10.15252/emmm.202317570)
Supplement: Supplementary file 1 — Appendix [file EMMM-15-e17570-s009.pdf]

Leblanc et al.,
Downregulation of stromal syntenin sustains AML development.

Table of Contents

Appendix Figure S1 2

Appendix Figure S2 4

Appendix Figure S3 6

Appendix Figure S4 8

Appendix Figure S5 9

Appendix Table S1 10

Appendix Table S2 11

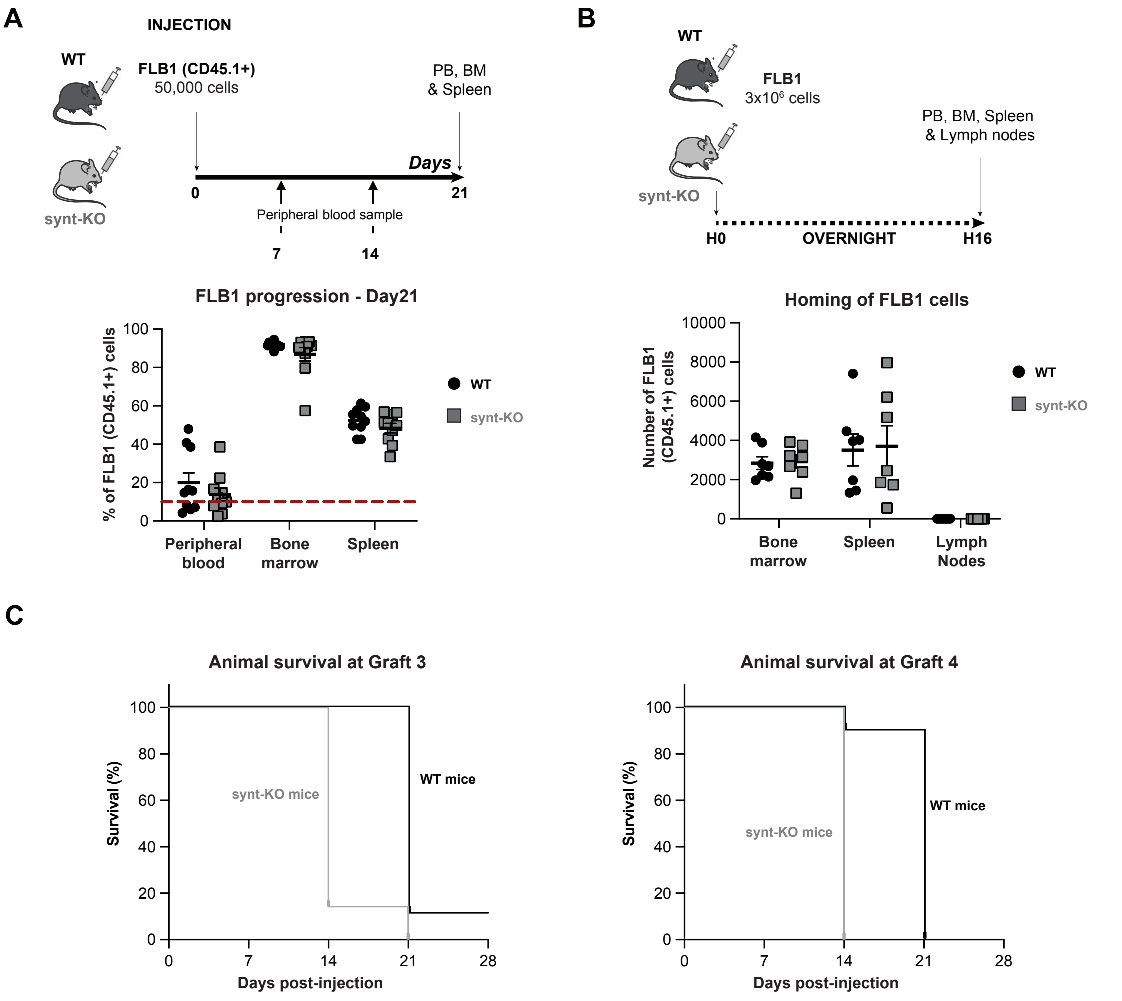

Appendix Fig. S1 (related to figure 2). Invasion and colonization of hematopoietic tissues (peripheral blood, bone marrow, spleen and lymph nodes) by FLB1 upon first transplantation (A,B) and animal survival upon serial transplantation (C), in wild-type versus syntenin-deficient animals. (A) Upper part, scheme for the invasion assay. Non-irradiated 8-11w old wild-type (WT)

and syntenin-knock-out (synt-KO) mice were injected with 50,000 FLB1 cells in the retro-orbital vein. Lower part, FACS analysis monitoring leukemia progression at day 21 in different tissues (as indicated). The dot-plot summarizes the percentage of CD45.1 FLB1 cells, relative to the total

number of CD45+ cells ± SEM, in the different organs. **(B)** Upper part, scheme for the homing assay. Non-irradiated 8-10wks old WT and KO mice were injected with 3x10^6 FLB1 cells in the retro-orbital vein. 12 hours after cell injection, animals were sacrificed and BM, spleen and lymph nodes were collected for analysis. Lower part, FACS analysis monitoring the number of FLB1 cells (using FITC-conjugated CD45.1-antibody) in different WT and synt-KO tissues as indicated. The

dot-plot indicates mean values ± SEM. **(C)** Kaplan-Meier-like plot related to **fig. 1B**, comparing the survival of WT and synt-KO mice at graft 3 and 4. All animals were sacrificed when blast levels

reached >10% in the peripheral blood. The survival curves for WT and synt-KO animals at graft 3 and 4 are significantly different using the log-rank test (***; $P<0.0001$).

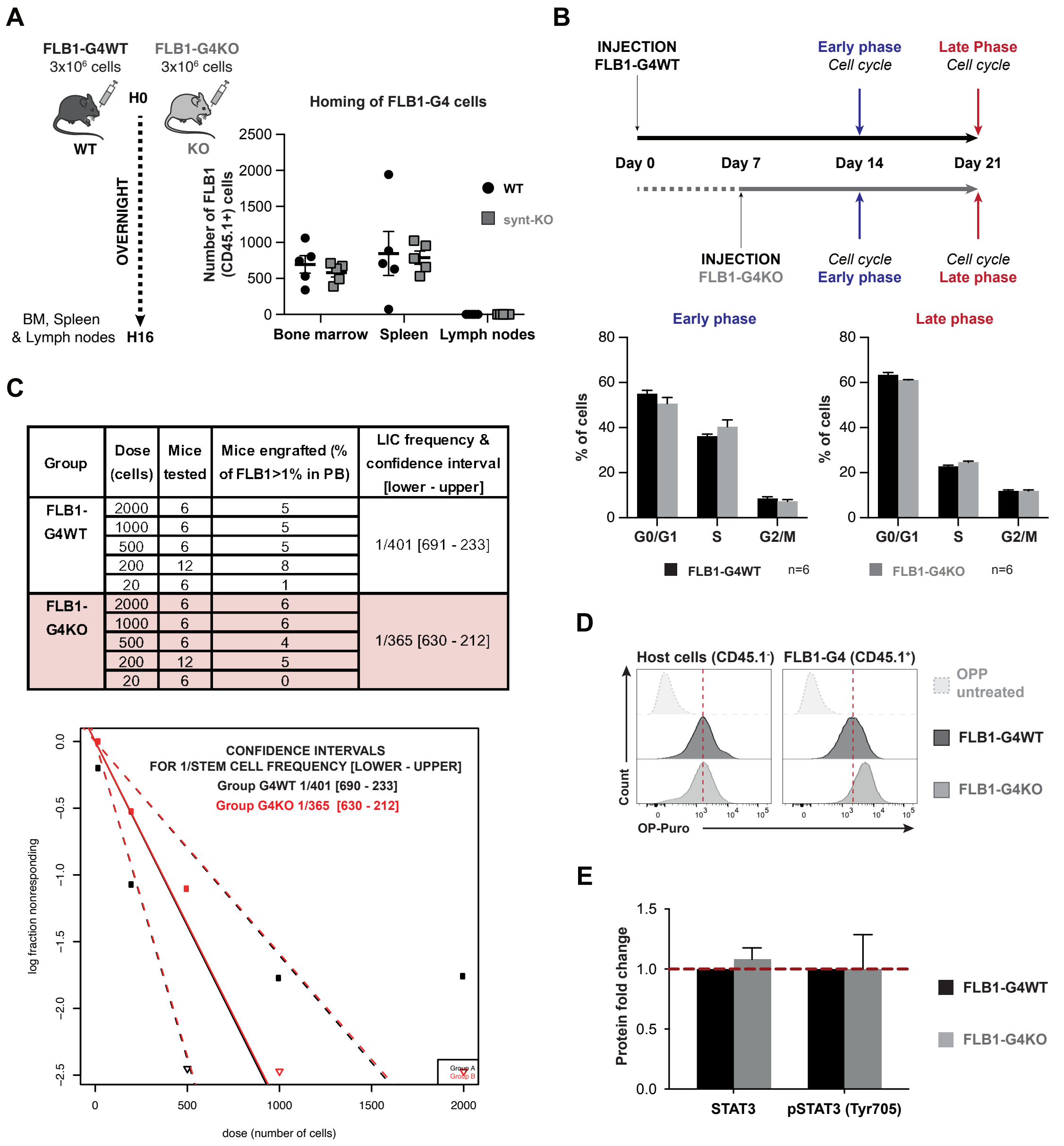

Group	Dose (cells)	Mice tested	Mice engrafted (% of FLB1>1% in PB)	LIC frequency & confidence interval [lower - upper]
FLB1-G4WT	2000	6	5	1/401 [691 - 233]
	1000	6	5	
	500	6	5	
	200	12	8	
	20	6	1	
FLB1-G4KO	2000	6	6	1/365 [630 - 212]
	1000	6	6	
	500	6	4	
	200	12	5	
	20	6	0	

Appendix Fig. S2. (related to figure 3). FLB1-G4KO display similar homing, cell cycling and clonogenic properties as FLB1-G4WT. (A) Left, scheme of the homing assay. Non-irradiated 8-10wks old WT and Synt-KO mice were injected with $3x10^6$ FLB1-G4WT or FLB1-G4KO cells in the retro-orbital vein. 16 hours after cell injection, animals were sacrificed and BM, spleen and lymph nodes were collected for analysis. Numbers of FLB1 cells in the BM and the spleen were counted by FACS, using the CD45.1-FITC antibody and CountBright™ Absolute Counting Beads according to the manufacturer. Results are expressed as mean number of CD45.1 cells per organ ± SEM. No signal was observed in lymph nodes. **(B)** Extreme Limiting Dilution Assay (ELDA) was performed to compare the frequency of Leukemia Initiating Cells (LIC) in FLB1-G4WT and

FLB1-G4KO. Upper part, table illustrating the ELDA performed by injecting decreasing numbers of sorted FLB1-G4WT or FLB1-G4KO cells, as indicated in the “dose column”. The mice were considered as engrafted when FLB1 (CD45.1) cells were detectable in the peripheral blood and composed at least 1% of the total cells in that compartment, with the numbers of successful engraftments indicated in the column “mice engrafted”. LIC frequencies for FLB1-G4WT and FLB1-G4KO were estimated, as well as the interval of confidence for each condition. Lower part, log-fraction plot illustrating the ELDA fitted to FLB1-G4WT vs FLB1-G4KO. The slope of the line represents the estimated LIC frequency. LIC frequencies of FLB1-G4WT (black line) and FLB1-G4KO (red line) are respectively 1/401 and 1/365. The dotted (black and red) lines indicate the 95% confidence intervals. **(C)** Upper part, scheme for the cell-cycling assay. FLB1-G4WT cells were injected at day 0 into WT mice, while the injection of FLB1-G4KO cells into synt-KO mice (evolving more rapidly) occurred one week later. Cell cycling was analyzed at both early (day 14) and late (day 21) phases of leukemia progression. Animals were sacrificed and their BM was stained with propidium iodide. Lower part, the cell cycle stage of the CD45.1+ cells was then analyzed by FACS. Results are expressed as percentage of cells in the different phases of the cell cycle ± SEM. **(D)** Representative histograms showing OPP incorporation into host cells ($CD45.1^-$, left) and FLB1-G4 cells ($CD45.1^+$, right) isolated from the BM of WT (black histogram), synt-KO (dark gray histogram) mice, or from the BM of a PBS-injected control mouse (OPP untreated; light gray histogram). Note the higher incorporation of OPP in FLB1-G4KO compared to FLB1-G4WT. **(E)** Histogram representing mean STAT3 and pSTAT3 signal intensities ± SEM, relative to signals obtained with FLB1-G4WT lysates, calculated from the analysis of 3 independent mice. Note, the absence of significant difference. Statistical analysis was performed using the nonparametric Mann-Whitney U test.

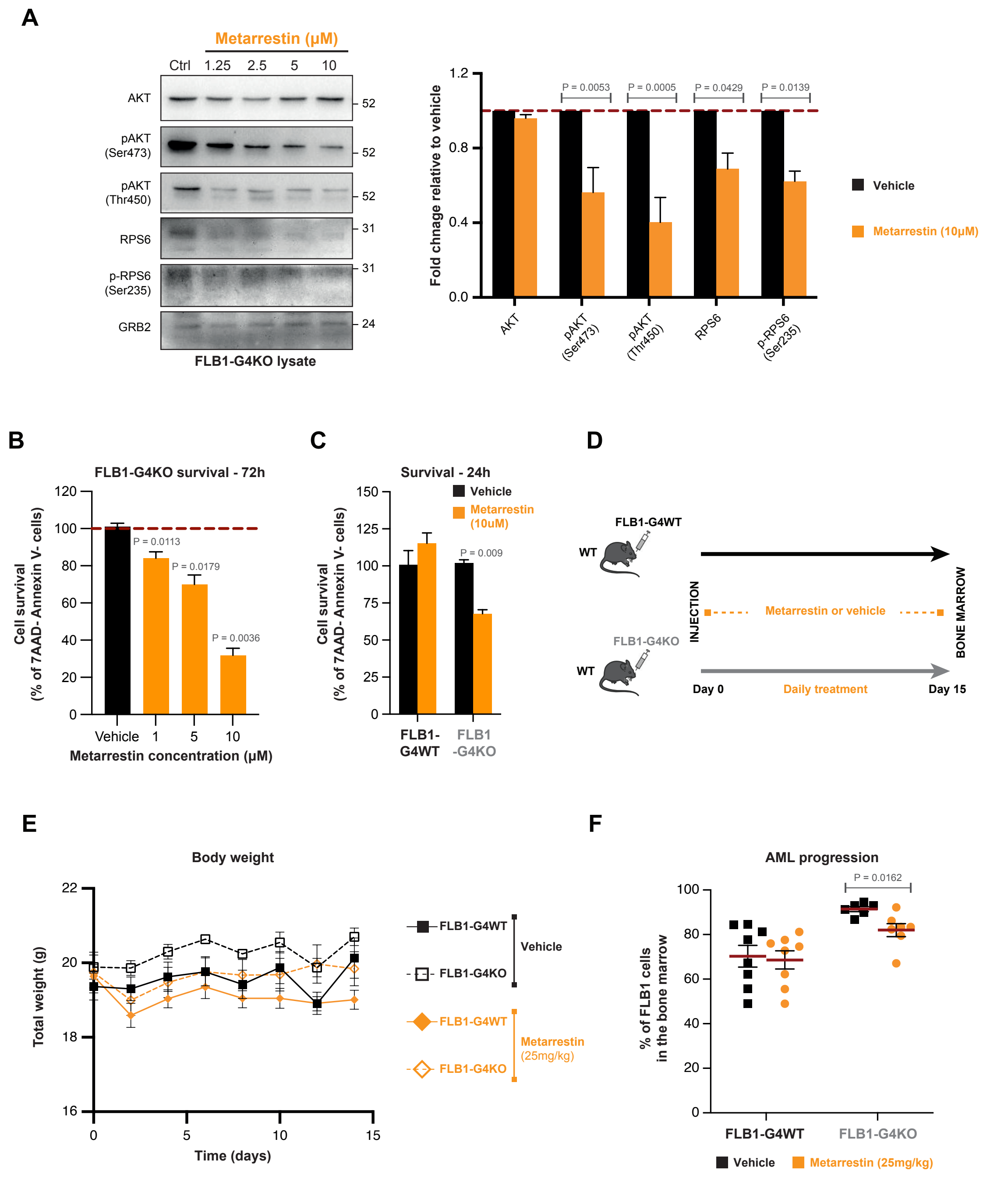

Appendix Fig. S3 (related to Fig. 3). The EEF1A2 inhibitor Metarrestin diminishes the *in vitro* survival and in vivo aggressiveness of leukemia blasts educated by a syntenin-deficient host.

(A) Left, Western blots of total cell lysates from FLB1-G4KO treated with DMSO (Ctrl) or metarrestin (1.25 to 10µM) for 16h. Different markers of cell survival were analyzed, as indicated. GRB2 was used as control. Right, histograms representing the mean signal intensities in drug-treated cells relative to the signals in FLB1-G4KO cells treated with vehicle (DMSO) ± SEM, calculated from the analysis of 3 independent experiments. Statistical analysis was performed

using the two-way analysis of variance (ANOVA). **(B)** Effect of Metarrestin treatment on FLB1-G4KO cell survival as determined in FACS. Cell survival in the presence of increasing concentrations of metarrestin was evaluated at 72h. Data represent the mean percentages of living FLB1-G4KO (CD45.1) cells ± SEM calculated from 3 independent experiments performed in triplicate. Statistical analysis was performed using the two-way analysis of variance (ANOVA). (C) FLB1-G4WT and FLB1-G4KO cells were treated with DMSO (control) or 10µM of metarrestin for 24h. Data represent the percentage of viable (annexinV-, 7AAD-) cells normalized to control ± SEM, calculated from 3 independent experiments performed in triplicate. Statistical analysis was performed using the one-way analysis of variance (ANOVA). **(D)** Scheme of the in vivo Metarrestin experiments. FLB1-G4WT cells were injected at day 0 into WT mice, while the inoculation of FLB1-G4KO cells into KO mice was delayed for one week, in order to synchronize the treatment. Animals were daily treated with Metarrestin or vehicle. Leukemia progression was assessed weekly, by FACS analysis as previously reported. **(E)** Daily evaluation of the body weight of each animal, treated with Metarrestin or vehicle. **(F)** FLB1 cell (CD45.1) frequencies in bone marrow aspirates were measured 15 days after cell inoculation. Results are expressed as mean percentage of FLB1 cells in the bone marrow ± SEM. Statistical analysis was performed using Unpaired t-test.

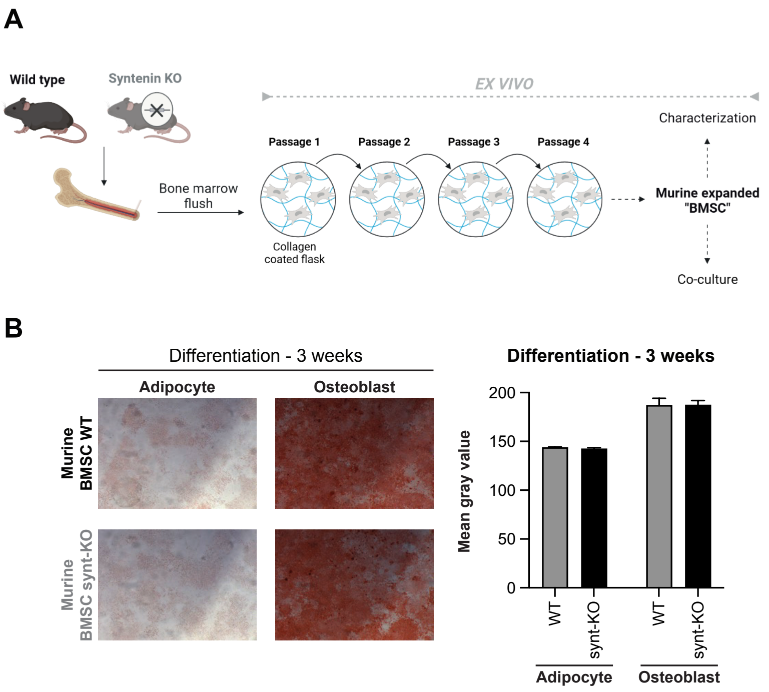

Appendix Fig. S4 (related to figure 4). Murine BMSC isolation and characterization. (A) Scheme illustrating the protocol for the preparation of murine expanded BMSC. 5-6w old WT and synt-KO mice were sacrificed, hind limbs were collected, and bones were flushed. Whole BM was plated on collagen coated flasks and expanded *ex vivo*. Hematopoietic cells were gradually eliminated by several washes, medium changes and at least four passages. **(B)** Characterization of the differentiation potential of BMSC. Expanded BMSC, WT and synt-KO, at passage 4 were cultivated under specific osteogenic or adipogenic conditions for 21 days. Alizarin Red and Red oil staining were performed to assess, respectively, Ca2++ deposition after osteogenic induction and lipid droplet accumulation after adipocyte differentiation. Note the similar differentiation profiles of WT and synt-KO BMSCs. The histograms represent the mean gray levels quantified with Image J from 5-6 images per condition.

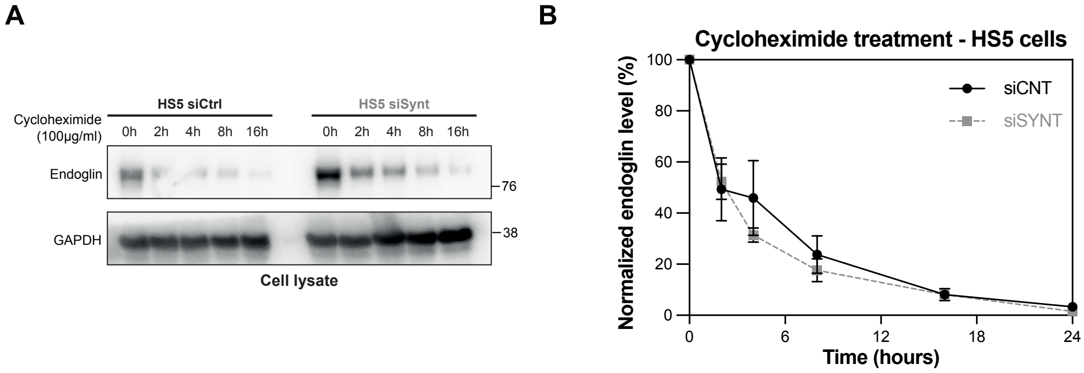

Appendix Figure S5 (related to Figure 5). Syntenin does not affect endoglin protein stability. (A) HS5 cells transfected with siRNA directed against syntenin (siSynt) or control (siCtrl) were treated with cycloheximide (100 µg/ml) and harvested at specific time points (0, 2, 4, 8, 16, and 24 h). Cells were then lysed and analyzed by Western blot for indicated markers. **(B)** Graphs representing mean endoglin signal intensities ± SEM, relative to GAPDH and normalized to time 0 for each condition, calculated from the analysis of 3 independent experiments.

REAGENT OR RESOURCE	NAME	SOURCE	REFERENCE	USE	DILUTION/CONCENTRATION
**Antibodies**	CD45.1 (Ly-5.1) clone A20, FITC	BD Biosciences	564704	Flow cytometry	1/100
	CD45.1 (Ly-5.1) clone A20, PE CF594	BD Biosciences	562452	Flow cytometry	1/100
	CD45.2 (Ly-5.2) clone 104, PerCP-Cyanine5.5	eBioscience	45-0454-82	Flow cytometry	1/500
	CD45.2 (Ly-5.2) clone 104, PE	BioLegend	109808	Flow cytometry	1/500
	TER-119 (Ly-76), PE	BioLegend	116208	Flow cytometry	1/500
	CD3e clone 17A2, PE	BioLegend	100206	Flow cytometry	1/800
	CD4 clone RM4-5, PE	eBioscience	12-0043-82	Flow cytometry	1/500
	CD8a clone 53-6.7, PE	eBioscience	12-0081-82	Flow cytometry	1/500
	Gr1 (Ly6G) cloneRB6-8C5, PE	eBioscience	12-5931-81	Flow cytometry	1/100
	CD51 (Integrin alpha V) clone RMV-7, Biotin	eBioscience	13-0512-81	Flow cytometry	1/200
	Ly-6A/E (Sca-1) clone D7, BV421	BD Biosciences	562729	Flow cytometry	1/600
	CD54 (ICAM-1) clone 3E2, BV421	BD Biosciences	553332	Flow cytometry	1/100
	CD106 (VCAM-1) clone 429, FITC	BD Biosciences	553775	Flow cytometry	1/100
	CD44 clone IM7, PerCP-Cyanine5.5	eBioscience	45-0441-82	Flow cytometry	1/200
	CD295 (Leptin receptor)	R&D	BAF497	Flow cytometry	1/100
	CD44, clone IM7, PerCP-Cyanine5.5	eBioscience	45-0441-82	Flow cytometry	1/200
	Mouse CD105 (Endoglin), clone MJ7/18, PECF594	BD Biosciences	562762	Flow cytometry	1/500
	Human CD45 clone HI30, Pacific Blue	BioLegend	304021	Flow cytometry	1/20
	Human CD105 (Endoglin) Flurorescin-conjugated Antibody	R&D	FAB10971F	Flow cytometry	1/20
	Mouse IgG1 Isotype control	R&D	MAB002	Flow cytometry	1/20
	Human CD3 clone UCHT1, Spark Violet 538	BioLegend	300483	Flow cytometry	1/80
	Human CD14 clone M5E2, Alexa Fluor 700	BD Biosciences	557923	Flow cytometry	1/50
	Human CD41 clone P2, PE	Beckman Coulter	A07781	Flow cytometry	1/50
	Human CD19 clone SJ25C1, PE/Cyanine5	BioLegend	363041	Flow cytometry	1/50
	Human CD45 clone HI30, PerCP-eFluor 710	eBioscience	46-0459-42	Flow cytometry	1/25
	Human CD235a clone HIR2, APC	BD Pharmingen	551336	Flow cytometry	1/100
	Human CD31 clone WM59, Brilliant Violet 421	BioLegend	303124	Flow cytometry	1/50
	GAPDH	Proteintech	10494-1-AP	Western blot	1/5000
	Tubulin clone DM1A	Sigma Aldrich	T6199	Western blot	1/10000
	GRB2	BD Biosciences	610112	Western blot	1/1000
	Syntenin clone 96	Homemade	-	Western blot	1/1000
	Alix clone 67	Homemade	-	Western blot	1/500
	Flotillin-1 clone 18	BD Biosciences	610820	Western blot	1/1000
	TSG101, clone C2	Santa Cruz	sc-7964	Western blot	1/1000
	Endoglin A8	Santa Cruz	sc-376381	Western blot	1/50
	Endoglin P3D1	Santa Cruz	sc-18883	Confocal microscopy	1/50
	Syntenin EPR8102	Abcam	ab133267	Confocal microscopy	1/500
	EEF1A2	GeneTex	GTX102326	Western blot, Flow cytometry	1/100
	RICTOR clone 53A2	Cell signaling	2114	Western blot	1/1000
	pT1135 RICTOR clone D30A	Cell signaling	3806	Western blot	1/1000
	AKT	Cell signaling	9272	Western blot	1/1000
	pS473 AKT clone D9E	Cell signaling	4060	Western blot, Flow cytometry	1/2000, 1/100
	pT450 AKT	Cell signaling	9267	Western blot	1/1000
	RPS6 clone 54D2	Cell signaling	2317	Western blot	1/1000
	pS235/236 RPS6 clone D572.2E	Cell signaling	4858	Western blot, Flow cytometry	1/2000, 1/50
	Donkey anti-rabbit IgG secondary antibody Alexa Fluor 488	Life Technologies	A21206	Confocal microscopy	1/2000
	Donkey anti-rabbit IgG secondary antibody Alexa Fluor 555	Life Technologies	A31572	Confocal microscopy, Flow cytometry	1/2000
	Goat anti-rabbit IgG secondary antibody APC	Life Technologies	A10931	Flow cytometry	1/2000
**Chemicals & proteins**	Collagen 1, Rat tail	Life Technologies	A1048301	-	5µg/cm2
	Insulin human, recombinant	Sigma Aldrich	I2643-25MG	-	10µg/ml
	Dexamethasone	Sigma Aldrich	D4902-25MG	-	1µM
	3-Isobutyl-1-methylxanthine	Sigma Aldrich	I5879-100MG	-	500µg/ml
	β-Glycerophosphate disodium salt hydrate	Sigma Aldrich	G9422-10G	-	10mM
	L-Ascorbic acid	Sigma Aldrich	A5960-10MG	-	50µg/ml
	DAPI fluoromount G	Cliniscience	0100-20	Immunofluorescence	1/2000
	Alexa Fluor 555 Azide 0.5mg	Life Technologies	A20012	Flow cytometry	5µM
	LIVE/DEAD® Fixable Far Red Dead Cell Stain	Life Technologies	L10120	Flow cytometry	1/1000
	Draq 7	Biolegend	424001	Flow cytometry	1/400
	SYTOX Blue Nucleic Acid Stain	Life Technologies	S11348	Flow cytometry	500nM
	Live/DEAD® Fixable Blue	Life Technologies	L23105	Flow cytometry	1/1000
	Propidium iodide (PI)	eBioscience	BMS500PI	Flow cytometry	40µg/ml
	CountBright™ Absolute Counting Beads	Life Technologies	C36950	Flow cytometry	1/100
	True-Stain Monocyte Blocker	BioLegend	426102	Flow cytometry	1/50
	Human BD FcBlock	BD Pharmingen	564220	Flow cytometry	1/50
	Oil Red O solution , 0.5% in isopropanol	Sigma Aldrich	O1391-250ML	-	-
	Alizarin-red staining solution	Sigma Aldrich	TMS-008-C	-	-
	O-Propargyl-puromycin	Jena Bioscience	NU-931-5	-	10µM (*in vitro*), 50mg/kg (*in vivo*)
	Annexin V Apoptosis detection kit PE	Life Technologies	88-8102-72	Flow cytometry	1/50
	Annexin V Apoptosis detection kit APC	Life Technologies	88-8007-72	Flow cytometry	1/50
	Click-it cell reaction buffer	Life Technologies	C10269	Flow cytometry	-
	Metarrestin (ML-246)	Cliniscience	AOB1384	-	10µM (*in vitro*), 25mg/kg (*in vivo*)

Appendix Table S1. Antibodies and reagents

Species	Primers	Forward (Fw) / Reverse (Rv)	Sequence 5'-3'
Human	SDCBP	Fw	AAGGTGCTCAAACAGGCTTT
		Rv	AAACCAACATGTCCAGTGCT
	GAPDH	Fw	TGCCAAATATGATGACATCAAGAA
		Rv	GGAGTGGGTGTCGCTGTTG
	Endoglin	Fw	CACTAGCCAGGTCTCGAAGG
		Rv	CTGAGGACCAGAAGCACCTC
	L32	Fw	CAAGGAGCTGGAAGTGCTGC
		Rv	CAGCTCTTTCCACGATGGC

Appendix Table S2. List of primers for RT-qPCR analysis.
