# Supplementary material for: Downregulation of stromal syntenin sustains AML development
Source: EMBO Mol Med. 2023 Oct 11;15(11):e17570. doi: 10.15252/emmm.202317570 (PMC10630886; doi:10.15252/emmm.202317570)
Supplement: Supplementary file 2 — Expanded View Figures PDF [file EMMM-15-e17570-s010.pdf]

Expanded View Figures

Figure EV1. (Related to Fig 5). Syntenin–endoglin relationship.

Effect of loss of syntenin on endoglin expression (A–D) and endoglin–syntenin interaction assays (E–G).

A Representative confocal micrographs showing the subcellular distribution of endogenous endoglin in HS5 and HS27a cells, depleted for syntenin expression (siSynt) or treated with control siRNA (siCtrl). Nuclei are stained with DAPI (blue), and endoglin is in green.

B Real-time PCR analysis of endoglin mRNA expression in HS5 and HS27a cells depleted for syntenin (siSynt) and endoglin (siEng) or treated with control siRNA (siCtrl). Values were normalized to housekeeping L32/GAPDH genes. Data represent the mean ± SEM of four independent experiments performed in duplicate.

C Left, FACS analysis of endoglin levels in HS5, transfected with miR-155-5p (miR-155) or control (miR-Ctrl). Cells were immunostained with the anti-human endoglin monoclonal antibody or isotype control antibody (untreated). Right, data represent the mean of the means of fluorescence intensity ± SEM, collected from three independent experiments. Statistical analysis was performed using the nonparametric Mann–Whitney *U*-test.

D HS5 cells were transfected with miR-155-5p (miR-155) or control (miR-Ctrl). After 48 h, the media were changed and the transfected HS5 cells were cultured in medium containing EV-depleted FCS (10%) for 16 h. Conditioned medium was submitted to differential centrifugation and small EVs were pelleted at 100,000 *g*. Total cell lysates and corresponding pelleted extracellular particles (small EVs) were analyzed for indicated markers.

E Surface plasmon resonance experiment illustrating the direct interaction of endoglin with GIPC1, used as positive control. Sensorgrams illustrating the binding of GIPC1, at increasing concentrations of recombinant GIPC1 (0.5 μM to 120 μM), to an immobilized peptide corresponding to the last 25 C-terminal amino acids of wild-type endoglin.

F Langmuir graph showing the binding (in resonance units, RU) of recombinant syntenin (blue curve) and GIPC1 (red curve) as observed at equilibrium, at increasing concentrations of analyte. KD (apparent) was calculated from the protein concentration required to observe half of the maximal response.

G Proximity ligation assay (PLA) detecting the proximity of endogenous syntenin and endoglin in HS5 cells. Left, representative images of confocal microscopy showing the PLA signals. Nuclei were stained with DAPI. Syntenin and endoglin antibodies were incubated alone or together. Each picture is representative of a typical cell staining pattern observed in five fields chosen at random. Right, the quantification of the number of PLA dots per nucleus is represented as the mean values ± SEM from a single experiment performed in duplicate. Statistical analysis was performed using the one-way analysis of variance (ANOVA).

© 2023 The Authors

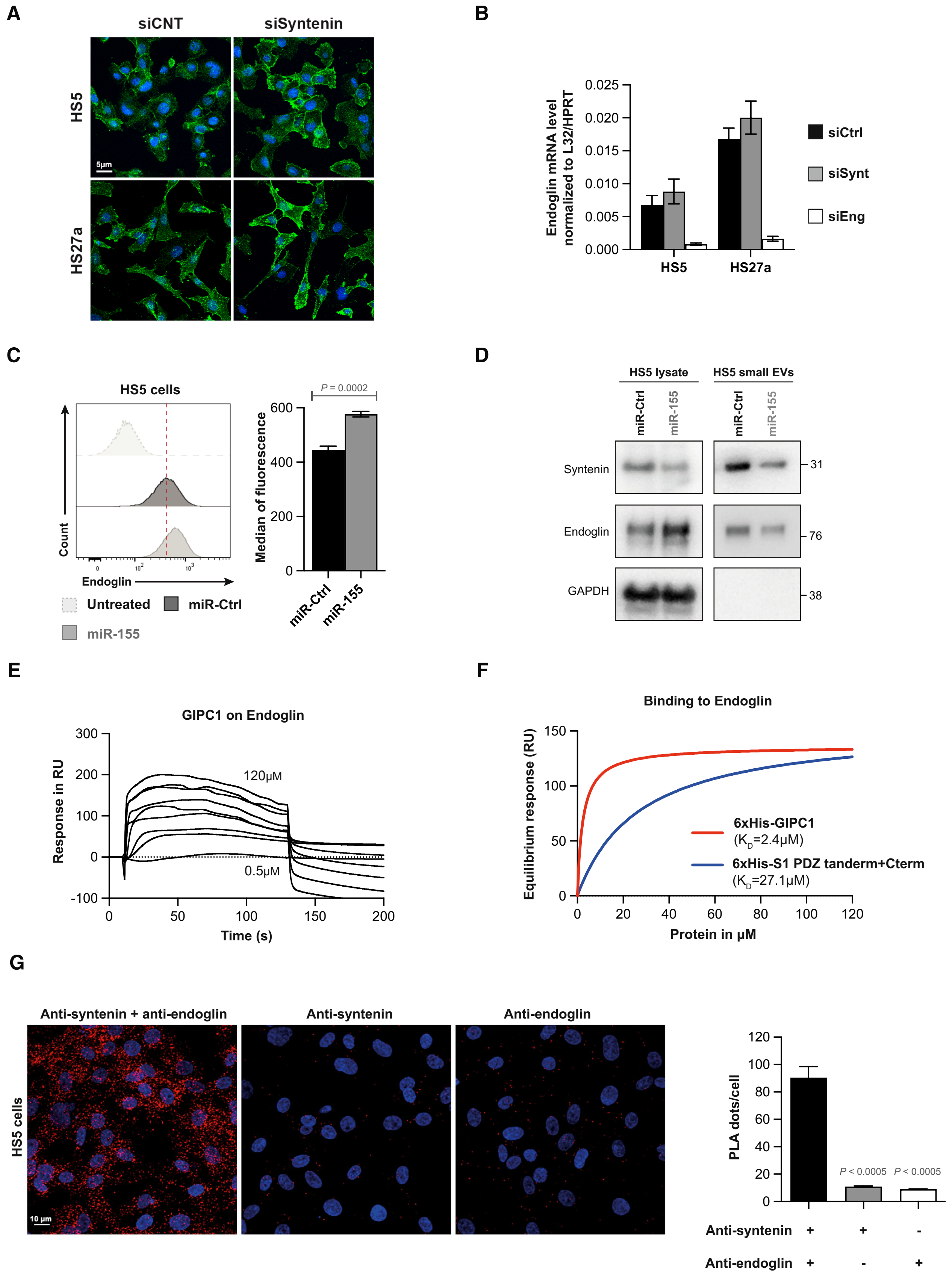

Figure EV1.

 © 2023 The Authors

Figure EV2. (Related to Fig 6). Endoglin/syntenin suppression in HS5 cells (control transfection experiments).

A Western blots of the lysates of HS5 cells transfected with control siRNA (siCtrl), with control siRNA and siRNA-targeting syntenin (siSynt + siCtrl) or endoglin (siEng + siCtrl), or siRNA-targeting syntenin and endoglin (siSynt + siEng; 30 nM), showing the effects on syntenin levels. GAPDH was used as control.

B FACS analysis of the endoglin expression at the surface of HS5 cells transfected with control siRNA (siCtrl, dark gray), with control siRNA and siRNA-targeting syntenin (siSynt + siCtrl, light gray) or endoglin (siEng + siCtrl, blue), or siRNA-targeting syntenin and endoglin (siSynt + siEng; red). Isotype control antibody was used as control (unstained).

C HS27a (stroma) cells were transfected with siRNA-targeting syntenin (siSynt), endoglin (siEng), both (siSynt + siEng), or control siRNA (siCtrl). After 24 h, HL60 (leukemia) cells were put in contact with the transfected HS27a cells. After 1 week of co-culture, HL60 cells were collected and re-seeded on freshly transfected HS27a cells. After 3 weeks of co-culture, AML cells were collected and treated with O-propargyl-puromycin (OPP) to measure protein synthesis by FACS. Upper panel, FACS detection of OPP incorporation in HL60 cells educated on HS27a treated as indicated. Untreated refers to HL60 cells not treated with OPP. Lower panel, graph representing the relative levels of protein synthesis in HL60 cells, normalized to untreated HL60 cells ± SEM. Values were calculated from four independent experiments. Statistical analysis was performed using the one-way analysis of variance (ANOVA).

D–F HS5 cells were transfected with siRNA-targeting syntenin (siSynt), endoglin (siEng), both (siSynt + siEng), or control siRNA (siCtrl). Twenty-four hours later, human AML HL60, U937, or OCI-AML3 cells were co-cultured with these HS5 cells. After 1 week of co-culture, AML cells were collected and re-seeded on freshly transfected HS5 cells. After 3 weeks of co-culture, AML cells were collected for FACS analysis of (D) EEF1A2, (E) pAKT (Ser473), and (F) pRPS6 (Ser235), respectively. The histograms represent the fold change in mean of fluorescence intensity relative to the signal in AML cells maintained on HS5 cells transfected with siCtrl ± SEM calculated from the analysis of three independent experiments. Statistical analysis was performed using the nonparametric two-way analysis of variance (ANOVA).

© 2023 The Authors

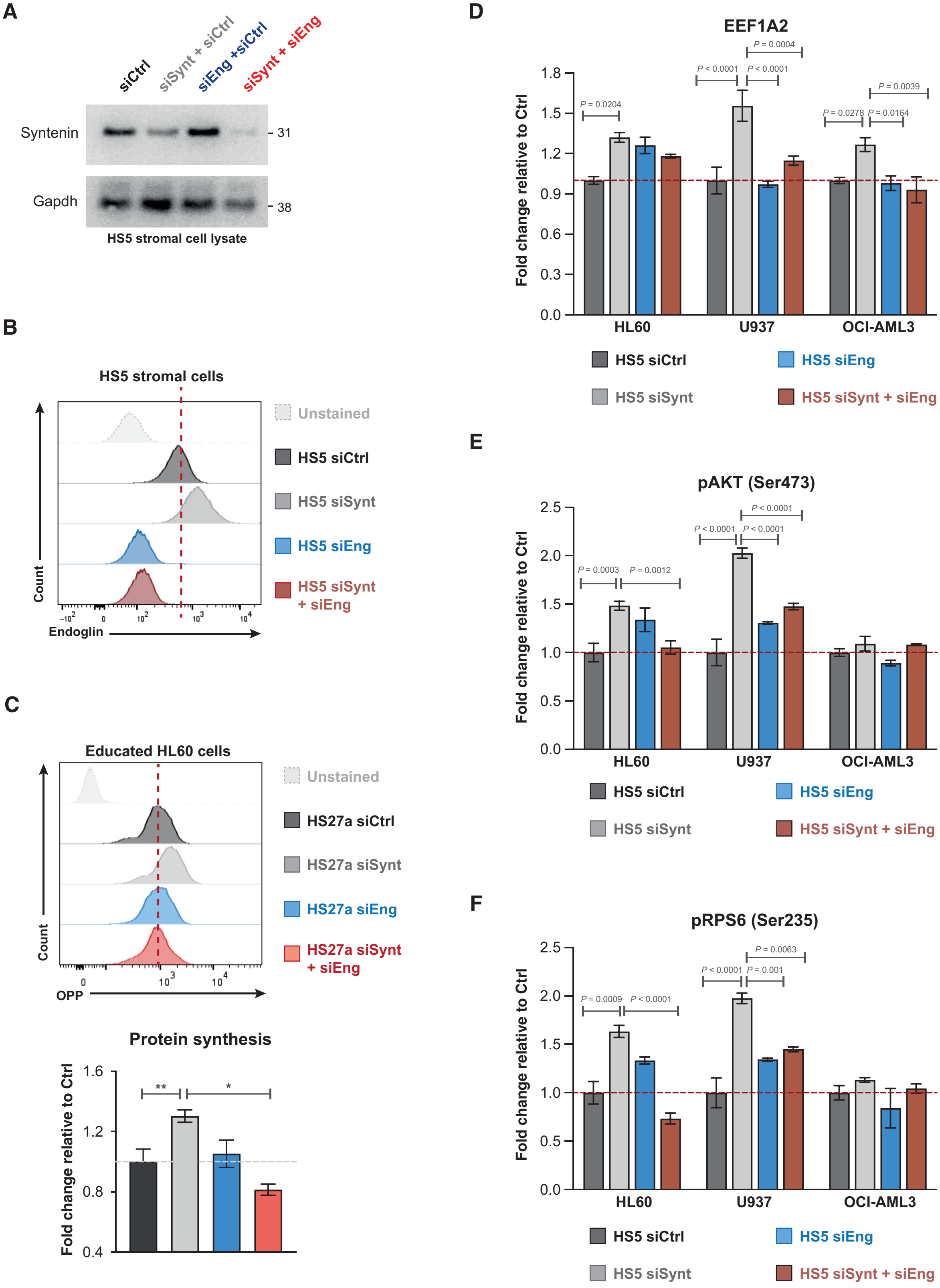

Figure EV2.

 © 2023 The Authors
