# Supplementary figures and images for: Downregulation of stromal syntenin sustains AML development
Source: EMBO Mol Med. 2023 Oct 11;15(11):e17570. doi: 10.15252/emmm.202317570 (PMC10630886; doi:10.15252/emmm.202317570)

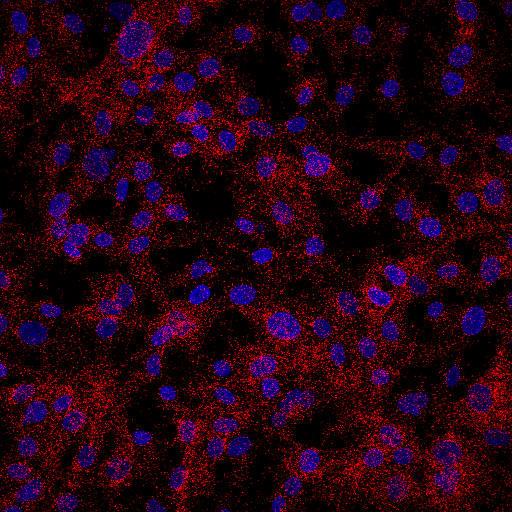

Supplement: Supplementary file 7 — Source Data for Appendix [file EMMM-15-e17570-s007.zip › Appendix data source/Confocal microscopy figure S5/Figure S5G/Anti-syntenin + anti-endoglin.tif]

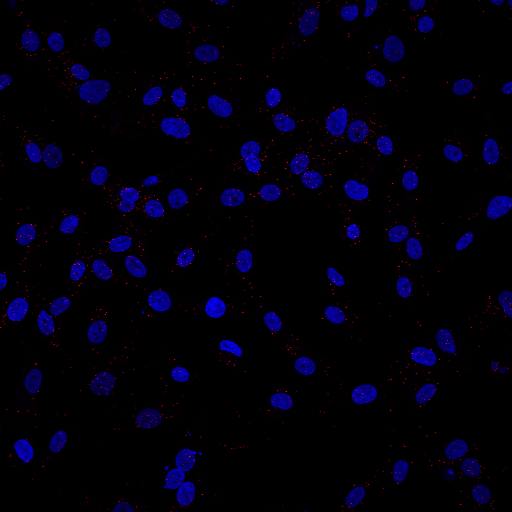

Supplement: Supplementary file 7 — Source Data for Appendix [file EMMM-15-e17570-s007.zip › Appendix data source/Confocal microscopy figure S5/Figure S5G/Anti-syntenin.tif]

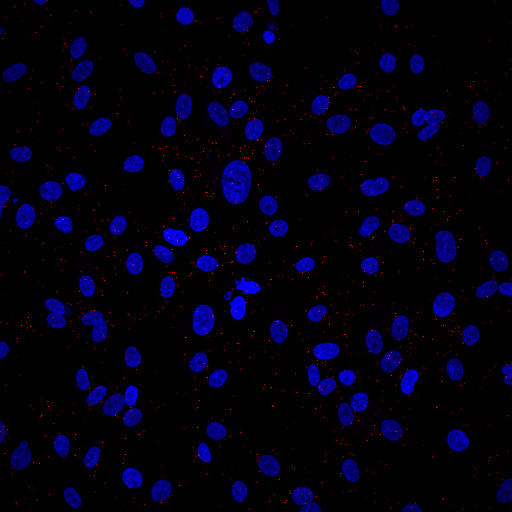

Supplement: Supplementary file 7 — Source Data for Appendix [file EMMM-15-e17570-s007.zip › Appendix data source/Confocal microscopy figure S5/Figure S5G/Anti-enddoglin.tif]

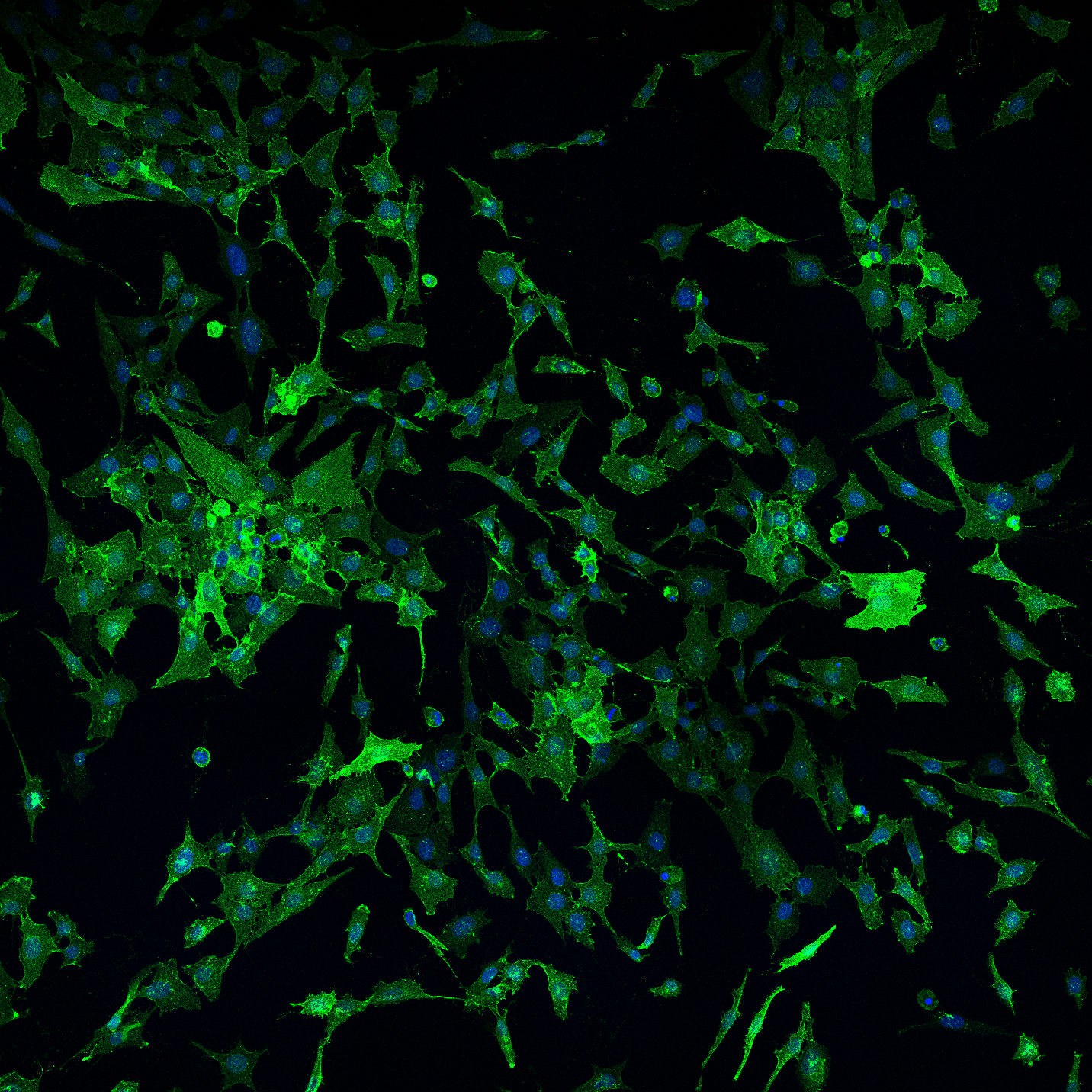

Supplement: Supplementary file 7 — Source Data for Appendix [file EMMM-15-e17570-s007.zip › Appendix data source/Confocal microscopy figure S5/Figure S5A/HS27A siCNT/Image 5-Orthogonal Projection-19.tif]

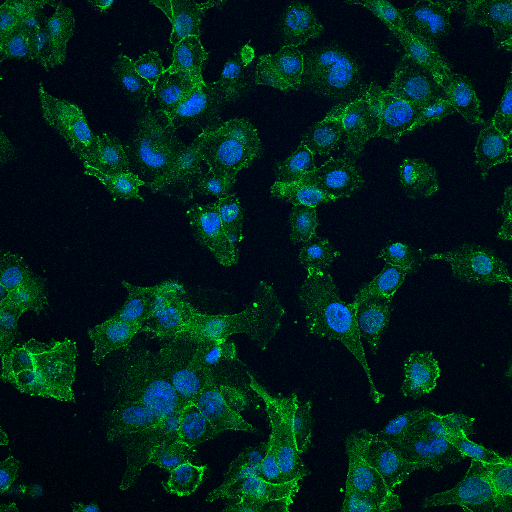

Supplement: Supplementary file 7 — Source Data for Appendix [file EMMM-15-e17570-s007.zip › Appendix data source/Confocal microscopy figure S5/Figure S5A/HS5 siCNT/Image 19.tif]

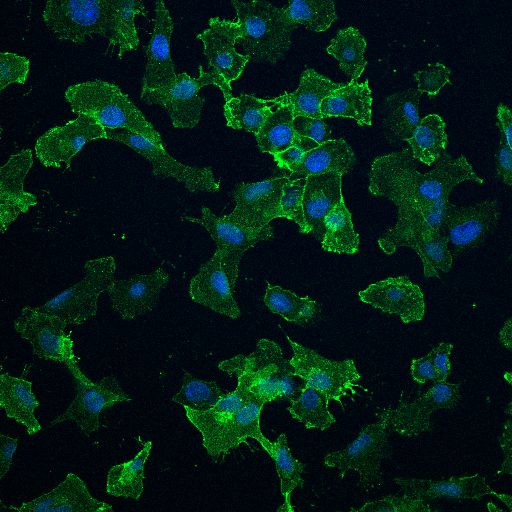

Supplement: Supplementary file 7 — Source Data for Appendix [file EMMM-15-e17570-s007.zip › Appendix data source/Confocal microscopy figure S5/Figure S5A/HS5 siSyntenin/Image 9.tif]

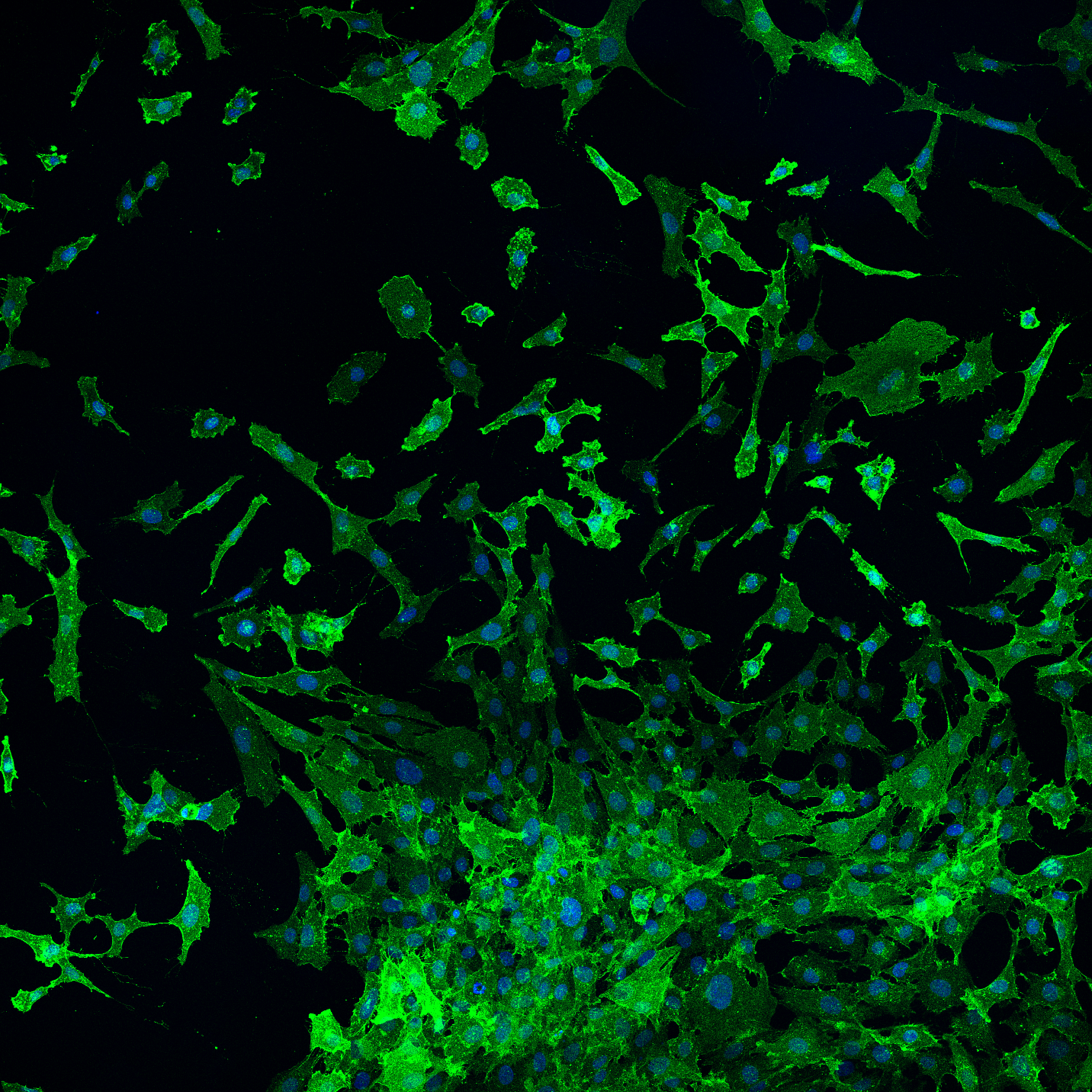

Supplement: Supplementary file 7 — Source Data for Appendix [file EMMM-15-e17570-s007.zip › Appendix data source/Confocal microscopy figure S5/Figure S5A/HS27a siSyntenin/Image 2-Orthogonal Projection-21.tif]

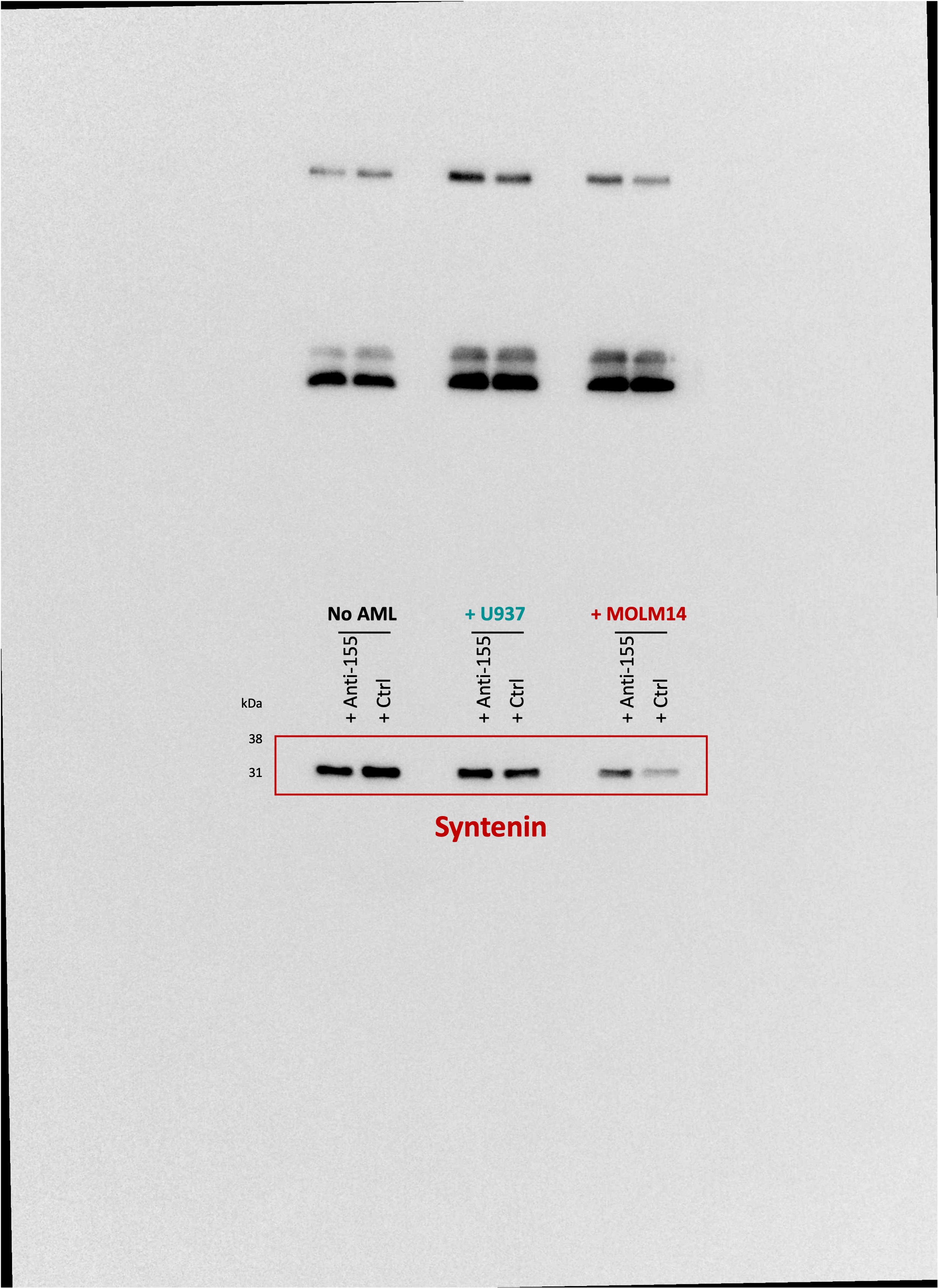

Supplement: Supplementary file 9 — Source Data for Figure 1 [file EMMM-15-e17570-s015.zip › Data source Figure 1/Western blot Figure 1E/Fig1E Syntenin.jpg]

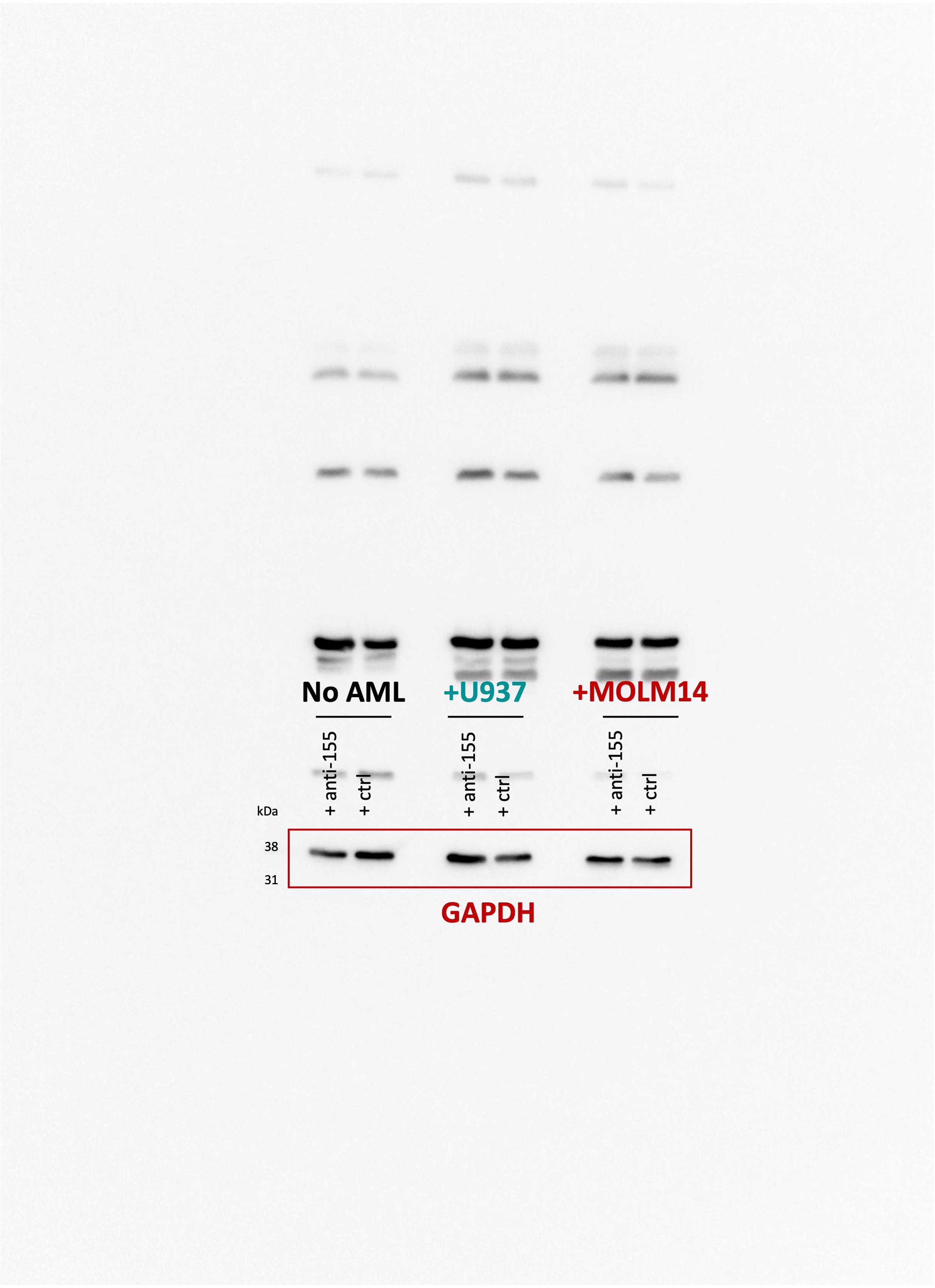

Supplement: Supplementary file 9 — Source Data for Figure 1 [file EMMM-15-e17570-s015.zip › Data source Figure 1/Western blot Figure 1E/Fig1E GAPDH.jpg]

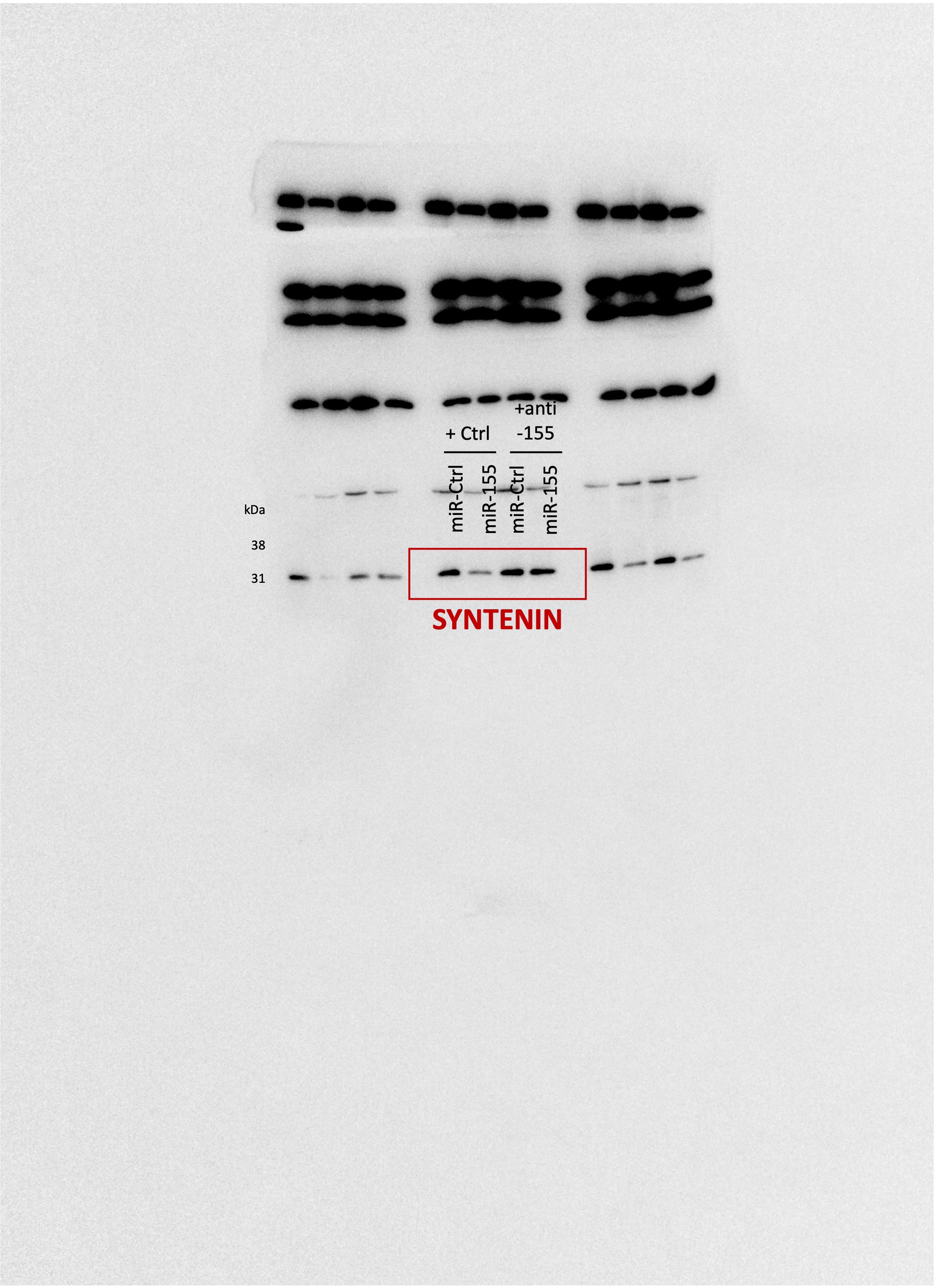

Supplement: Supplementary file 9 — Source Data for Figure 1 [file EMMM-15-e17570-s015.zip › Data source Figure 1/Western blot Figure 1D/Fig1D Syntenin.jpg]

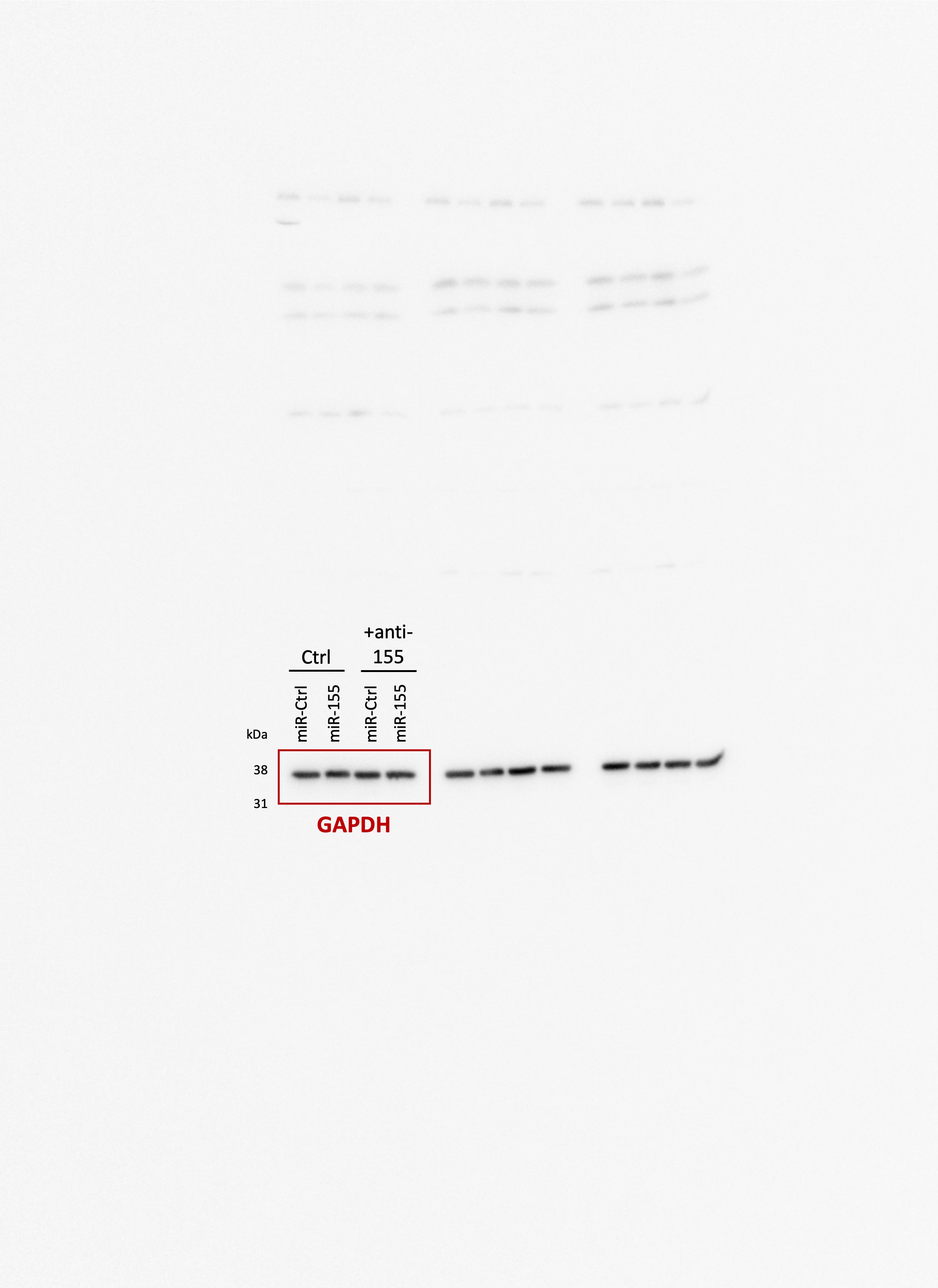

Supplement: Supplementary file 9 — Source Data for Figure 1 [file EMMM-15-e17570-s015.zip › Data source Figure 1/Western blot Figure 1D/Fig1D GAPDH.jpg]

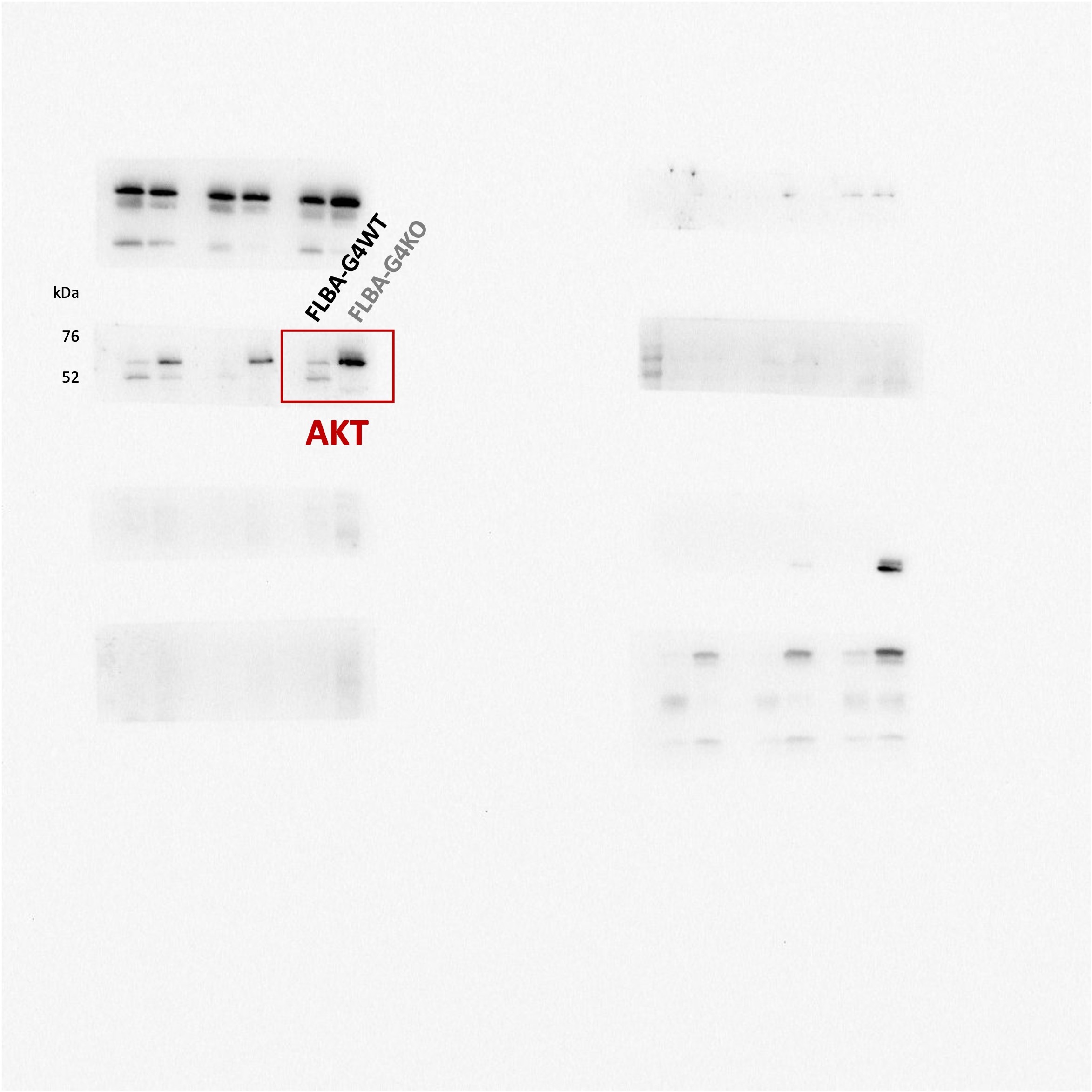

Supplement: Supplementary file 11 — Source Data for Figure 3 [file EMMM-15-e17570-s002.zip › Data source Figure 3/Western blot Figure 3E/Fig3E AKT.jpg]

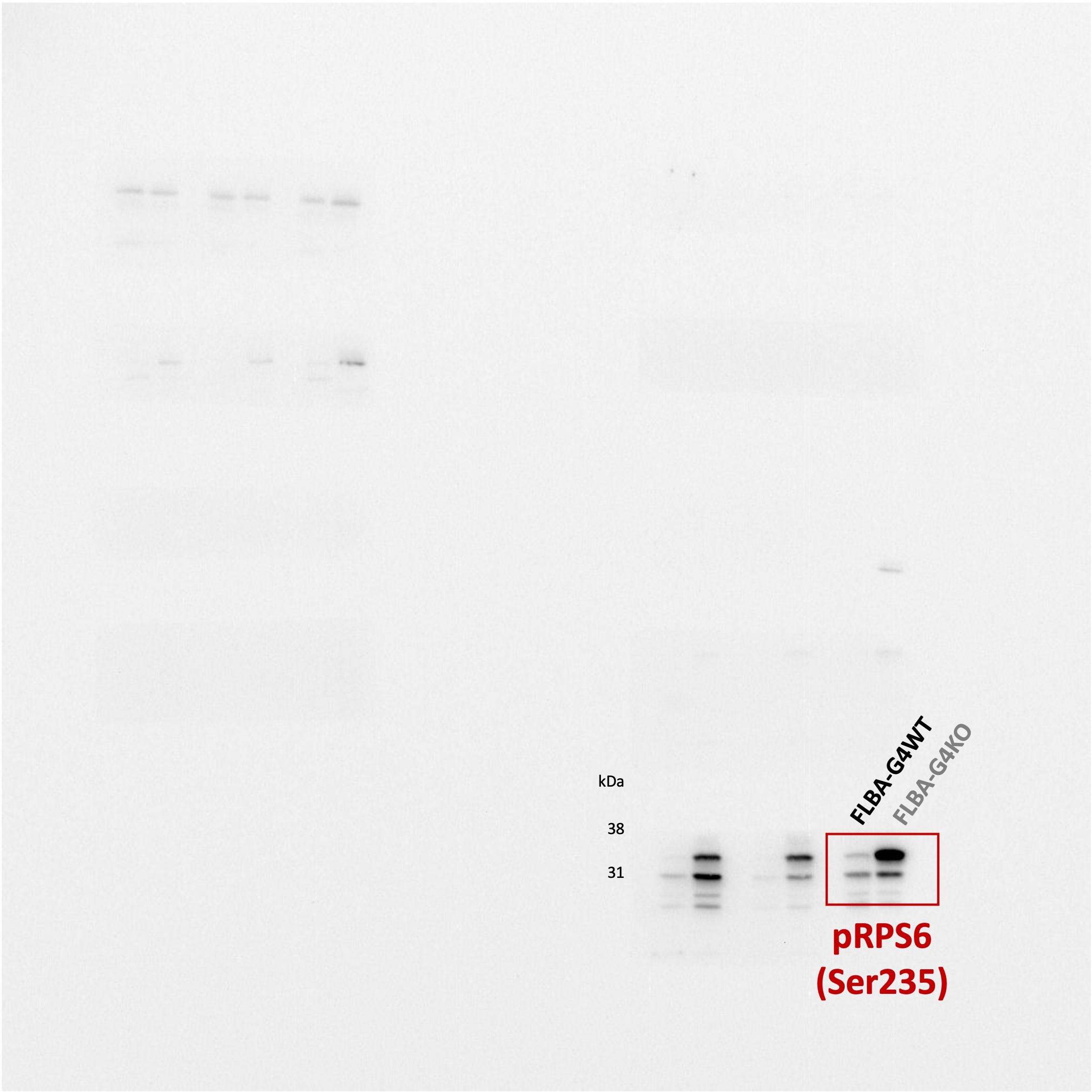

Supplement: Supplementary file 11 — Source Data for Figure 3 [file EMMM-15-e17570-s002.zip › Data source Figure 3/Western blot Figure 3E/Fig3E pRPS6 (Ser235).jpg]

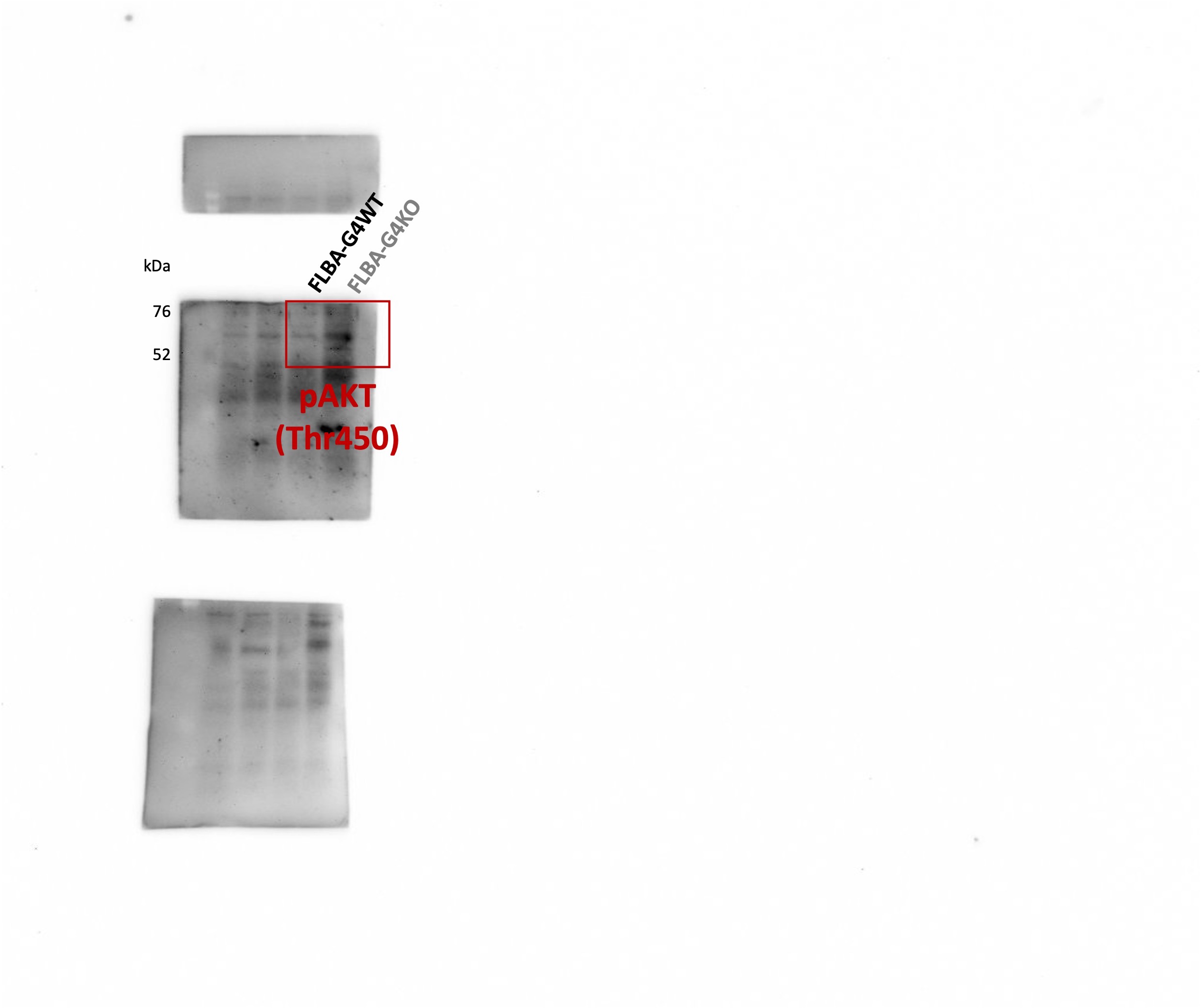

Supplement: Supplementary file 11 — Source Data for Figure 3 [file EMMM-15-e17570-s002.zip › Data source Figure 3/Western blot Figure 3E/Fig3E pAKT (Thr450).jpg]

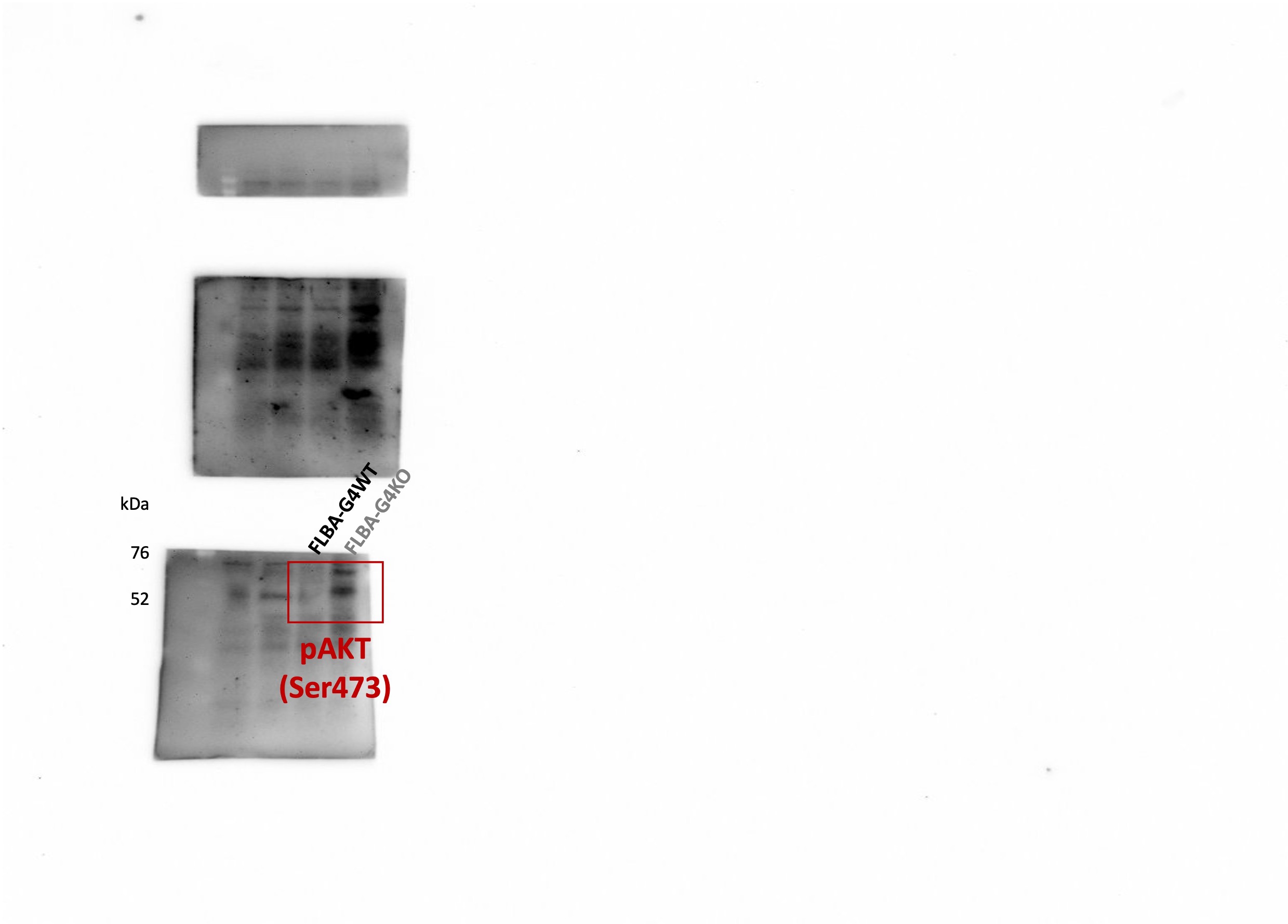

Supplement: Supplementary file 11 — Source Data for Figure 3 [file EMMM-15-e17570-s002.zip › Data source Figure 3/Western blot Figure 3E/Fig3E pAKT (Ser473).jpg]

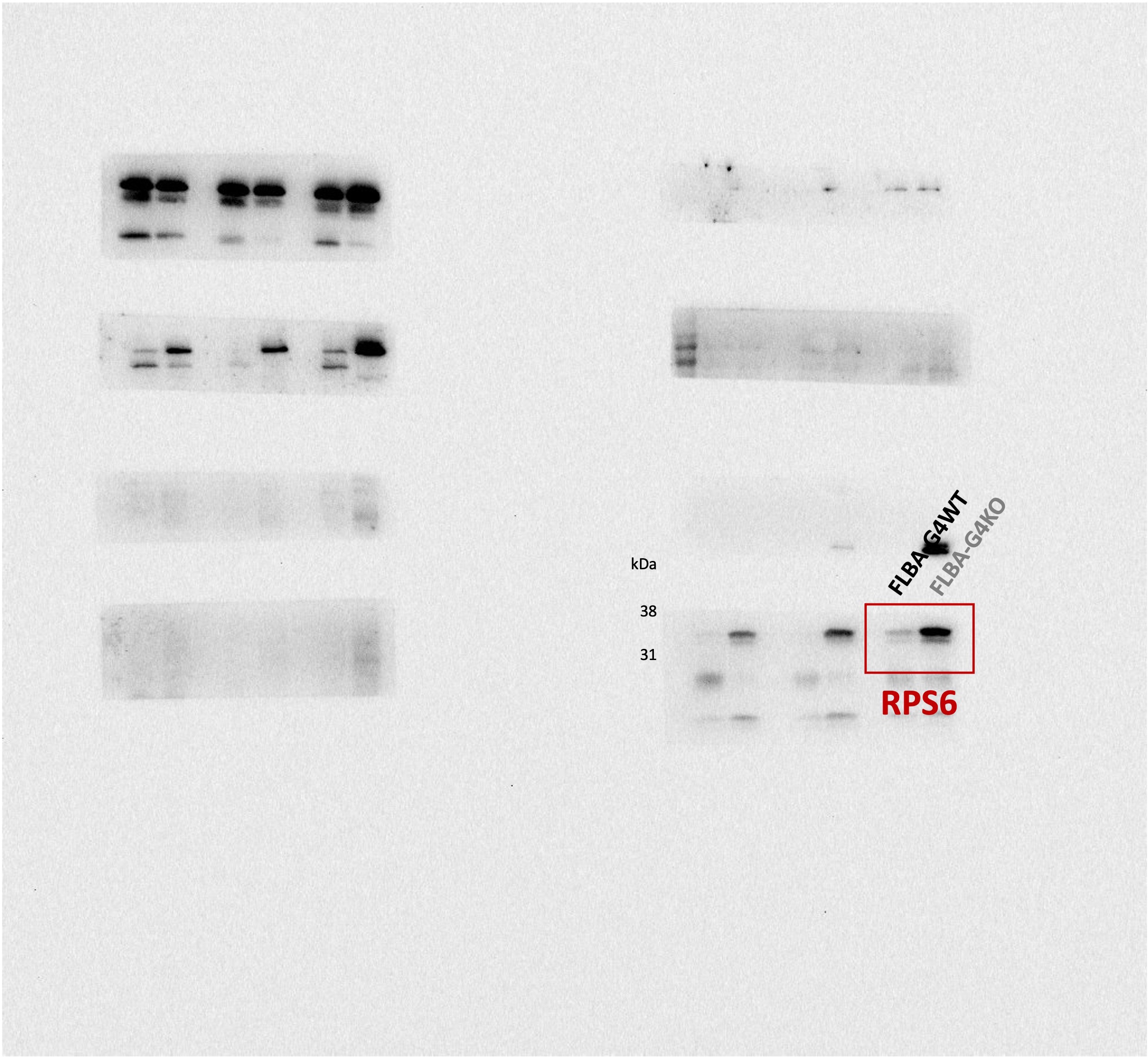

Supplement: Supplementary file 11 — Source Data for Figure 3 [file EMMM-15-e17570-s002.zip › Data source Figure 3/Western blot Figure 3E/Fig3E RPS6.jpg]

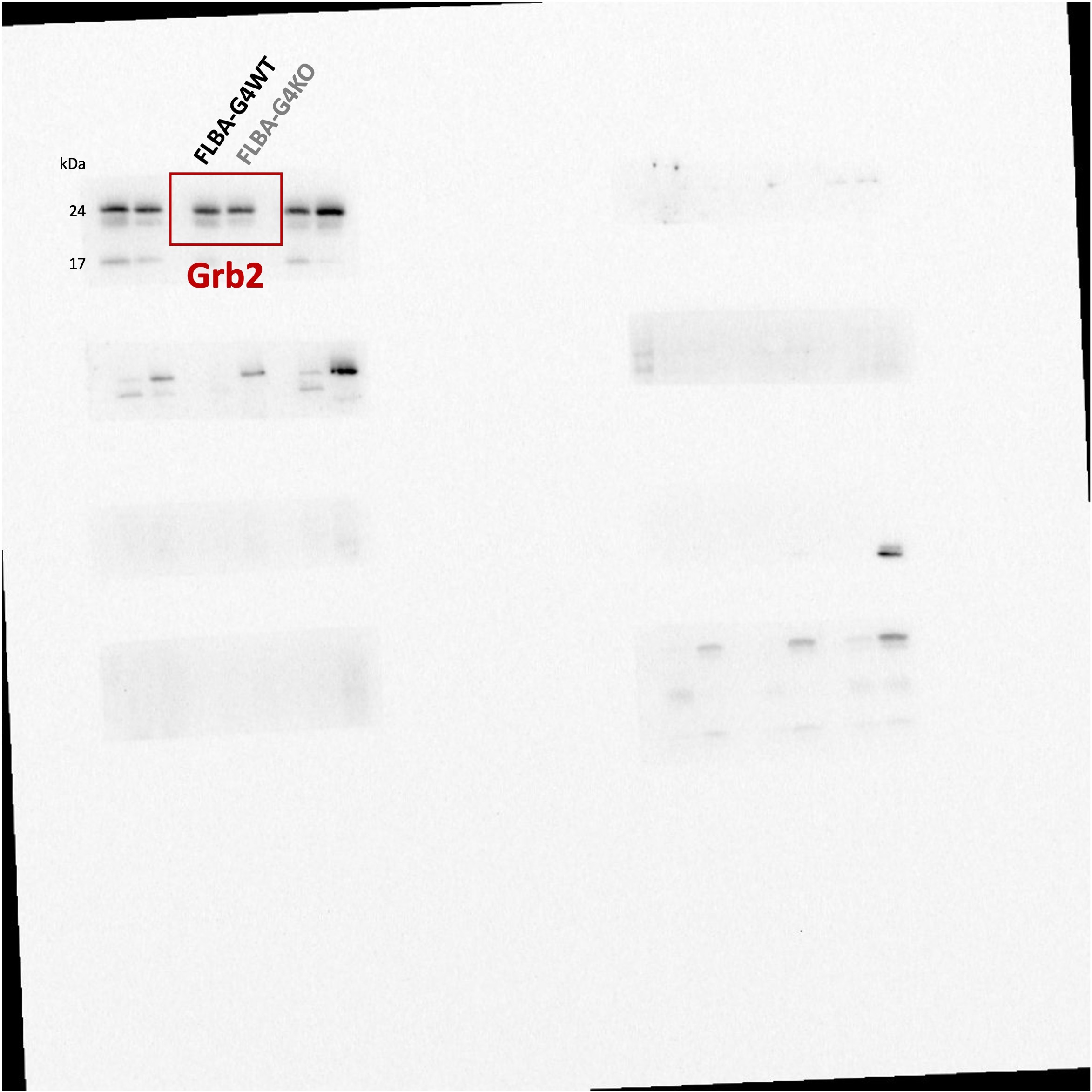

Supplement: Supplementary file 11 — Source Data for Figure 3 [file EMMM-15-e17570-s002.zip › Data source Figure 3/Western blot Figure 3E/Fig3E Grb2.jpg]

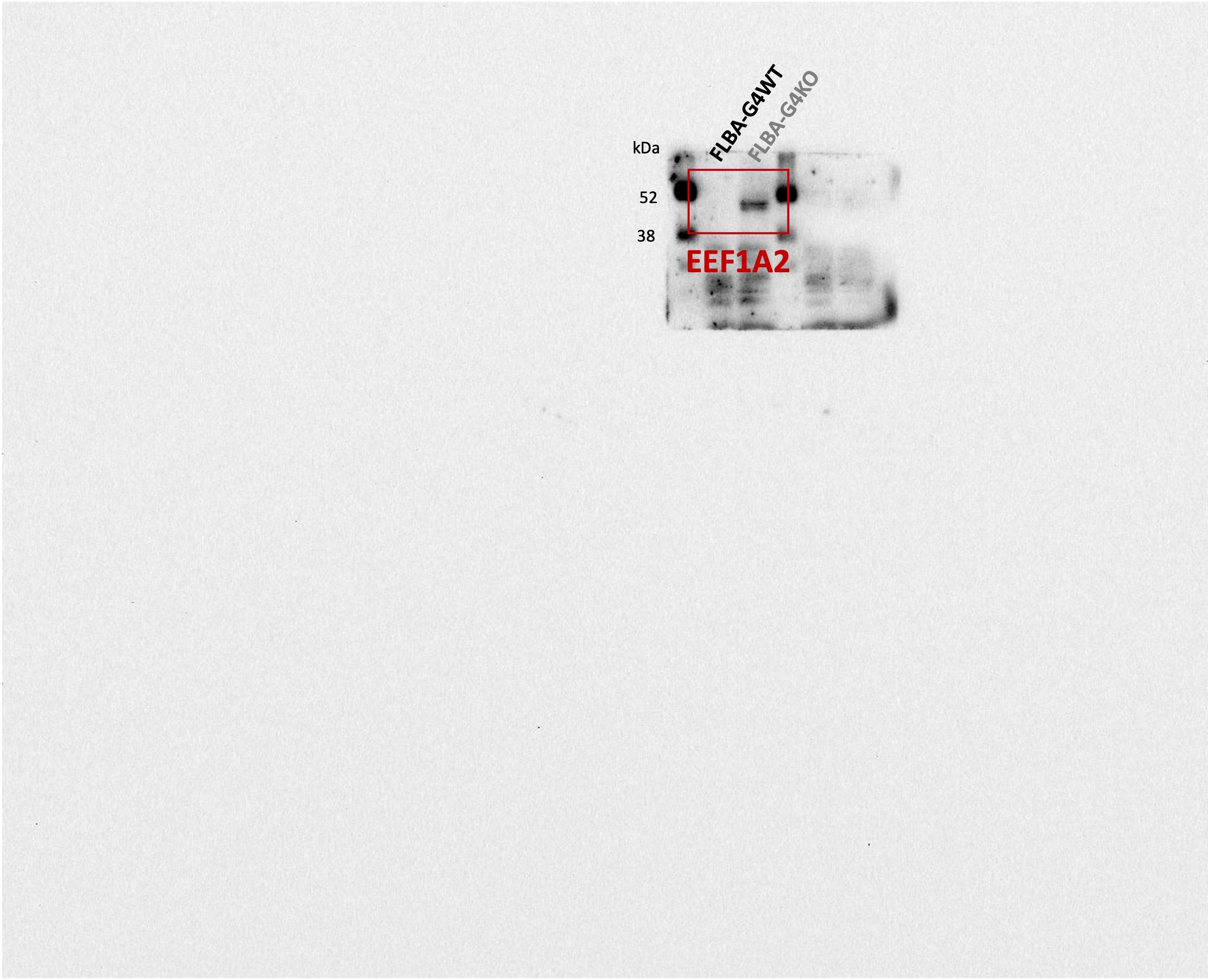

Supplement: Supplementary file 11 — Source Data for Figure 3 [file EMMM-15-e17570-s002.zip › Data source Figure 3/Western blot Figure 3F/Fig3F EEF1A2.jpg]

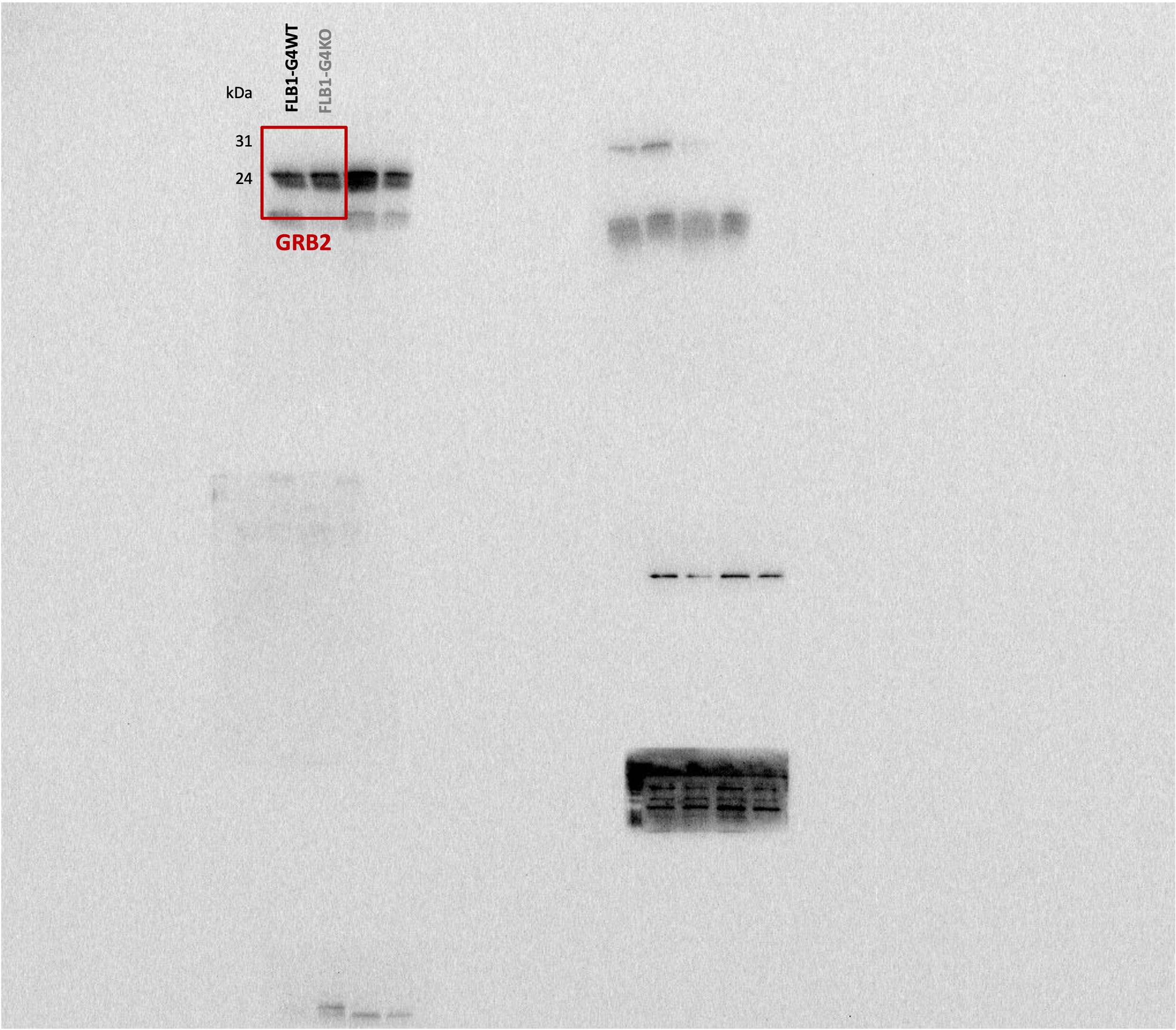

Supplement: Supplementary file 11 — Source Data for Figure 3 [file EMMM-15-e17570-s002.zip › Data source Figure 3/Western blot Figure 3F/Fig3F Grb2.jpg]

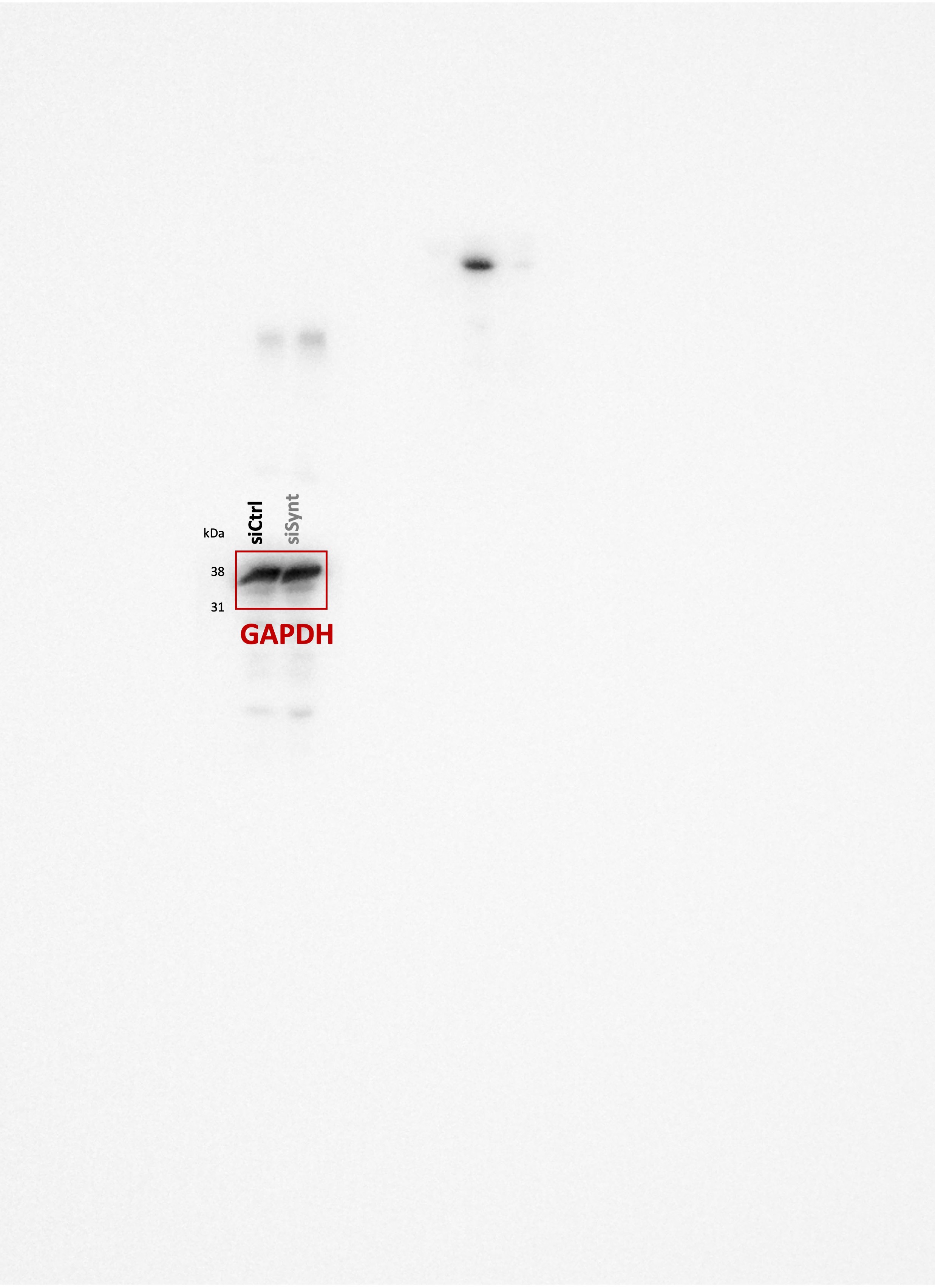

Supplement: Supplementary file 13 — Source Data for Figure 5 [file EMMM-15-e17570-s003.zip › Data source Figure 5/Western blot Figure 5C/Fig5C HS27a cell lysate - GAPDH.jpg]

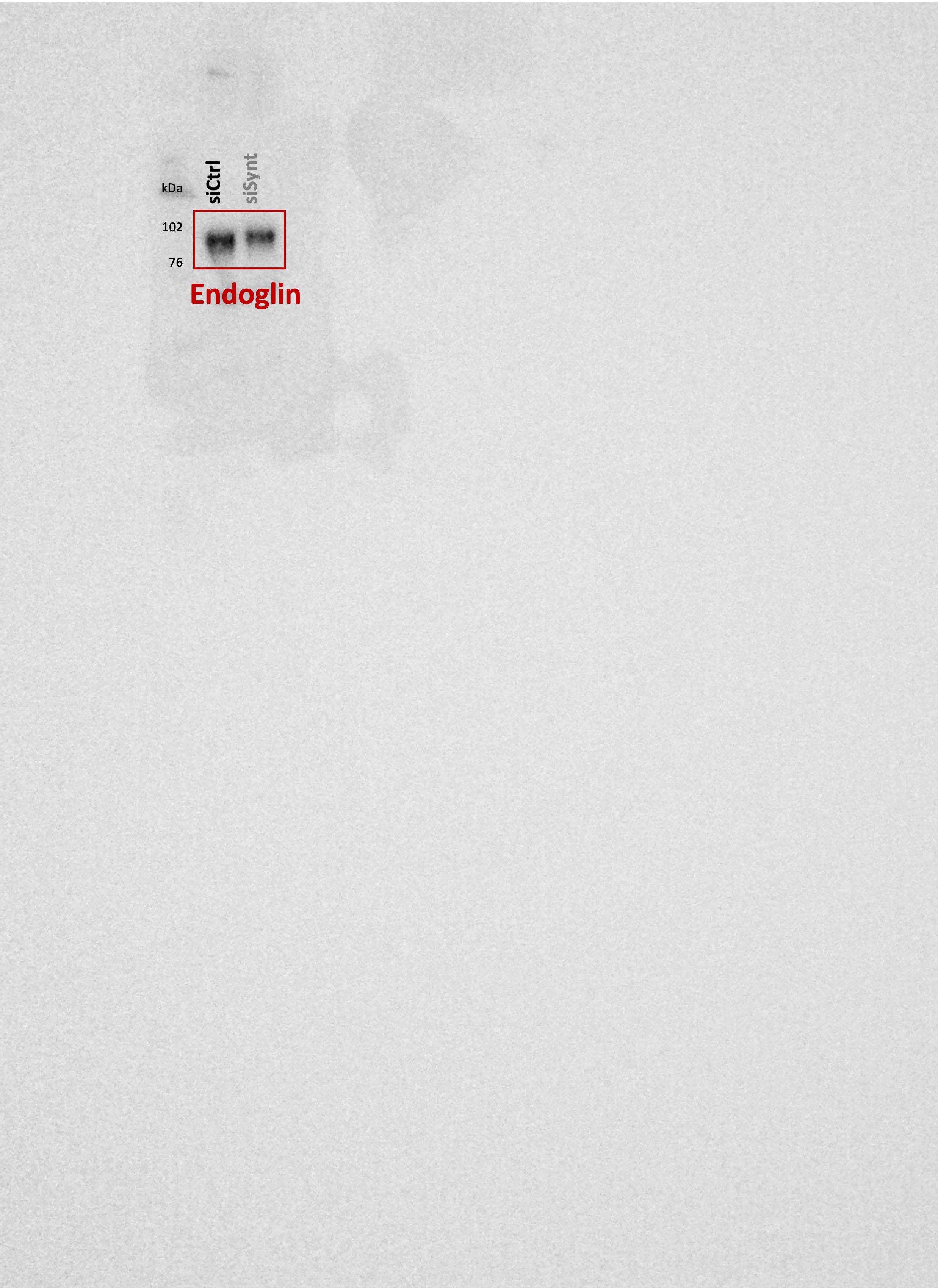

Supplement: Supplementary file 13 — Source Data for Figure 5 [file EMMM-15-e17570-s003.zip › Data source Figure 5/Western blot Figure 5C/Fig5C HS27a smallEVs - Endoglin.jpg]

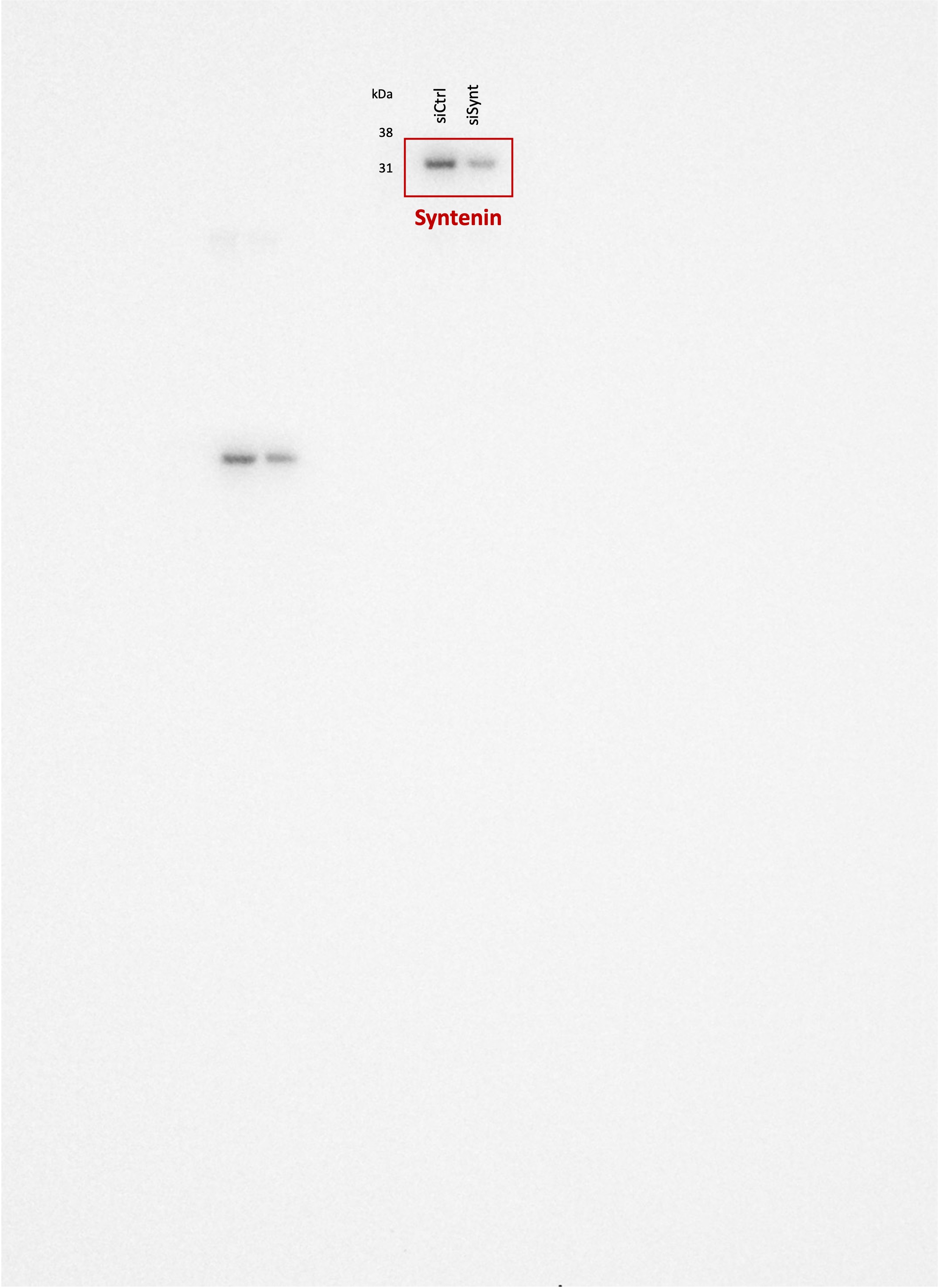

Supplement: Supplementary file 13 — Source Data for Figure 5 [file EMMM-15-e17570-s003.zip › Data source Figure 5/Western blot Figure 5C/Fig5C HS27a smallEVs - Syntenin.jpg]

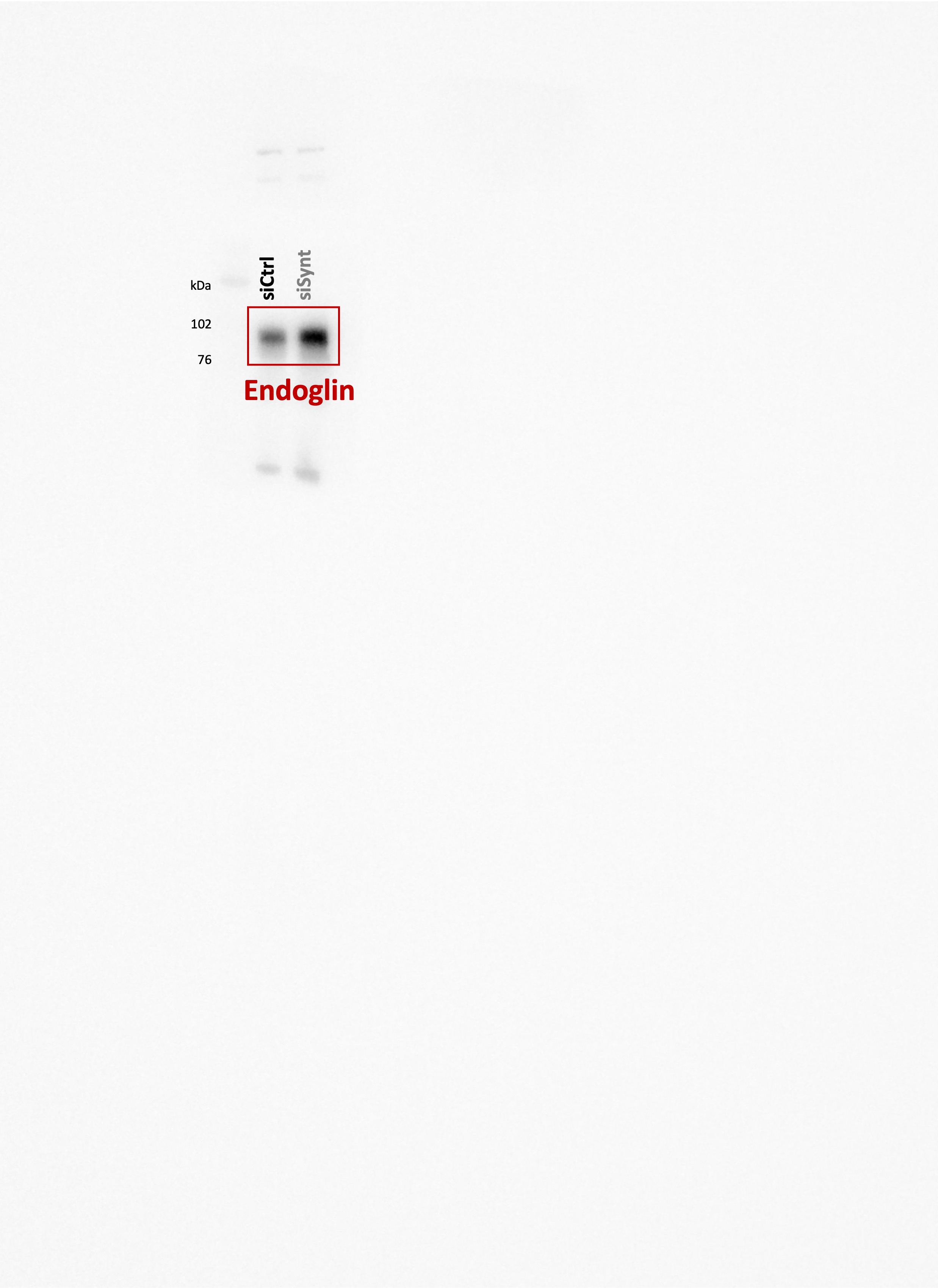

Supplement: Supplementary file 13 — Source Data for Figure 5 [file EMMM-15-e17570-s003.zip › Data source Figure 5/Western blot Figure 5C/Fig5C HS27a cell lysate - Endoglin.jpg]

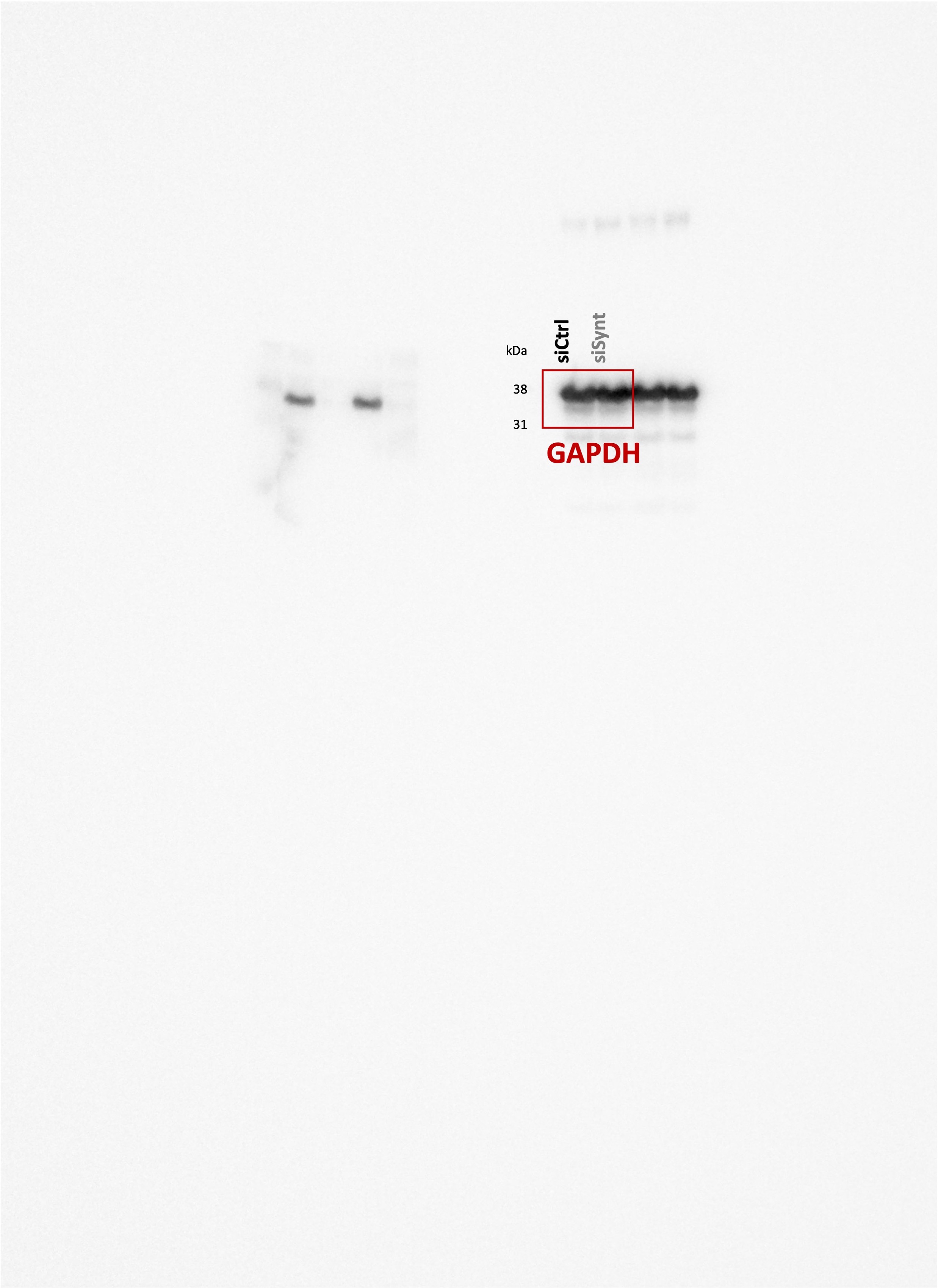

Supplement: Supplementary file 13 — Source Data for Figure 5 [file EMMM-15-e17570-s003.zip › Data source Figure 5/Western blot Figure 5C/Fig5C HS5 cell lysate - GAPDH.jpg]

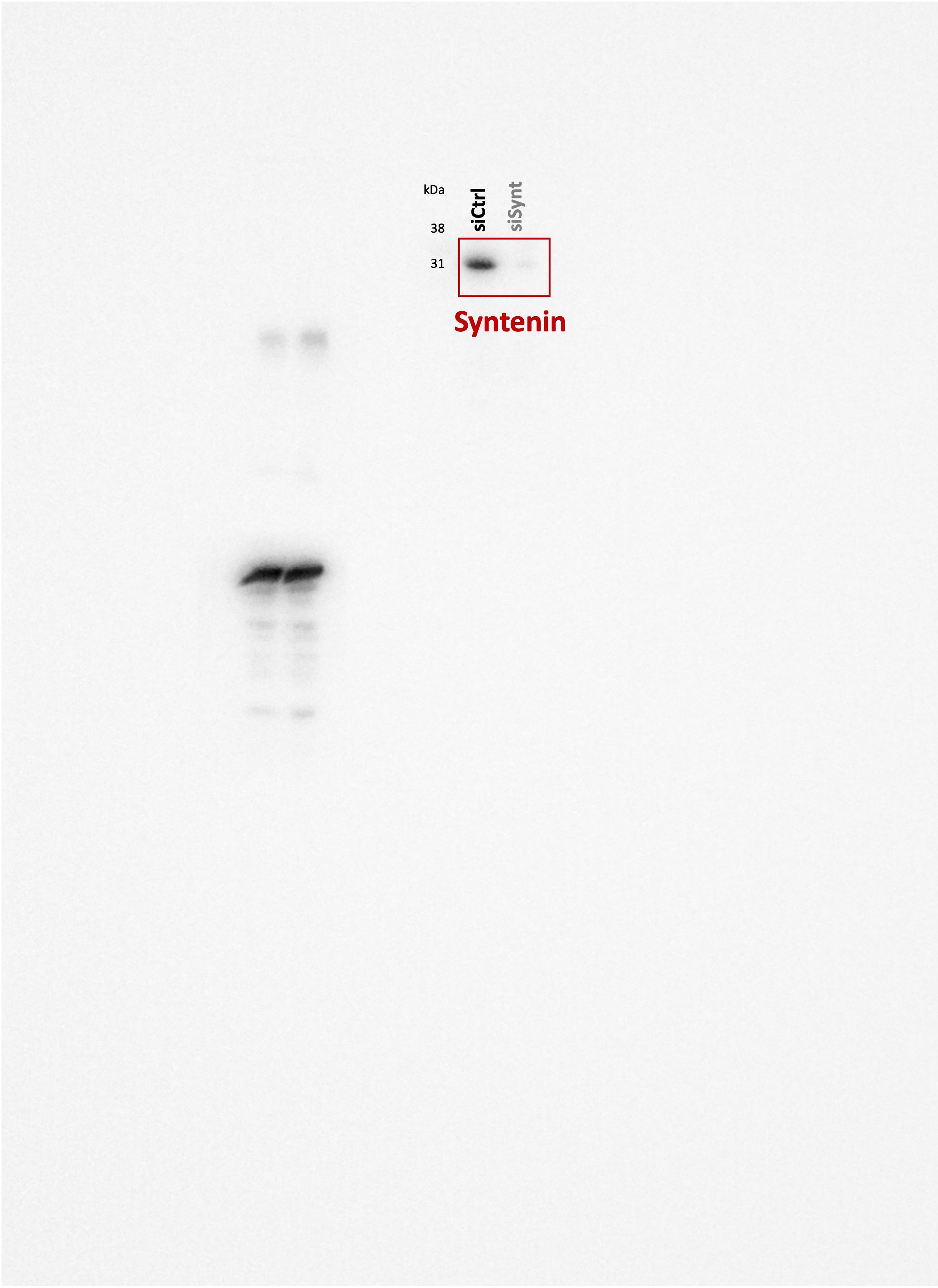

Supplement: Supplementary file 13 — Source Data for Figure 5 [file EMMM-15-e17570-s003.zip › Data source Figure 5/Western blot Figure 5C/Fig5C HS27a cell lysate - syntenin.jpg]

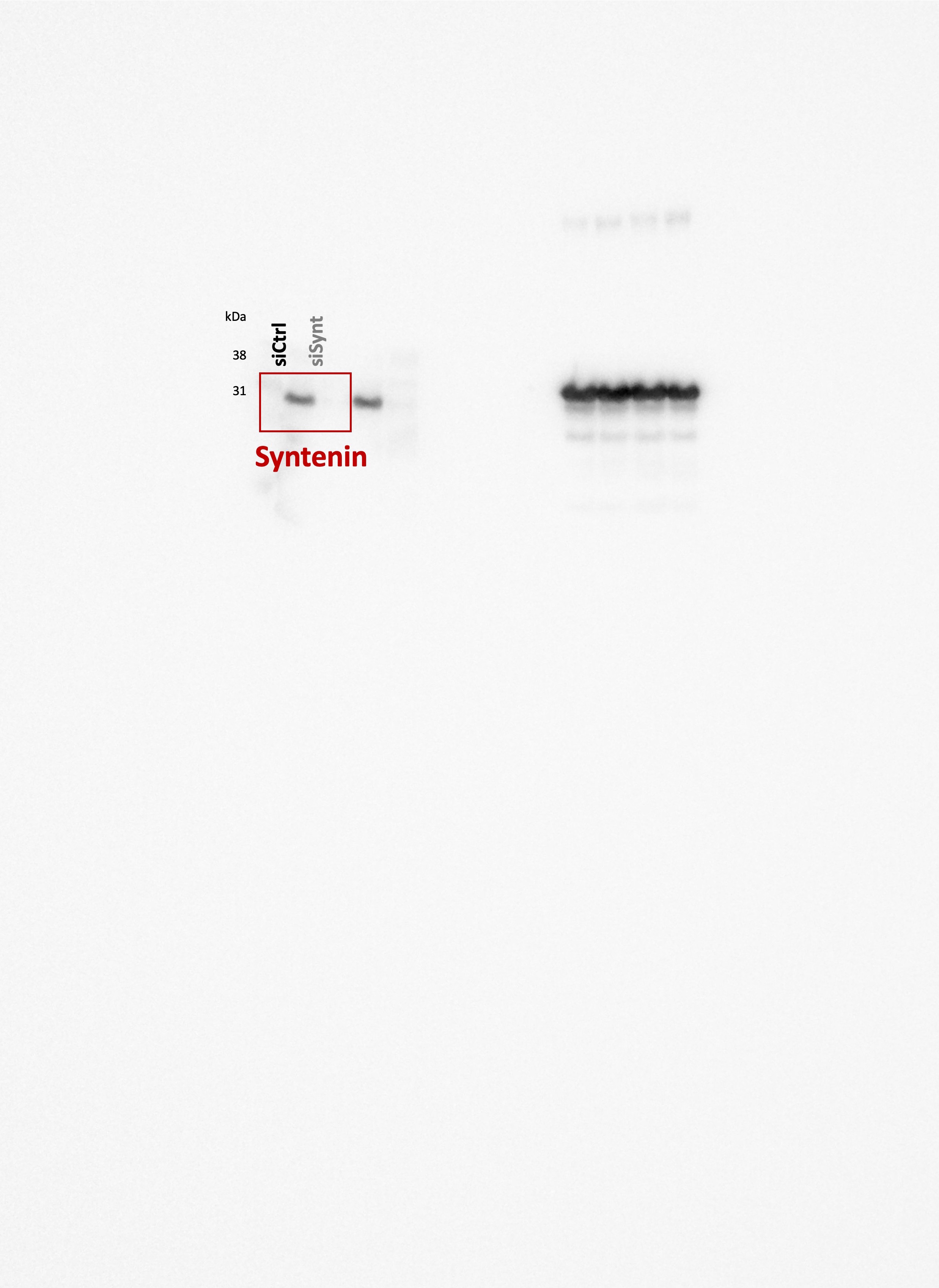

Supplement: Supplementary file 13 — Source Data for Figure 5 [file EMMM-15-e17570-s003.zip › Data source Figure 5/Western blot Figure 5C/Fig5C HS5 cell lysate - syntenin.jpg]

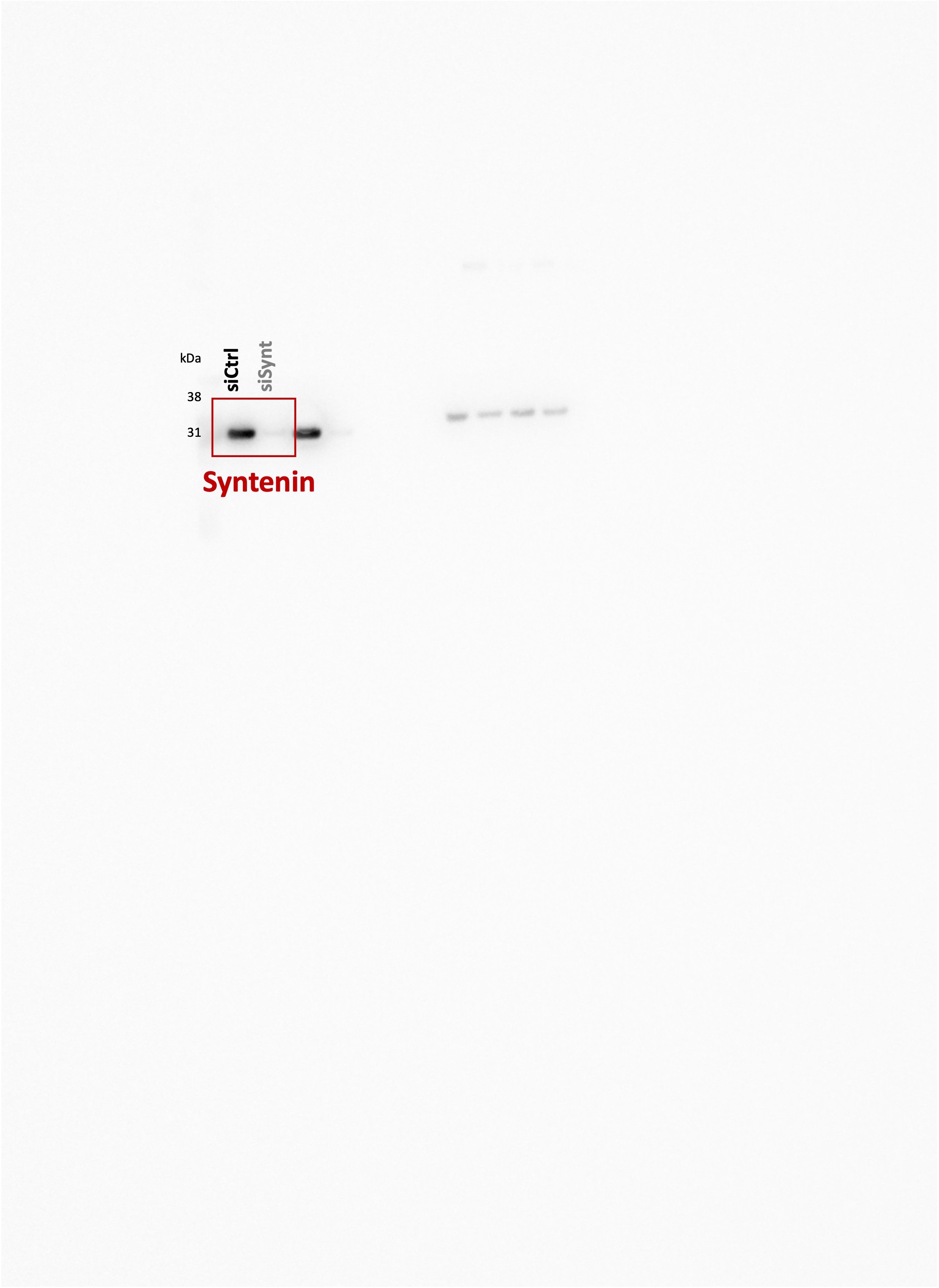

Supplement: Supplementary file 13 — Source Data for Figure 5 [file EMMM-15-e17570-s003.zip › Data source Figure 5/Western blot Figure 5C/Fig5C HS5 smallEVs - syntenin.jpg]

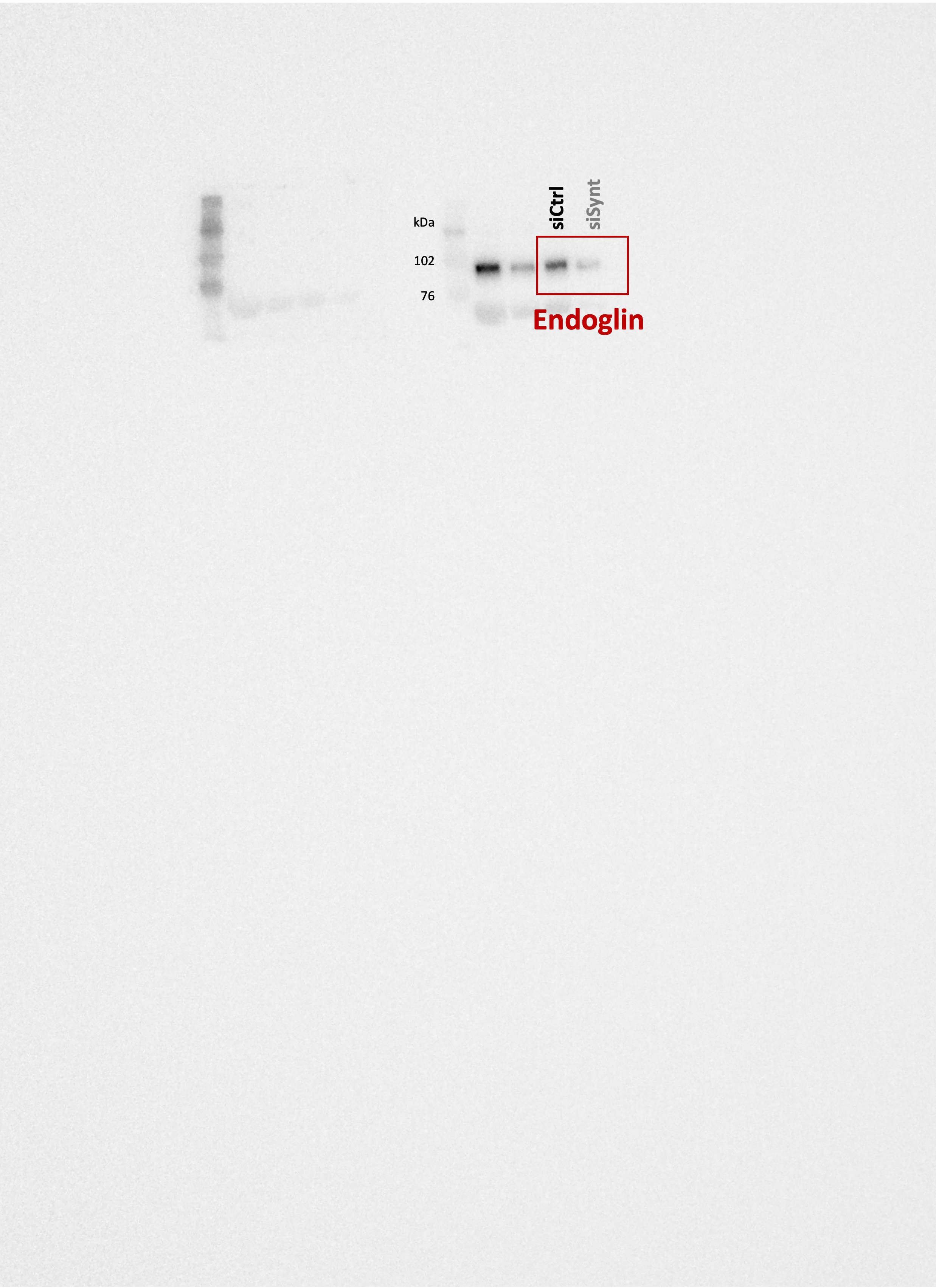

Supplement: Supplementary file 13 — Source Data for Figure 5 [file EMMM-15-e17570-s003.zip › Data source Figure 5/Western blot Figure 5C/Fig5C HS5 smallEVs - Endoglin.jpg]

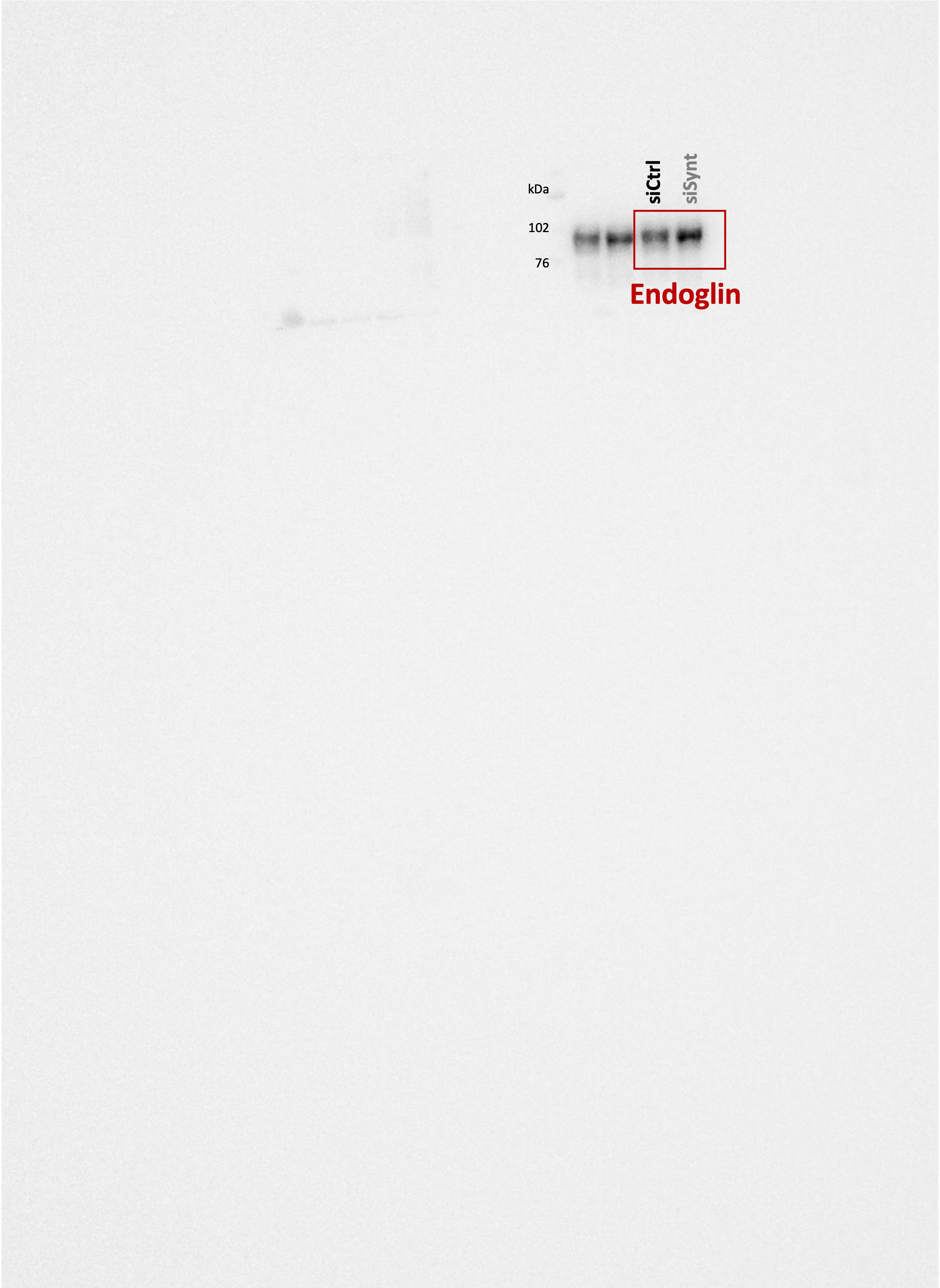

Supplement: Supplementary file 13 — Source Data for Figure 5 [file EMMM-15-e17570-s003.zip › Data source Figure 5/Western blot Figure 5C/Fig5C HS5 cell lysate - Endoglin.jpg]

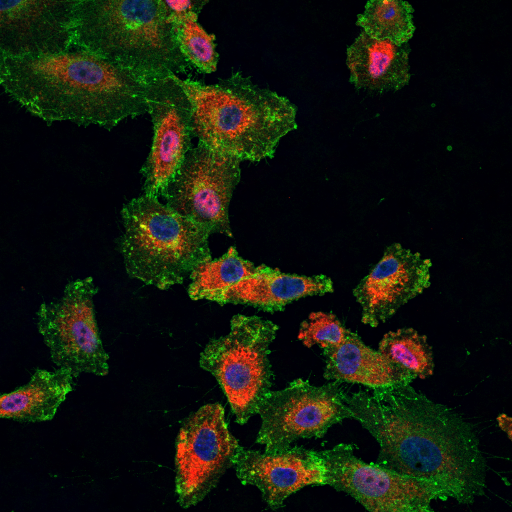

Supplement: Supplementary file 13 — Source Data for Figure 5 [file EMMM-15-e17570-s003.zip › Data source Figure 5/Confocal microscopy Figure 5E/Figure 5E/HS5 cells.tif]

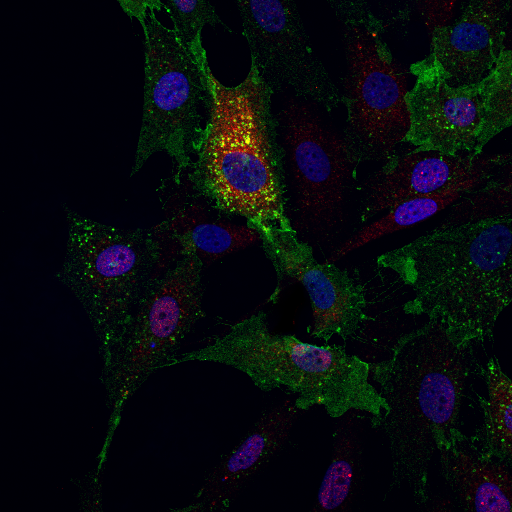

Supplement: Supplementary file 13 — Source Data for Figure 5 [file EMMM-15-e17570-s003.zip › Data source Figure 5/Confocal microscopy Figure 5E/Figure 5E/HS27a cells.tif]
