# Supplementary material for: Downregulation of stromal syntenin sustains AML development
Source: EMBO Mol Med. 2023 Oct 11;15(11):e17570. doi: 10.15252/emmm.202317570 (PMC10630886; doi:10.15252/emmm.202317570)
Supplement: Supplementary file 15 [file EMMM-15-e17570-s012.pdf]

EMBO Molecular Medicine

Downregulation of stromal syntenin sustains AML development

Raphael Leblanc, Rania Ghossoub, Armelle Goubard, Rémy Castellano, Joanna Fares, Luc Camoin, Stephane Audebert, Marielle Balzano, Berna Bou-Tayeh, Cyril Fauriat, Norbert Vey, Sylvain Garciaz, Jean-Paul Borg, yves collette, Michel Aurrand-Lions, Guido David, and Pascale Zimmermann
DOI: 10.15252/emmm.202317570

Corresponding authors: Raphael Leblanc (raphael.leblanc@inserm.fr) , Pascale Zimmermann (pascale.zimmermann@inserm.fr)

Review Timeline:

Submission Date:	15th Feb 23
Editorial Decision:	6th Mar 23
Revision Received:	30th Jul 23
Editorial Decision:	30th Aug 23
Revision Received:	8th Sep 23
Editorial Decision:	11th Sep 23
Revision Received:	13th Sep 23
Accepted:	14th Sep 23

Editor: Lise Roth

Transaction Report:

(Note: With the exception of the correction of typographical or spelling errors that could be a source of ambiguity, letters and reports are not edited. Depending on transfer agreements, referee reports obtained elsewhere may or may not be included in this compilation. Referee reports are anonymous unless the Referee chooses to sign their reports.)

6th Mar 2023

Dear Dr. Leblanc,

Thank you for the submission of your manuscript to EMBO Molecular Medicine. We have now received feedback from the three reviewers who agreed to evaluate your manuscript. As you will see from the reports below, the referees acknowledge the interest of the study and are overall supporting publication of your work pending appropriate revisions.

Addressing the reviewers' concerns in full will be necessary for further considering the manuscript in our journal, and acceptance of the manuscript will entail a second round of review. EMBO Molecular Medicine encourages a single round of revision only and therefore, acceptance or rejection of the manuscript will depend on the completeness of your responses included in the next, final version of the manuscript. For this reason, and to save you from any frustrations in the end, I would strongly advise against returning an incomplete revision.

If you would like to discuss further the points raised by the referees, I am available to do so via email or video. Let me know if you are interested in this option.

Revised manuscripts should be submitted within three months of a request for revision; they will otherwise be treated as new submissions, except under exceptional circumstances in which a short extension is obtained from the editor.

When submitting your revised manuscript, please carefully review the instructions that follow below. We perform an initial quality control of all revised manuscripts before re-review; failure to include requested items will delay the evaluation of your revision.

We require:

1) A .docx formatted version of the manuscript text (including legends for main figures, EV figures and tables). Please make sure that the changes are highlighted to be clearly visible.

2) Individual production quality figure files as .eps, .tif, .jpg (one file per figure). For guidance, download the 'Figure Guide PDF' (https://www.embopress.org/page/journal/17574684/authorguide#figureformat).

3) At EMBO Press we ask authors to provide source data for the main figures. Our source data coordinator will contact you to discuss which figure panels we would need source data for and will also provide you with helpful tips on how to upload and organize the files.

4) A .docx formatted letter INCLUDING the reviewers' reports and your detailed point-by-point responses to their comments. As part of the EMBO Press transparent editorial process, the point-by-point response is part of the Review Process File (RPF), which will be published alongside your paper.

5) A complete author checklist, which you can download from our author guidelines (https://www.embopress.org/page/journal/17574684/authorguide#submissionofrevisions). Please insert information in the checklist that is also reflected in the manuscript. The completed author checklist will also be part of the RPF.

6) Please note that all corresponding authors are required to supply an ORCID ID for their name upon submission of a revised manuscript. An ORCID identified is currently missing for Prof. Pascale Zimmermann.

7) It is mandatory to include a 'Data Availability' section after the Materials and Methods. Before submitting your revision, primary datasets produced in this study need to be deposited in an appropriate public database, and the accession numbers and database listed under 'Data Availability'. Please remember to provide a reviewer password if the datasets are not yet public (see https://www.embopress.org/page/journal/17574684/authorguide#dataavailability).

In case you have no data that requires deposition in a public database, please state so in this section "This study includes no data deposited in external repositories". Note that the Data Availability Section is restricted to new primary data that are part of this study.

8) For data quantification: please specify the name of the statistical test used to generate error bars and P values, the number (n) of independent experiments (specify technical or biological replicates) underlying each data point and the test used to calculate p-values in each figure legend. The figure legends should contain a basic description of n, P and the test applied. Graphs must include a description of the bars and the error bars (s.d., s.e.m.). Please provide exact p values.

9) Our journal encourages inclusion of *data citations in the reference list* to directly cite datasets that were re-used and obtained from public databases. Data citations in the article text are distinct from normal bibliographical citations and should

directly link to the database records from which the data can be accessed. In the main text, data citations are formatted as follows: "Data ref: Smith et al, 2001" or "Data ref: NCBI Sequence Read Archive PRJNA342805, 2017". In the Reference list, data citations must be labeled with "[DATASET]". A data reference must provide the database name, accession number/identifiers and a resolvable link to the landing page from which the data can be accessed at the end of the reference. Further instructions are available at .

10) We replaced Supplementary Information with Expanded View (EV) Figures and Tables that are collapsible/expandable online. A maximum of 5 EV Figures can be typeset. EV Figures should be cited as 'Figure EV1, Figure EV2" etc... in the text and their respective legends should be included in the main text after the legends of regular figures.
- For the figures that you do NOT wish to display as Expanded View figures, they should be bundled together with their legends in a single PDF file called *Appendix*, which should start with a short Table of Content. Appendix figures should be referred to in the main text as: "Appendix Figure S1, Appendix Figure S2" etc.
- Additional Tables/Datasets should be labeled and referred to as Table EV1, Dataset EV1, etc. Legends have to be provided in a separate tab in case of .xls files. Alternatively, the legend can be supplied as a separate text file (README) and zipped together with the Table/Dataset file.
See detailed instructions here:

11) The paper explained: EMBO Molecular Medicine articles are accompanied by a summary of the articles to emphasize the major findings in the paper and their medical implications for the non-specialist reader. Please provide a draft summary of your article highlighting
- the medical issue you are addressing,
- the results obtained and
- their clinical impact.
This may be edited to ensure that readers understand the significance and context of the research. Please refer to any of our published articles for an example.

12) For more information: There is space at the end of each article to list relevant web links for further consultation by our readers. Could you identify some relevant ones and provide such information as well? Some examples are patient associations, relevant databases, OMIM/proteins/genes links, author's websites, etc...

13) Author contributions: CRediT has replaced the traditional author contributions section because it offers a systematic machine readable author contributions format that allows for more effective research assessment. Please remove the Authors Contributions from the manuscript and use the free text boxes beneath each contributing author's name in our system to add specific details on the author's contribution. More information is available in our guide to authors.
14) Disclosure statement and competing interests: We updated our journal's competing interests policy in January 2022 and request authors to consider both actual and perceived competing interests. Please review the policy https://www.embopress.org/competing-interests and update your competing interests if necessary.

15) Every published paper now includes a 'Synopsis' to further enhance discoverability. Synopses are displayed on the journal webpage and are freely accessible to all readers. They include a short stand first (maximum of 300 characters, including space) as well as 2-5 one-sentences bullet points that summarizes the paper. Please write the bullet points to summarize the key NEW findings. They should be designed to be complementary to the abstract - i.e. not repeat the same text. We encourage inclusion of key acronyms and quantitative information (maximum of 30 words / bullet point). Please use the passive voice. Please attach these in a separate file or send them by email, we will incorporate them accordingly.

Please also suggest a striking image or visual abstract to illustrate your article as a PNG file 550 px wide x 300-600 px high.

16) As part of the EMBO Publications transparent editorial process initiative (see our Editorial at http://embomolmed.embopress.org/content/2/9/329), EMBO Molecular Medicine will publish online a Review Process File (RPF) to accompany accepted manuscripts.
In the event of acceptance, this file will be published in conjunction with your paper and will include the anonymous referee reports, your point-by-point response and all pertinent correspondence relating to the manuscript. Let us know whether you agree with the publication of the RPF and as here, if you want to remove or not any figures from it prior to publication.
Please note that the Authors checklist will be published at the end of the RPF.

EMBO Molecular Medicine has a "scooping protection" policy, whereby similar findings that are published by others during review or revision are not a criterion for rejection. Should you decide to submit a revised version, I do ask that you get in touch after three months if you have not completed it, to update us on the status.

I look forward to receiving your revised manuscript.

Yours sincerely,

Lise Roth

Lise Roth, PhD
Senior Editor
EMBO Molecular Medicine

EMBO Press participates in many Publish and Read agreements that allow authors to publish Open Access with reduced/no publication charges. Check your eligibility: https://authorservices.wiley.com/author-resources/Journal-Authors/open-access/affiliation-policies-payments/index.html

***** Reviewer's comments *****

Referee #1 (Comments on Novelty/Model System for Author):

The use of mouse models to test the author´s hypothesis is adequate. The use of sufficient animals to achieve statistical significance is justified.

Referee #1 (Remarks for Author):

This is an interesting and relevant study on the mechanisms by which the bone marrow stroma may impact the development and onset of acute myeloid leukemia.
The data shows that syntenin downregulation promotes acute leukemia expansion in vivo (in several mouse models) and in vivo.
The authors use different cell lines and approaches to reach a somewhat complicated conclusion that syntenin downregulation promotes endoglin secretion which in turn promotes acute myeloid leukemia expansion.
This broad reaching conclusion is partially supported by the data, however, I have several (mostly conceptual and related to the authors intrepretation) comments and concerns:

- endoglin expression by subsets of leukemia cells has been reported, and the conclusion of those studies point to an important role of the bone marrow vasculature in promoting leukemia (AML) expansion. Have the authors of the present manuscript investigated the importance of bone marrow angiogenesis (or -if it is not a quantative byt rather a qualitative change in endothelial behavior and content- the angiocrine profile) of syntenin deficient bone marrows? What is the role of endothelial cells in this setting?
- the mechanism by which syntenin acts is somewhat confusing: if it is simply resulting in downregulation of endoglin secretion (not production?), this should be clearly stated in the manuscript.
- have the authors studied the expression of syntenin and its putative role in regulating endoglin availability in Human primary leukemia samples? How relevant are the authors claims in a real, clinically relevant setting?

minor comment: the quality of the WBs could be improved.

Referee #2 (Comments on Novelty/Model System for Author):

Overall, the experiments were designed in rational and data presented support major conclusions.

Referee #2 (Remarks for Author):

EMBO Molecular Medicine
Manuscript Number: EMM-2023-17570
"Downregulation of stromal syntenin sustains AML development" by Leblanc R. et al.
In this paper, authors investigated the downregulation of syntenin in Bone Marrow Stromal Cells (BNSC) by AML cells expressing high levels of miR-155 and the consequence on their progression in vitro and in vivo. By different approaches, they demonstrated that syntenin deficiency in BMSC sustains leukemic aggressiveness by promoting leukemic cell survival and protein synthesis. At molecular level, they identified a regulatory loop potentially implicated in the AML-stroma crosstalk which involves EEF1A2-AKT-RPS6 signaling pathway.

Overall, the experiments were designed in rational and data presented support major conclusions. However, there are some concerns that need to be addressed before consideration for publication in EMBO Molecular Medicine.

Major concerns:
Figure 2: FLB1 blasts gain in aggressiveness upon serial transplantation in a syntenin-deficient host.
Authors should perform these experiments with AML model expressing miR-155high and in these conditions evaluate syntenin expression in stromal cells.

Figure 5: Syntenin-deficiency in BMSC affects the distribution of endoglin.
5E. Authors should at least provide quantification of the confocal images. To better demonstrate the colocalization between endogenous endoglin and syntenin, they must perform proximity ligation assays.

Figure 6: High endoglin expression in BMSC acts in trans to regulate AML protein synthesis.

The mechanism(s) for how endoglin maintains AML cells proliferation and protein synthesis should be further elucidated
How to explain that when an invalidation for endoglin is carried out in panel 6B, the signal for pS473-AKT, pS235-RPS6 and EEF1A2 level increases whereas it was shown in panel A that under siRNA against endoglin the protein synthesis decreases.

Figure 7: Model recapitulating novel findings presented in this study.
Authors must provide more mechanistic demonstration to support their model.

Finally, did authors considered the impact of overexpression of syntenin on AML development?

Minor concerns:
Figure 2B: label on the axis indicates % of FLB1 cells in the peripheral blood at day 14 and contrasts with the title which indicates day15
In the text: Yet, FLB1 grown in syntenin-knockout mice significantly gained in aggressiveness from the 3rd transplantation on (Fig. 2B)

Referee #3 (Comments on Novelty/Model System for Author):

The data from human models are limited. More human cell lines should be included.

Referee #3 (Remarks for Author):

This work shows that loss of the PDZ-domain containing protein Syntenin in bone marrow stromal cells (BMSC) accelerates AML in experimental systems in vitro and in vivo. Investigation of gene expression datasets showed that experimentally induced AML models of different severity induce downregulation of Syntenin in the stromal compartment. This was correlated with expression levels of miR-155 in AML cells, and miR-155 downregulation reversed this effect. In transplantation experiments, loss of Syntenin in the host caused increased aggressiveness of transplanted murine leukemia cells in serial grafts. This was accompanied by activation of AKT, RPS6, elevated levels of Eef1a2 and increased protein synthesis. Similar observations were made in co-culture experiments of murine AML cells with wild-type vs. Syntenin-deficient BMSC as well as in co-cultures of the human AML cell line HL-60 with the HS5 stromal cell line in which Syntenin was silenced through RNA interference. Syntenin-deficient stromal cells exhibited increased surface expression of Endoglin, while Endoglin was depleted from small extracellular vesicles. Syntenin partially co-localized with Endoglin and downregulation of Endoglin in HS5 cells caused reduced protein synthesis in AML cells in co-cultures.

While this work provides evidence for the intriguing concept of stromal influence on AML pathophysiology, it has several shortcomings that compromise the relevance of the findings.

• Most importantly, the general relevance of the results is not convincingly demonstrated. Most experiments are performed in one murine leukemia model and one human AML cell line. Not all results were conserved in U937 cells. Why could this be the case? Several human AML cell lines and different stromal cell lines should be included to show the general relevance of the findings. Data on syntenin gene expression in human AML stroma should be included.

• Can knockdown of Eef1a2 or Metarrestin treatment normalize the competitive advantage of Syntenin-deficient cells?

• Is the Metarrestin effect specific to Syntenin-deficient cells? FLB1-G4WT cells should be shown for comparison in Figure S3B.

• Differentiation effects in Figure S4B should be quantified.

• In contrast to what is written in the text and what is shown in Figure 6B, the data in Figure 6C do not show that silencing of Endoglin has opposing effects to silencing of Syntenin, as knockdowns of both genes caused increased AKT/RPS6 phosphorylation and EEF1A2 expression. The respective section in the results section should be rephrased.

REVIEWERS' COMMENTS TO AUTHORS

REFEREE #1

This is an interesting and relevant study on the mechanisms by which the bone marrow stroma may impact the development and onset of acute myeloid leukemia.

The data shows that syntenin downregulation promotes acute leukemia expansion in vivo (in several mouse models) and in vivo. The authors use different cell lines and approaches to reach a somewhat complicated conclusion that syntenin downregulation promotes endoglin secretion which in turn promotes acute myeloid leukemia expansion.

This broad reaching conclusion is partially supported by the data, however, I have several (mostly conceptual and related to the authors intrepretation) comments and concerns:

Q1. Endoglin expression by subsets of leukemia cells has been reported, and the conclusion of those studies point to an important role of the bone marrow vasculature in promoting leukemia (AML) expansion. Have the authors of the present manuscript investigated the importance of bone marrow angiogenesis (or -if it is not a quantative by rather a qualitative change in endothelial behavior and content- the angiocrine profile) of syntenin deficient bone marrows? What is the role of endothelial cells in this setting?

R1. Indeed, in addition to being a marker for BMSCs and CAFs, endoglin plays an essential role in angiogenesis, particularly in tumor angiogenesis. Specifically, in that regard, we performed immunohistochemistry to test for a possible vascular phenotype in our Synt-KO animals. As shown below, we did not observe vasculature defects in our syntenin KO mice (CD31 staining). Consequently, we did not investigate further in that direction. We decided not to introduce these observations in the current manuscript to avoid compromising the flow of information.

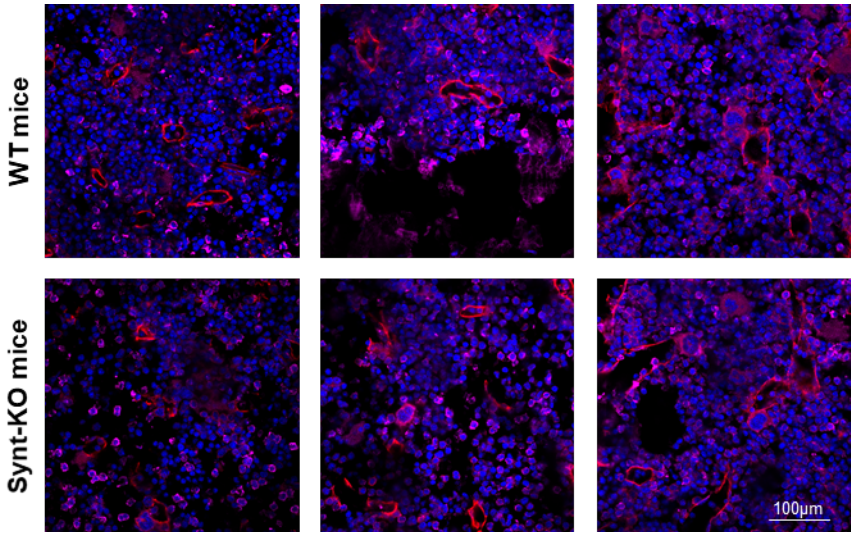

__Figure in support of R1 (for referee only).__ Immunostaining of bone marrow sections from C57BL/6J WT or Synt-KO mice. The images represent bone marrow sections from 3 different animals per group. The vasculature is stained using a CD31 antibody (Red staining), osteoblasts and nucleus are respectively stained using an osteocalcin antibody (Purple staining), and DAPI is used for nuclear staining (Blue staining).

Q2. The mechanism by which syntenin acts is somewhat confusing: if it is simply resulting in downregulation of endoglin secretion (not production?), this should be clearly stated in the manuscript.

R2. We now assessed endoglin RNA levels in both HS5 and HS27a stromal cells transfected with syntenin-directed siRNA or control. As shown in **figure EV1B**, we found that syntenin deficiency does affect the mRNA level of endoglin in stromal cells. These data illustrate that the primary function of syntenin in bone marrow stromal cells is not related to changes in production. We also add evidence that syntenin does not affect the stability of endoglin protein (cycloheximide experiments) (**Appendix**

Fig. S5A & B). Of note, an extensive body of prior evidence (in large part from our own work, cited in the manuscript) indicates syntenin plays an essential role in the regulation of the endosomal trafficking of the cargo that binds to its PDZ domains. Our data suggest that it includes the regulation of endoglin trafficking in BMSC, syntenin-deficiency leading to an increased level of endoglin at BMSC cell surfaces (**Fig. 5A-C & EV1A**.), associated with a decreased release of endoglin via BMSC-exosomes (**Fig. 5C**). Thus, endoglin adds to the list of important syntenin-controlled cell surface receptors and co-receptors. While the specific details of this syntenin-regulated endoglin traffic in BMSC deserve to be worked out, such will not affect the conclusions. Altogether, our results suggest that syntenin acts on the protein turnover of endoglin in the stromal cell (its entry in 'exit' routes) but does not affect the production of endoglin. We hope this is clear now from the data, text and discussion.

Q3. Have the authors studied the expression of syntenin and its putative role in regulating endoglin availability in Human primary leukemia samples? How relevant are the authors claims in a real, clinically relevant setting?

R3. To date very few clinical data exploring AML-BMSC have been generated. Indeed, BMSCs constitute a very limited population within the BM (and a fortiori the AML-BM). Available databases do not reveal any dysregulation of syntenin expression within expanded AML-BMSCs compared to healthy donor-BMSC, but data were obtained from limited number of patients without any specifications (Von Der Heide et al. 2017). Although not currently applicable to routine work, new single cell omics approaches might allow setting up of a pipeline to analyze direct expression of syntenin (or that of agents, e.g. miRs that can downregulate syntenin) in BMSCs. Detecting the decrease of a syntenin transcript that normally is not abundant to start with, also presents a particular challenge. To address this issue indirectly and to so respond to this referee request, we tested whether aggressive forms of AML can upregulate the expression of endoglin at the surface of BMSCs in patients. Briefly, bone marrow samples isolated from AML FLT3-WT vs AML-FLT3-ITD patients were negatively selected for CD3, CD14, CD41, CD19, CD235a, CD45 & CD31. We then measured the membrane expression of endoglin in this population of 'stromal' cells (the term 'stromal' taken in the broad sense), when at least 300 stromal cells were reliably detected (see details in **Dataset EV4**). As shown in **Figure 6A** and in **Dataset EV4**, our results reveal that aggressive AML forms carrying the FLT3-ITD mutation significantly up-regulate endoglin expression on the surface of stromal cells. Although the patient cohort is limited (17 patients FLT3-WT vs. 8 patients FLT3-ITD), this result strongly suggests the clinical relevance of our findings in AML.

REFEREE #2

In this paper, authors investigated the downregulation of syntenin in Bone Marrow Stromal Cells (BNSC) by AML cells expressing high levels of miR-155 and the consequence on their progression in vitro and in vivo. By different approaches, they demonstrated that syntenin deficiency in BMSC sustains leukemic aggressiveness by promoting leukemic cell survival and protein synthesis. At molecular level, they identified a regulatory loop potentially implicated in the AML-stroma crosstalk which involves EEF1A2-AKT-RPS6 signaling pathway.

Overall, the experiments were designed in rational and data presented support major conclusions. However, there are some concerns that need to be addressed before consideration for publication in EMBO Molecular Medicine.

Q1. Figure 2: FLB1 blasts gain in aggressiveness upon serial transplantation in a syntenin-deficient host. Authors should perform these experiments with AML model expressing miR-155high and in these conditions evaluate syntenin expression in stromal cells.

R1. In **Figure 2,** our aim was to evaluate the effect of the lack of stromal syntenin on AML progression. This request of referee 2 is addressed in **Figure 1.** A wide range of publications in high impact journals have previously demonstrated that AML bearing FLT3-ITD mutation overexpresses this specific miR-155 (Wallace et al. 2017; Salemi et al. 2015; Gerloff et al. 2015; Hoang et al. 2022; Wang et al. 2023). **FIG. 1B** clearly demonstrates that the AML model expressing miR-155^{high} decrease the expression of syntenin in BMSCs *in vivo*.

Q2. Figure 5: Syntenin-deficiency in BMSC affects the distribution of endoglin. 5E. Authors should at least provide quantification of the confocal images. To better demonstrate the colocalization between endogenous endoglin and syntenin, they must perform proximity ligation assays.

R2. As requested, we now provide a Pearson quantification for syntenin-endoglin co-localization in HS5 cells (**Fig. 5E**, upper panel). The same experiment was also performed with another stromal cell line, HS27a (**Fig. 5E**, lower panel). To further demonstrate the co-localization between endogenous endoglin and syntenin, a proximity ligation assay was performed in HS5 stromal cells. This assay confirms the endogenous colocalization between endoglin and syntenin in the cytoplasm of HS5 cells (**Fig. EV1G**).

Q3. Figure 6: High endoglin expression in BMSC acts in trans to regulate AML protein synthesis. The mechanism(s) for how endoglin maintains AML cells proliferation and protein synthesis should be further elucidated. How to explain that when an invalidation for endoglin is carried out in panel 6B, the signal for pS473-AKT, pS235-RPS6 and EEF1A2 level increases whereas it was shown in panel A that under siRNA against endoglin the protein synthesis decreases.

R3. We acknowledge this observation and thank the referee for his remark. We now reproduced our results with two additional AML cell lines (U937 & OCI-AML3) and one other human stromal cell model (HS27a cells). We thereby confirm that syntenin deficiency in stromal cells markedly increases AML translational activity (**Fig. 6C** & **EV2C**), associated with an increase in EEF1A2 levels in the AML cells (**Fig. EV2D**). Co-down-regulating endoglin (in the stromal cells) prevents an effect of the stromal cells on protein synthesis and EEF1A2 levels in the AML. Altogether, these results indicate that high endoglin expression in BMSCs promotes AML protein synthesis associated with the activation of EEF1A2. Of note, we observed discrepancies in the phosphorylation of AKT and RPS6 depending on the AML model used, suggesting the signaling downstream of EEF1A2 may rely on alternative wirings and activation of other pathways than the AKT/RPS6. These observations are now also discussed.

Q4. Figure 7: Model recapitulating novel findings presented in this study. Authors must provide more mechanistic demonstration to support their model.

R4. The model summarizes the data that we have generated. We rephrased the legend to add more conditionals.

Q5. Finally, did authors considered the impact of overexpression of syntenin on AML development?

R5. Since we found that syntenin expression is downregulated in BMSC isolated from mice with leukemia (**Fig. 1**), our focus was on stromal-syntenin downregulation. Over-expression of scaffolding proteins (whereby all is about stoichiometry) is not without pitfalls and may also result in loss-of-function phenotypes. We are not convinced such over-expression experiment would necessarily help clarifyng the significance of a loss of expression experiment. The physiological relevance would also not be immediately clear to us.

Q6. Figure 2B: label on the axis indicates % of FLB1 cells in the peripheral blood at day 14 and contrasts with the title which indicates day15.

R6. Thank you for bringing this to our attention. We have made the necessary correction in the title, replacing "Day 15" with "Day 14."

Q7. In the text: Yet, FLB1 grown in syntenin-knockout mice significantly gained in aggressiveness from the 3rd transplantation on (Fig. 2B).

R7. Thank you for bringing this to our attention, it has been changed in the text.

REFEREE #3

The data from human models are limited. More human cell lines should be included.

This work shows that loss of the PDZ-domain containing protein Syntenin in bone marrow stromal cells (BMSC) accelerates AML in experimental systems in vitro and in vivo. Investigation of gene expression

datasets showed that experimentally induced AML models of different severity induce downregulation of Syntenin in the stromal compartment. This was correlated with expression levels of miR-155 in AML cells, and miR-155 downregulation reversed this effect. In transplantation experiments, loss of Syntenin in the host caused increased aggressiveness of transplanted murine leukemia cells in serial grafts. This was accompanied by activation of AKT, RPS6, elevated levels of Eef1a2 and increased protein synthesis. Similar observations were made in co-culture experiments of murine AML cells with wild-type vs. Syntenin-deficient BMSC as well as in co-cultures of the human AML cell line HL-60 with the HS5 stromal cell line in which Syntenin was silenced through RNA interference. Syntenin-deficient stromal cells exhibited increased surface expression of Endoglin, while Endoglin was depleted from small extracellular vesicles. Syntenin partially co-localized with Endoglin and downregulation of Endoglin in HS5 cells caused reduced protein synthesis in AML cells in co-cultures.

While this work provides evidence for the intriguing concept of stromal influence on AML pathophysiology, it has several shortcomings that compromise the relevance of the findings.

Q1. Most importantly, the general relevance of the results is not convincingly demonstrated. Most experiments are performed in one murine leukemia model and one human AML cell line. Not all results were conserved in U937 cells. Why could this be the case? Several human AML cell lines and different stromal cell lines should be included to show the general relevance of the findings.

R1. As requested, to strengthen the relevance of our results, another model of human BMSC (HS27a) and two additional human AML models (U937 and OCI-AML3) are now added to our functional assays. We confirm that syntenin deficiency markedly increases AML translational activity (**Fig. 6C** & **EV2C**), associated with an increase in EEF1A2 levels in AML cells (**Fig. EV2D**).

Q2. Data on syntenin gene expression in human AML stroma should be included.

R2. (Similar to last concern of referee 1).
To date very few clinical data exploring AML-BMSC have been generated. Indeed, BMSCs constitute a very limited population within the BM (and a fortiori the AML BM). Available databases do not reveal any dysregulation of syntenin expression within expanded AML-BMSCs compared to healthy donor-BMSC, but data were obtained from limited number of patients without any specifications (Von Der Heide et al. 2017). Although not currently applicable to routine work, new single cell omics approaches might allow setting up of a pipeline to analyze direct expression of syntenin (or that of agents, e.g. miRs that can downregulate syntenin) in BMSCs. Detecting the decrease of a syntenin transcript that normally is not abundant to start with, also presents a particular challenge. To address this issue indirectly and to so respond to this referee request, we tested whether aggressive forms of AML can upregulate the expression of endoglin at the surface of BMSCs in patients. Briefly, bone marrow samples isolated from AML FLT3-WT vs AML-FLT3-ITD patients were negatively selected for CD3, CD14, CD41, CD19, CD235a, CD45 & CD31. We then measured the membrane expression of endoglin in this population of 'stromal' cells (the term 'stromal' taken in the broad sense), when at least 300 stromal cells were reliably detected (see details in **Dataset EV4**). As shown in **Figure 6A** and in **Dataset EV4**, our results reveal that aggressive AML forms carrying the FLT3-ITD mutation significantly up-regulate endoglin expression on the surface of stromal cells. Although the patient cohort is limited (17 patients FLT3-WT vs. 8 patients FLT3-ITD), this result strongly suggests the clinical relevance of our findings in AML.

Q3. Can knockdown of EEF1A2 or Metarrestin treatment normalize the competitive advantage of Syntenin-deficient cells?

R3. In **appendix supplementary figure S3**, we show that the EEF1A2 inhibitor (Metarrestin) significantly reduces the *in vivo* progression of FLB1-G4KO cells that overexpress EEF1A2. In contrast, FLB1-G4WT cells that do not express EEF1A2 are unaffected by Metarrestin treatment.

Q4. Is the Metarrestin effect specific to Syntenin-deficient cells? FLB1-G4WT cells should be shown for comparison in Figure S3B.

R4. This condition was added in the manuscript. While very difficult to maintain *ex vivo*, FLB1-G4WT apoptosis is not significantly affected by a 24h treatment with 10µM of metarrestin (**Appendix Fig. S3C**).

Q5. Differentiation effects in Figure S4B should be quantified.

R5. As requested, the quantification was performed and added in appendix **Figure S4B**.

Q6. In contrast to what is written in the text and what is shown in Figure 6B, the data in Figure 6C do not show that silencing of Endoglin has opposing effects to silencing of Syntenin, as knockdowns of both genes caused increased AKT/RPS6 phosphorylation and EEF1A2 expression. The respective section in the results section should be rephrased.

R6. By including other AML models in our study, we now have more observations clarifying this issue. We demonstrate that co-silencing endoglin in stromal cells prevents the gain of expression of EEF1A2 in U937 and OCI-AML3 human AML cells. In HL60 AML cells, we observed the same trand although not significant (**Fig. EV2D**). Yet in HL60, it still holds that the knock-down of endoglin prevents a syntenin knockdown having an effect. These observations indicate that there are differences between the cellular models, suggesting the involvement of multiple mechanisms in the regulation of AML translation. This statement has been added to the discussion. See also reply to Q3 by referee 2.

REFERENCES

Gerloff, D., R. Grundler, A. A. Wurm, D. Bräuer-Hartmann, C. Katzerke, J. U. Hartmann, V. Madan, et al. 2015. “NF-KB/STAT5/MiR-155 Network Targets PU.1 in FLT3-ITD-Driven Acute Myeloid Leukemia.” *Leukemia* 29 (3): 535–47. https://doi.org/10.1038/leu.2014.231.

Heide, E. K. Von Der, M. Neumann, S. Vosberg, A. R. James, M. P. Schroeder, J. Ortiz-Tanchez, K. Isaakidis, et al. 2017. “Molecular Alterations in Bone Marrow Mesenchymal Stromal Cells Derived from Acute Myeloid Leukemia Patients.” *Leukemia* 31 (5): 1069–78. https://doi.org/10.1038/leu.2016.324.

Hoang, Dinh Hoa, Dandan Zhao, Sergio Branciamore, Davide Maestrini, Ivan R. Rodriguez, Ya Huei Kuo, Russell Rockne, et al. 2022. “MicroRNA Networks in FLT3-ITD Acute Myeloid Leukemia.” *Proceedings of the National Academy of Sciences of the United States of America* 119 (16). https://doi.org/10.1073/pnas.2112482119.

Salemi, Domenico, Giuseppe Cammarata, Cecilia Agueli, Luigi Augugliaro, Chiara Corrado, Maria Grazia Bica, Stefania Raimondo, et al. 2015. “MiR-155 Regulative Network in FLT3 Mutated Acute Myeloid Leukemia.” *Leukemia Research* 39 (8): 883–96. https://doi.org/10.1016/j.leukres.2015.04.017.

Wallace, Jared A., Dominique A. Kagele, Anna M. Eiring, Carissa N. Kim, Ruozhen Hu, Marah C. Runtsch, Margaret Alexander, et al. 2017. “MiR-155 Promotes FLT3-ITD-Induced Myeloproliferative Disease through Inhibition of the Interferon Response.” *Blood* 129 (23): 3074–86. https://doi.org/10.1182/blood-2016-09-740209.

Wang, Lingyan, Peifang Jiang, Jiazheng Li, Yan Huang, Jingjing Wen, Zhengjun Wu, Yanxin Chen, and Jianda Hu. 2023. “Loss of MiR-155 Sensitizes FLT3-ITD+AML to Chemotherapy and FLT3 Inhibitors via Glycolysis Blocking by Targeting PIK3R1.” *Journal of Cancer* 14: 99–113. https://doi.org/10.7150/jca.54775.

30th Aug 2023

Dear Dr. Leblanc,

Thank you for the submission of your revised manuscript to EMBO Molecular Medicine, and please accept my apologies for the delay in getting back to you in this busy time of the year. We have now received the reports from the 2 referees who re-reviewed your manuscript. As you will see, they are supportive of publication, and we will therefore be able to accept your manuscript once the following editorial points will be addressed:

1/ Main manuscript file:
- Please address the queries from our data editors in the related Data Edited manuscript file. Remove the yellow highlights in the text and only keep in track changes mode any new modification.
- We noted a minor discrepancy in an author's name: Berna Bou-Tayeh in the manuscript file, vs. Berna Boutayeh in the submission system.
- Materials and methods:
o We do not have size limitations on the Materials and Methods, so we would encourage you to add (part of) the supplementary methods currently in the appendix to the main manuscript file.
o Cells: please indicate whether the cells were authenticated and tested for mycoplasma contamination.
o Human material: please include the full statement that informed consent was obtained from all subjects and that the experiments conformed to the principles set out in the WMA Declaration of Helsinki and the Department of Health and Human Services Belmont Report.
o Antibodies: please provide dilutions/concentrations.
o Statistics: please include information on blinding, randomization, inclusion/exclusion criteria.
o Please make sure the checklist contains all above information.
- Data Availability: It is mandatory to include a 'Data Availability' section after the Materials and Methods and before the Acknowledgements. Primary datasets produced in this study need to be deposited in an appropriate public database, and the accession numbers and database listed under 'Data Availability'. In case you have no data that requires deposition in a public database, please state so in this section ("This study includes no data deposited in external repositories"). Note that the Data Availability Section is restricted to new primary data that are part of this study.
- Acknowledgements: All funding sources should be added to the submission system, and match the information provided in the manuscript (currently missing: the Institut National de la Santé et de la Recherche Médicale (INSERM), Institute for Cancer and Immunology (Aix-Marseille University)).
- Author contributions: CRediT has replaced the traditional author contributions section because it offers a systematic machine-readable author contributions format that allows for more effective research assessment. Please remove the Authors Contributions from the manuscript and use the free text boxes beneath each contributing author's name in our system to add specific details on the author's contribution. More information is available in our guide to authors.
- Please rename to the conflict of interest to "Disclosure statement and competing interests". We updated our journal's competing interests policy and request authors to consider both actual and perceived competing interests. Please review the policy https://www.embopress.org/competing-interests and update your competing interests if necessary. This section should be removed from the title page and placed after the Acknowledgements.
- References: please list 10 authors before et al., and only add DOIs for preprints and datasets not yet published.

2/ Figures and Appendix:
- Please upload each figure (main and EV) as an individual figure file.
- Please make sure all figures are correctly referenced in the manuscript file. 'Appendix Supplementary Table S1' should be renamed to 'Appendix Table S1'.
- Please combine the different Appendix files into one single file with a Table of Content. The Appendix figure legends should be in the same file.
- The Dataset EV legends should be removed from the manuscript and included in each Excel file as a separate tab.
- Reagent and Tools table: please format it as indicated in our guide to authors, you can find a template here: https://www.embopress.org/page/journal/17574684/authorguide

3/ Source Data:
Source Data for the main figures need to be uploaded 1 folder/file per 1 figure while the SD for EV and Appendix figures can be grouped and uploaded as 1 folder; Please provide the links to PRIDE.

4/ The paper explained: EMBO Molecular Medicine articles are accompanied by a summary of the articles to emphasize the major findings in the paper and their medical implications for the non-specialist reader. Please provide a summary of your article highlighting
- the medical issue you are addressing,
- the results obtained and

- their clinical impact.
This may be edited to ensure that readers understand the significance and context of the research. Please refer to any of our published articles for an example.

5/ Every published paper now includes a 'Synopsis' to further enhance discoverability. Synopses are displayed on the journal webpage and are freely accessible to all readers. They include a short stand first (maximum of 300 characters, including space) as well as 2-5 one-sentences bullet points that summarizes the paper. Please write the bullet points to summarize the key NEW findings. They should be designed to be complementary to the abstract - i.e. not repeat the same text. We encourage inclusion of key acronyms and quantitative information (maximum of 30 words / bullet point). Please use the passive voice. Please attach these in a separate file or send them by email, we will incorporate them accordingly.

Please also suggest a striking image or visual abstract to illustrate your article as a PNG file 550 px wide x 300-600 px high.

6/ As part of the EMBO Publications transparent editorial process initiative (see our Editorial at http://embomolmed.embopress.org/content/2/9/329), EMBO Molecular Medicine will publish online a Review Process File (RPF) to accompany accepted manuscripts.
This file will be published in conjunction with your paper and will include the anonymous referee reports, your point-by-point response and all pertinent correspondence relating to the manuscript. Let us know whether you agree with the publication of the RPF and as here, if you want to remove or not any figures from it prior to publication.
Please note that the Authors checklist will be published at the end of the RPF.

I look forward to receiving your revised manuscript.

Yours sincerely,

Lise Roth

Lise Roth, PhD
Senior Editor
EMBO Molecular Medicine

***** Reviewer's comments *****

Referee #2 (Remarks for Author):

The authors have sufficiently addressed my comments. The manuscript is significantly improved compared to the original submission.

Referee #3 (Remarks for Author):

The authors have sufficiently responded to my questions. The inclusion of results from more cell line models strengthens the work.

The authors addressed the minor issues.

11th Sep 2023

Dear Dr. Leblanc,

Thank you for submitting your revised files. I am pleased to inform you that I will be able to accept your manuscript once the following editorial issues are addressed:

- Please address the queries from our data editors in the related Data Edited manuscript file. In case you did not find this file, I attach it to this letter for your convenience.
- Please correct the checklist to make sure it reflects the information provided in the manuscript (specifically, information about blinding and inclusion/exclusion criteria, and right section of data availability/datasets).
- Data Availability: The datasets deposited must be publicly accessible before online publication of your manuscript, and a URL link must be provided.
- Please make sure all figures are correctly referenced in the manuscript file. 'Appendix Supplementary Table S1' should be renamed to 'Appendix Table S1' (corrections are still needed on p.13 and 16 and in the legends of the appendix file).
- Thank you for providing The Paper Explained, please incorporate it in the manuscript.

Once you have addressed the above, please accept all changes and only keep in track changes mode any new modification.

As part of the EMBO Publications transparent editorial process initiative (see our Editorial at http://embomolmed.embopress.org/content/2/9/329), EMBO Molecular Medicine will publish online a Review Process File (RPF) to accompany accepted manuscripts.
This file will be published in conjunction with your paper and will include the anonymous referee reports, your point-by-point response and all pertinent correspondence relating to the manuscript.
Please Let us know whether you agree with the publication of the RPF and as here, if you want to remove or not any figures from it prior to publication.
Please note that the Authors checklist will be published at the end of the RPF.

Thank you for bearing with these last issues.
I look forward to receiving your revised manuscript.

With kind regards,

Lise Roth

Lise Roth, PhD
Senior Editor
EMBO Molecular Medicine

The authors addressed the remaining editorial issues.

14th Sep 2023

Dear Dr. Leblanc,

I am pleased to inform you that your manuscript is accepted for publication and is now being sent to our publisher to be included in the next available issue of EMBO Molecular Medicine!

We would like to remind you that as part of the EMBO Publications transparent editorial process initiative, EMBO Molecular Medicine will publish a Review Process File online to accompany accepted manuscripts. If you do NOT want the file to be published or would like to exclude figures, please immediately inform the editorial office via e-mail.

Congratulations on your interesting work,

With kind regards,

Lise Roth

Lise Roth, Ph.D
Senior Editor
EMBO Molecular Medicine

Follow us on Twitter @EmboMolMed
Sign up for eTOCs at embopress.org/alertsfeeds

*** *** *** IMPORTANT INFORMATION *** *** ***

SPEED OF PUBLICATION
The journal aims for rapid publication of papers, using using the advance online publication "Early View" to expedite the process: A properly copy-edited and formatted version will be published as "Early View" after the proofs have been corrected. Please help the Editors and publisher avoid delays by providing e-mail address(es), telephone and fax numbers at which author(s) can be contacted.

Should you be planning a Press Release on your article, please get in contact with embomolmed@wiley.com as early as possible, in order to coordinate publication and release dates.

LICENSE AND PAYMENT:

All articles published in EMBO Molecular Medicine are fully open access: immediately and freely available to read, download and share.

EMBO Molecular Medicine charges an article processing charge (APC) to cover the publication costs. You, as the corresponding author for this manuscript, should have already received a quote with the article processing fee separately. Please let us know in case this quote has not been received.

Once your article is at Wiley for editorial production you will receive an email from Wiley's Author Services system, which will ask you to log in and will present you with the publication license form for completion. Within the same system the publication fee can be paid by credit card, an invoice, pro forma invoice or purchase order can be requested.

Payment of the publication charge and the signed Open Access Agreement form must be received before the article can be published online.

PROOFS

You will receive the proofs by e-mail approximately 2 weeks after all relevant files have been sent o our Production Office. Please return them within 48 hours and if there should be any problems, please contact the production office at embopressproduction@wiley.com.

Please inform us if there is likely to be any difficulty in reaching you at the above address at that time. Failure to meet our deadlines may result in a delay of publication.

All further communications concerning your paper proofs should quote reference number EMM-2023-17570-V4 and be directed to the production office at embopressproduction@wiley.com.

EMBO Press Author Checklist

Corresponding Author Name: Leblanc & Zimmermann	
Journal Submitted to: Embo Molecular Medicine	
Manuscript Number: EMM-2013-17570	

USEFUL LINKS FOR COMPLETING THIS FORM
The EMBO Journal - Author Guidelines
EMBO Reports - Author Guidelines
Molecular Systems Biology - Author Guidelines
EMBO Molecular Medicine - Author Guidelines

Reporting Checklist for Life Science Articles (updated January
This checklist is adapted from Materials Design Analysis Reporting (MDAR) Checklist for Authors. MDAR establishes a minimum set of requirements in transparent reporting in the life sciences (see Statement of Task: 10.31222/osf.io/9sm4x). Please follow the journal's guidelines in preparing your
Please note that a copy of this checklist will be published alongside your article.

Abridged guidelines for figures
1. Data
The data shown in figures should satisfy the following conditions:
- the data were obtained and processed according to the field's best practice and are presented to reflect the results of the experiments in an accurate and unbiased manner.
- ideally, figure panels should include only measurements that are directly comparable to each other and obtained with the same assay.
- plots include clearly labeled error bars for independent experiments and sample sizes. Unless justified, error bars should not be shown for technical
- if n<5, the individual data points from each experiment should be plotted. Any statistical test employed should be justified.
- Source Data should be included to report the data underlying figures according to the guidelines set out in the authorship guidelines on Data

2. Captions
Each figure caption should contain the following information, for each panel where they are relevant:
- a specification of the experimental system investigated (eg cell line, species name).
- the assay(s) and method(s) used to carry out the reported observations and measurements.
- an explicit mention of the biological and chemical entity(ies) that are being measured.
- an explicit mention of the biological and chemical entity(ies) that are altered/varied/perturbed in a controlled manner.
- the exact sample size (n) for each experimental group/condition, given as a number, not a range;
- a description of the sample collection allowing the reader to understand whether the samples represent technical or biological replicates (including how many animals, litters, cultures, etc.).
- a statement of how many times the experiment shown was independently replicated in the laboratory.
- definitions of statistical methods and measures:
  - common tests, such as t-test (please specify whether paired vs. unpaired), simple χ2 tests, Wilcoxon and Mann-Whitney tests, can be unambiguously identified by name only, but more complex techniques should be described in the methods section;
  - are tests one-sided or two-sided?
  - are there adjustments for multiple comparisons?
  - exact statistical test results, e.g., P values = x but not P values < x;
  - definition of 'center values' as median or average;
  - definition of error bars as s.d. or s.e.m.

Please complete ALL of the questions below.
Select "Not Applicable" only when the requested information is not relevant for your study.

Materials

Newly Created Materials	Information included in the manuscript?	In which section is the information available? (Reagents and Tools Table, Materials and Methods, Figures, Data Availability Section)
New materials and reagents need to be available; do any restrictions apply?	Not Applicable	

Antibodies	Information included in the manuscript?	In which section is the information available? (Reagents and Tools Table, Materials and Methods, Figures, Data Availability Section)
For **antibodies** provide the following information:<br>- Commercial antibodies: RRID (if possible) or supplier name, catalogue number and or/clone number<br>- Non-commercial: RRID or citation	Yes	Appendix Table S1

DNA and RNA sequences	Information included in the manuscript?	In which section is the information available? (Reagents and Tools Table, Materials and Methods, Figures, Data Availability Section)
**Short novel DNA or RNA including primers, probes**: provide the sequences.	Yes	Appendix Table S2

Cell materials	Information included in the manuscript?	In which section is the information available? (Reagents and Tools Table, Materials and Methods, Figures, Data Availability Section)
**Cell lines:** Provide species information, strain. Provide accession number in repository **OR** supplier name, catalog number, clone number, and/**OR** RRID.	Yes	Materials and methods
**Primary cultures:** Provide species, strain, sex of origin, genetic modification status.	Yes	Materials and methods
Report if the cell lines were recently **authenticated** (e.g., by STR profiling) and tested for mycoplasma contamination.	Yes	Since mycoplasma affect exosomal secretion, our cell lines are monthly tested for mycoplasma. Indicated in Materials and methods

Experimental animals	Information included in the manuscript?	In which section is the information available? (Reagents and Tools Table, Materials and Methods, Figures, Data Availability Section)
**Laboratory animals or Model organisms:** Provide species, strain, sex, age, genetic modification status. Provide accession number in repository **OR** supplier name, catalog number, clone number, **OR** RRID.	Yes	Materials and methods
**Animal observed in or captured from the field:** Provide species, sex, and age where possible.	Not Applicable	
Please detail **housing and husbandry conditions**.	Yes	Materials and methods

Plants and microbes	Information included in the manuscript?	In which section is the information available? (Reagents and Tools Table, Materials and Methods, Figures, Data Availability Section)
**Plants:** provide species and strain, ecotype and cultivar where relevant, unique accession number if available, and source (including location for collected wild specimens).	Not Applicable	
**Microbes:** provide species and strain, unique accession number if available, and source.	Not Applicable	

Human research participants	Information included in the manuscript?	In which section is the information available? (Reagents and Tools Table, Materials and Methods, Figures, Data Availability Section)
If collected and within the bounds of privacy constraints report on age, sex and gender or ethnicity for all study participants.	Yes	Materials and Methods and details in dataset EV4

Core facilities	Information included in the manuscript?	In which section is the information available? (Reagents and Tools Table, Materials and Methods, Figures, Data Availability Section)
If your work benefited from core facilities, was their service mentioned in the acknowledgments section?	Yes	Aknowledgments section

Design

Study protocol	Information included in the manuscript?	In which section is the information available? (Reagents and Tools Table, Materials and Methods, Figures, Data Availability Section)
If study protocol has been **pre-registered, provide DOI in the manuscript**. For clinical trials, provide the trial registration number **OR** cite DOI.	Not Applicable	
Report the **clinical trial registration number** (at ClinicalTrials.gov or equivalent), where applicable.	Not Applicable	

Laboratory protocol	Information included in the manuscript?	In which section is the information available? (Reagents and Tools Table, Materials and Methods, Figures, Data Availability Section)
Provide DOI OR other citation details if **external detailed step-by-step protocols** are available.	Not Applicable	

Experimental study design and statistics	Information included in the manuscript?	In which section is the information available? (Reagents and Tools Table, Materials and Methods, Figures, Data Availability Section)
Include a statement about **sample size** estimate even if no statistical methods were used.	Yes	Figures legends
Were any steps taken to minimize the effects of subjective bias when allocating animals/samples to treatment (e.g. **randomization procedure**)? If yes, have they been described?	Yes	Not described. The animals were divided into different groups of 5 in order to avoid the "cage effect" for all animal experimentations
Include a statement about **blinding** even if no blinding was done.	Not Applicable	Materials and methods
Describe **inclusion/exclusion criteria** if samples or animals were excluded from the analysis. Were the criteria pre-established?<br><br>If sample or data points were omitted from analysis, report if this was due to attrition or intentional exclusion and provide justification.	Yes	For human samples, the membrane expression of endoglin stromal cells were measured when at least 300 stromal cells were reliably detected. Indicated in Materials and methods, in particular in the section "statistical analysis"
For every figure, are **statistical tests** justified as appropriate? Do the data meet the assumptions of the tests (e.g., normal distribution)? Describe any methods used to assess it. Is there an estimate of variation within each group of data? Is the variance similar between the groups that are being statistically compared?	Yes	Materials and methods, Figure legends

Sample definition and in-laboratory replication	Information included in the manuscript?	In which section is the information available? (Reagents and Tools Table, Materials and Methods, Figures, Data Availability Section)
In the figure legends: state number of times the experiment was **replicated** in laboratory.	Yes	Figure legends
In the figure legends: define whether data describe **technical or biological replicates**.	Yes	Figure legends

Ethics

Ethics	Information included in the manuscript?	In which section is the information available? (Reagents and Tools Table, Materials and Methods, Figures, Data Availability Section)
Studies involving **human participants**: State details of **authority granting ethics approval** (IRB or equivalent committee(s), provide reference number for approval.	Yes	Materials and methods
Studies involving **human participants**: Include a statement confirming that **informed consent** was obtained from all subjects and that the experiments conformed to the principles set out in the WMA Declaration of Helsinki and the Department of Health and Human Services Belmont Report.	Yes	Materials and methods
Studies involving **human participants:** For publication of **patient photos**, include a statement confirming that consent to publish was obtained.	Not Applicable	
Studies involving experimental **animals**: State details of **authority granting ethics approval** (IRB or equivalent committee(s), provide reference number for approval. Include a statement of compliance with ethical regulations.	Yes	Materials and methods
Studies involving **specimen and field samples:** State if relevant **permits** obtained, provide details of authority approving study; if none were required, explain why.	Not Applicable	

Dual Use Research of Concern (DURC)	Information included in the manuscript?	In which section is the information available? (Reagents and Tools Table, Materials and Methods, Figures, Data Availability Section)
Could your study fall under dual use research restrictions? Please check biosecurity documents and list of **select agents and toxins** (CDC): https://www.selectagents.gov/sat/list.htm	Not Applicable	
If you used a select agent, is the security level of the lab appropriate and reported in the manuscript?	Not Applicable	
If a study is subject to dual use research of concern regulations, is the name of the **authority granting approval and reference number** for the regulatory approval provided in the manuscript?	Not Applicable	

Reporting

The MDAR framework recommends adoption of discipline-specific guidelines, established and endorsed through community initiatives. Journals have their own policy about requiring specific guidelines and recommendations to complement MDAR.

Adherence to community standards	Information included in the manuscript?	In which section is the information available? (Reagents and Tools Table, Materials and Methods, Figures, Data Availability Section)
State if relevant guidelines or checklists (e.g., **ICMJE, MIBBI, ARRIVE, PRISMA**) have been followed or provided.	Not Applicable	
For **tumor marker prognostic studies,** we recommend that you follow the **REMARK** reporting guidelines (see link list at top right). See author guidelines, under 'Reporting Guidelines'. Please confirm you have followed these guidelines.	Not Applicable	
For **phase II and III randomized controlled trials**, please refer to the **CONSORT** flow diagram (see link list at top right) and submit the CONSORT checklist (see link list at top right) with your submission. See author guidelines, under 'Reporting Guidelines'. Please confirm you have submitted this list.	Not Applicable	

Data Availability

Data availability	Information included in the manuscript?	In which section is the information available? (Reagents and Tools Table, Materials and Methods, Figures, Data Availability Section)
Have **primary datasets** been deposited according to the journal's guidelines (see 'Data Deposition' section) and the respective accession numbers provided in the Data Availability Section?	Yes	Proteomic dataset is publicly available (https://www.ebi.ac.uk/pride/archive/projects/PXD023602). Indicated in Materials and methods and in Data Availibility Section
Were **human clinical and genomic datasets** deposited in a public access-controlled repository in accordance to ethical obligations to the patients and to the applicable consent agreement?	Yes	Materials and methods
Are **computational models** that are central and integral to a study available without restrictions in a machine-readable form? Were the relevant accession numbers or links provided?	Not Applicable	
If publicly available data were reused, provide the respective **data citations in the reference list**.	Yes	Materials and methods, results
